# Structural and bioinformatics analyses identify deoxydinucleotide-specific nucleases and their association with genomic islands in gram-positive bacteria

**DOI:** 10.1093/nar/gkae1235

**Published:** 2025-01-08

**Authors:** Sofia Mortensen, Stanislava Kuncová, Justin D Lormand, Tanner M Myers, Soo-Kyoung Kim, Vincent T Lee, Wade C Winkler, Holger Sondermann

**Affiliations:** CSSB Centre for Structural Systems Biology, Deutsches Elektronen-Synchrotron DESY, Notkestr. 85, 22607 Hamburg, Germany; CSSB Centre for Structural Systems Biology, Deutsches Elektronen-Synchrotron DESY, Notkestr. 85, 22607 Hamburg, Germany; CSSB Centre for Structural Systems Biology, Deutsches Elektronen-Synchrotron DESY, Notkestr. 85, 22607 Hamburg, Germany; Department of Chemistry and Biochemistry, University of Maryland, College Park, MD 20742, USA; Department of Cell Biology and Molecular Genetics, University of Maryland, College Park, MD 20742, USA; Department of Cell Biology and Molecular Genetics, University of Maryland, College Park, MD 20742, USA; Department of Chemistry and Biochemistry, University of Maryland, College Park, MD 20742, USA; Department of Cell Biology and Molecular Genetics, University of Maryland, College Park, MD 20742, USA; CSSB Centre for Structural Systems Biology, Deutsches Elektronen-Synchrotron DESY, Notkestr. 85, 22607 Hamburg, Germany; Christian-Albrechts-University, 24118 Kiel, Germany

## Abstract

Dinucleases of the DEDD superfamily, such as oligoribonuclease, Rexo2 and nanoRNase C, catalyze the essential final step of RNA degradation, the conversion of di- to mononucleotides. The active sites of these enzymes are optimized for substrates that are two nucleotides long, and do not discriminate between RNA and DNA. Here, we identified a novel DEDD subfamily, members of which function as dedicated deoxydinucleases (diDNases) that specifically hydrolyze single-stranded DNA dinucleotides in a sequence-independent manner. Crystal structures of enzyme-substrate complexes reveal that specificity for DNA stems from a combination of conserved structural elements that exclude diribonucleotides as substrates. Consistently, diDNases fail to complement the loss of enzymes that act on diribonucleotides, indicating that these two groups of enzymes support distinct cellular functions. The genes encoding diDNases are found predominantly in genomic islands of Actinomycetes and Clostridia, which, together with their association with phage-defense systems, suggest potential roles in bacterial immunity.

## Graphical Abstract

**Graphical Abstract:**
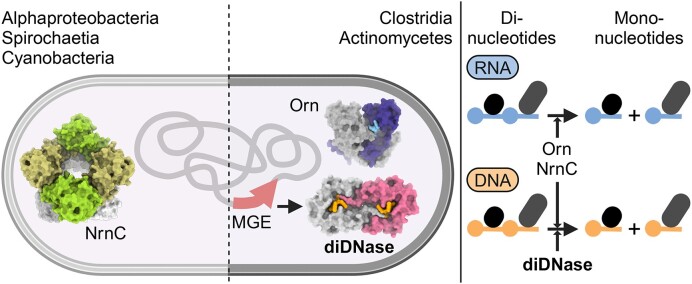

## Introduction

Nucleic acid cleavage is a vital process in cellular physiology as it is essential for RNA processing and turnover, DNA replication, DNA polymerase proofreading activity, DNA repair, recombination, antiviral defense and signaling. Consistent with a wide array of biological functions, a variety of nucleases are present in cells and can be broadly classified into endonucleases and exonucleases, depending on whether they hydrolyze phosphodiester bonds internal to long nucleic acids or from the free ends of a substrate, respectively (1,2). Alternatively, nucleases can be distinguished based on the conservation and fold of their catalytic domains. For example, nucleases of the DEDD superfamily adopt a common DnaQ fold (3). The four catalytic residues, the DEDD signature that gives the superfamily its name, are strictly conserved and belong to three motifs: EXOI with the first aspartate and glutamate residues of the DEDD signature, EXOII with the second aspartate residue and EXOIII with the last aspartate residue (3,4). Cleavage of phosphodiester bonds in nucleic acids requires an intact DEDD signature, as these residues coordinate two divalent cations that are important for the general reaction mechanism (5). Variation in the EXOI-III motifs beyond the acidic catalytic residues can be used to separate DEDD family proteins into subclasses with distinct substrate preferences (4). However, the classification of nucleases can be complicated by enzymes having broader or more unique substrate preferences and by catalytic activities and substrate specificity of some nucleases varying depending on the experimental conditions (5).

DEDD superfamily proteins may consist of a single domain, such as RNase T, an enzyme involved in the maturation of tRNA and rRNA (6). There are also examples of multidomain DEDD proteins, such as RNase D and DNA polymerases (7,8). Structurally related yet evolutionarily distinct exoribonuclease oligoribonuclease (Orn) and nanoRNase C (NrnC) are single-domain DEDD proteins that fulfill an essential housekeeping function during the final step of RNA degradation (9–11). In addition, Orn is central to nucleotide-based second messenger signaling as it breaks down linear dinucleotides generated by cyclic dinucleotide-specific phosphodiesterases (12,13). We recently showed that, despite their original categorization as oligo- or nanoRNases, Orn and NrnC function as dedicated dinucleases with high specificity toward substrates of two nucleotides in length under near-physiological conditions (12,14,15).

Orn and NrnC dinucleases fall into separate classes within the DEDD family as based on the fifth catalytic residue in their EXOIII motifs, a histidine (h) in Orn (DEDDh subclass) and a tyrosine (y) in NrnC (DEDDy subclass) (16). Additionally, Orn functions as a homodimer, whereas NrnC forms a homooctamer in crystals and in solution (14,15,17). Regardless of these sequence and structural differences, Orn and NrnC share remarkably similar active site architectures, which have evolved convergently and define them as dinucleases. The active sites invariably consist of (i) a catalytic DEDDh/y-containing core for divalent cation coordination and catalysis, (ii) a leucine residue that wedges between the two bases of the substrates and (iii) a 5′-phosphate cap that coordinates the 5′ phosphate of the dinucleotides (15).

In our previous studies, we examined the phylogenetic distribution of NrnC homologs and discovered that they are mainly present in three clades of bacteria: Alphaproteobacteria, Cyanobacteria, and Actinomycetes (15). Although Alphaproteobacterial and Cyanobacterial NrnC homologs share a high degree of sequence similarity with the NrnC homologs investigated to date, Actinomycetal NrnC homologs are more divergent and include a change in key residues in the binding pocket of the 5′ phosphate (15). Whether the apparent sequence divergence translates into alternative substrate preferences or functions of NrnC homologs in Actinomycetes remained unknown.

Here, we report that NrnC homologs from Actinomycetes and Clostridia differ from the NrnC proteins studied thus far in their oligomeric states and enzymatic functions. In particular, we show that Actinomycetal and Clostridial NrnC homologs are dimeric enzymes that selectively hydrolyze deoxydinucleotides. Crystal structures of enzyme-substrate complexes show that substrate specificity is achieved through a combination of structural elements surrounding the active site of the enzyme. Actinomycetes with diDNases also encode Orn proteins, which do not display any apparent functional differences from Gammaproteobacterial Orn *in vitro* and *in vivo*. Therefore, Orn proteins are likely to support housekeeping functions during RNA degradation. In contrast, diDNase-encoding genes have a spurious phylogenetic distribution and are associated with mobile genetic elements (MGE) and known phage-defense genes, suggesting possible roles in bacterial immunity.

## Material and methods

### Phylogenetic analysis of NrnC homologs

Protein sequences of NrnC homologs were identified using BLASTp against nonredundant protein sequences (18). One representative sequence was selected from each of the previously identified bacterial orders containing NrnC (15). The final set included 46 sequences (Supplementary Table S1). Selected NrnC protein sequences met the following basic criteria: single-domain proteins of approximately 200 amino acids and containing a catalytic DEDDy signature. Sequences were aligned using Clustal Omega (19) and a phylogenetic tree was built using Simple Phylogeny (19). Sequence logos were generated with WebLogo3 (20). Protein identifier, organism of origin, and GenBank locus for the enzymes investigated experimentally are listed in Supplementary Table S1.

### Protein expression and purification

NrnC coding sequences were codon-optimized for expression in *E. coli*, gene synthesized and cloned into a modified pET28a vector (GenScript, Novagen) to generate a His_6_-tagged small ubiquitin-like modifier (SUMO) N-terminal fusion. Single point mutants were generated using the QuikChange Site-Directed Mutagenesis Kit (Agilent) according to manufacturer instructions. For protein expression, plasmids were transformed into One Shot BL21(DE3) cells (Invitrogen) and grown for 2 h at 37°C, then at 18°C for 18–20 h in auto-induction (AI) media ZYM-5052 (21) supplemented with 100 μg/ml kanamycin.

Cells were lysed by sonication in lysis buffer (50 mM Tris-HCl, 500 mM NaCl, 25 mM imidazole, 1 mM PMSF and 3 mM β-mercaptoethanol [pH 8.5]). Clarified lysates were incubated with Ni-NTA resin (Qiagen) and washed with lysis buffer. His_6_-tagged Ulp1 protease was added to the Ni-NTA resin to cleave the His_6_-SUMO tag and elute the untagged proteins of interest. Centrifugal filters (Amicon Ultra-4, 10 kDa cut-off) were used to concentrate proteins, then EDTA was added (pH 8.5, 10 mM final). Proteins were further purified by gel filtration using either HiLoad 16/600 Superdex 200 pg or Superdex 200 Increase 10/300 GL columns (Cytiva) equilibrated with gel filtration buffer (25 mM HEPES-NaOH and 150 mM NaCl [pH 7.5]). Protein-containing fractions were flash-frozen in liquid nitrogen, and stored at −70°C.

### Size-exclusion chromatography-coupled multi-angle light scattering (SEC-MALS)

Purified proteins at 1–2 mg/ml (∼40–80 μM) were injected onto a Superdex 200 Increase 10/300 GL column (Cytiva) equilibrated with gel filtration buffer at room temperature and coupled with a static multi-angle light scattering detector (miniDAWN, Wyatt Technology, Waters) and a refractive index detector (Optilab T-rEX, Wyatt Technology, Waters). Astra software (version 8.0.2.5, Wyatt Technology, Waters) was used for data analysis.

### Nanoscale differential scanning fluorimetry (nDSF)

Nanoscale differential scanning fluorimetry (nDSF) was performed on a Prometheus NT.48 (NanoTemper Technologies, Munich, Germany) (22). Proteins (0.5–1 mg/ml in gel filtration buffer) were incubated with 0.5 mM GMP (Sigma), dGTP (Sigma), pGG (Jena Bioscience), deoxy-pGG (Dharmacon), pCGG (Dharmacon) or deoxy-pAGG (Dharmacon) for 5 min at 20°C, then analyzed with a temperature scan from 20°C to 95°C. Data were processed using MoltenProt on the eSPC server (23,24).

### Enzyme kinetics

Substrate hydrolysis by NrnC homologs was measured by fluorescence unquenching of nucleotides containing 2-aminopurine as one of the bases (25). All substrates were purchased 5′-phosphorylated from Dharmacon. Enzymatic activity was measured as the rate of increase in the fluorescence intensity of cleaved 2-aminopurine with excitation at 310 nm and emission at 375 nm over a series of initial substrate concentrations (0.9–45 μM). Enzyme concentrations ranged from 10 to 500 nM, depending on the level of activity, as indicated in results. The substrates used are p(2AP)G in either RNA or DNA form, meaning with the dinucleotides containing either ribose or deoxyribose, respectively, unless stated otherwise. The reactions were assembled by mixing the reaction buffer (20 mM Tris-HCl, 150 mM KCl [pH 7.9]) with a substrate stock prepared in water and an enzyme diluted with dilution buffer (20 mM Tris-HCl, 150 mM KCl, 4 mM EDTA [pH 7.9]). 45 μL of the resulting mixtures were placed in the wells of a 96 well plate and reactions were started by adding 5 μL of start buffer (20 mM Tris-HCl, 150 mM KCl, 100 mM MgCl_2_ [pH 7.9]). The fluorescence intensity was recorded in triplicate every 10 s for 3 min at 21°C using a Tecan Infinite M Plex plate reader. The initial slopes of the resulting time-course data were determined and converted into the velocities of product release using a calibration curve. Calibration curves were created for each type of substrate tested by incubating the reactions for 5–10 hours in a sealed plate to allow for complete substrate conversion and plotting the final fluorescence intensity values against initial substrate concentrations. The velocities of product release were plotted against initial substrate concentrations and fitted with enzyme kinetics models in GraphPad Prism 10.

### Oligonucleotide radioactive labeling and cleavage reactions

Single-stranded DNA fragments (purchased from Integrated DNA Technologies) were phosphorylated with equimolar concentrations of [γ-^32^P]-ATP and T4 PNK in T4 PNK Reaction Buffer for 1 h at 37°C, followed by heat inactivation at 95°C for 5 min. 5′-^32^P-radiolabeled DNA was annealed with complementary DNA to form 5′-overhang, 3′-overhang or blunt-end DNA by incubation at 95°C for 10 min followed by cooling at room temperature for 1 h. The cleavage reactions were run at room temperature in 20 mM Tris-HCl, 150 mM KCl, 5 mM MgCl_2_ [pH 7.9] with 3.3 nM of the DNA substrates and the indicated enzyme concentrations. At the indicated times (0, 3, 5, 10, 20, 30 min), the reactions were stopped by transferring 2 μl of the reaction to tubes containing 5 μl of 0.2 M EDTA. Samples were analyzed by 20% Urea PAGE in 1x TBE buffer. The gel was exposed to a phosphor imager screen and imaged using a Fujifilm FLA-7000 phosphor imager (GE). Experiments were performed in triplicates and representative gels are shown.

### Crystallization and structure determination

NrnC homologs were incubated with either pGG (Jena Bioscience) or deoxy-pGG (dpGG, Dharmacon) at 1:2 protein:substrate ratio prior to crystallization. Crystals were obtained in sitting-drop setups at 19°C using the following conditions: apo-diDNase*_Rho_* - 18%(w/v) PEG3350, 0.1 M sodium acetate (pH 4.5), 0.1 M Bis-Tris (pH 5.5); diDNase*_Rho_*-dpGG - 0.2 M ammonium chloride, 20%(w/v) PEG3350; diDNase*_Noc_*-dpGG - 0.2 M lithium sulfate, 0.1 M bis-tris (pH 5.5), 25%(w/v) PEG3350; NrnC*_Bh_*-dpGG - 0.1 M succinic acid (pH 6.5), 20% PEG 3350, and 20% xylitol; Orn*_Bla_*-pGG - 0.2 M sodium chloride, 0.1 M Bis-Tris (pH 5.5), 25%(w/v) PEG3350. Crystals were cryoprotected with xylitol or low-viscosity oil prior to flash-cooling in liquid nitrogen.

Diffraction data were collected at beamline P11 at the Deutsches Elektronen-Synchrotron (DESY) and processed using XDS (26). Phase information was obtained by molecular replacement using Phaser (27) and search models based on NrnC*_Bh_* (PDB ID 7MPL) or Orn*_Vc_* (PDB ID 6N6A) structures. Models were iteratively refined using Phenix.refine (28) and manually built in Coot (29). Structural figures were prepared using PyMOL (version 2.5.0; Schrödinger). Data processing and model refinement statistics are presented in Supplementary Table S2. The graphical abstract was created in BioRender (Sondermann, H. (2024), https://BioRender.com/m30c846).

### *P. aeruginosa Δorn* complementation assay

Genes of interest were subcloned into a modified pJN105 vector (30) for expression of N-terminal StrepII-tagged proteins in *P. aeruginosa Δorn*. Plasmids were introduced into *P. aeruginosa* by electroporation (15). Drip assays were performed as previously described on LB agar plates containing 60 μg/ml gentamicin and 0.2% arabinose (15). Colony area was quantified using particle analysis in ImageJ (31).

### *P. aeruginosa* growth curves

Single colonies of *P. aeruginosa PA14* transformants with either empty vector (pJN105) or pJN105 encoding for diDNase*_Noc_* were used to inoculate LB supplemented with 60 μg/ml gentamicin, followed by growth overnight at 37°C with shaking. Overnight cultures were diluted with fresh LB containing 60 μg/ml gentamicin and 0.2% arabinose to an OD_600_ of 0.05 and grown at 37°C with shaking. OD_600_ was measured at indicated time intervals. Data are plotted as average values from three experiments with error bars representing standard deviations.

### Western blotting

*P. aeruginosa* expressing StrepII-tagged NrnC homologs from a plasmid were cultured in LB supplemented with 60 μg/ml gentamicin and 0.2% arabinose overnight at 37°C. Cells (1 ml) were collected, resuspended in SDS-PAGE loading buffer, incubated at 100°C for 10 min and resolved on 15% SDS-PAGE gels. Proteins were transferred onto 0.2 μm PVDF membranes using a Trans-Blot Turbo Transfer System (Bio-Rad), blocked with EveryBlot Blocking Buffer (Bio-Rad) for 1 h at 20°C and incubated with StrepTag II HRP antibody (Millipore, Merck, at a 1:10000 dilution) for an additional hour at 20°C. Membranes were washed three times for 5 min with PBST and imaged with Amersham ECL Select Western Blotting Detection Reagent (Cytiva).

### *B. subtilis ΔnrnAΔnrnB* lysate experiments

*B. subtilis ΔnrnAΔnrnB* strain was previously described (12). Sequences for Orn*_Vc_*, NrnC*_Bh_*, or diDNase*_Noc_* were subcloned into pDR111 before integration into the non-essential amyE locus through double homologous recombination as previously described (32). Overnight *B. subtilis* cultures grown at 37°C were diluted to an OD_600_ of ∼ 1.0 and cultured for 40 min in 250 μM IPTG LB media. Cells were harvested and concentrated 10 times in 25 mM Tris-HCl, 100 mM NaCl [pH 8.0]. Cells were lysed by sonication after addition of 1 mM PMSF, then stored at -80°C. Cleavage of trace amounts of 5′-^32^P-radiolabeled DNA (dpAA) or RNA (pAA) was tested in the presence of 25 mM Tris-HCl, 100 mM NaCl, 5 mM MgCl_2_ [pH 8.0]. Reactions were stopped by quenching in EDTA and urea, then analyzed by denaturing 20% PAGE.

### *Nocardioides alkalitolerans* growth and RT-qPCR

*Nocardioides alkalitolerans* strain (DSM no. 16699) was purchased from Leibniz Institute DSMZ-German Collection of Microorganisms and Cell Cultures and grown in Soy Broth media at 28°C. Liquid cultures (10 ml) were inoculated with a single colony and grown shaking at 28°C for 16 h to an OD_600_ of 0.6–0.8. For RNA extraction, 0.5 ml of each culture was added to 1 ml of RNAprotect Bacteria reagent (Qiagen), mixed and incubated for 5 min at 20°C. Bacterial cells were collected by centrifugation at 20°C at 5000 × g for 10 min and pellets were resuspended in 100 μl of 30 mM Tris, 1 mM EDTA [pH 8.0] supplemented with 15 mg/ml lysozyme and 10 mg/ml proteinase K. The suspensions were rotated at 20°C for 10 min, after which 700 μl buffer RLT (RNeasy Protect Bacteria mini kit, Qiagen) was added. Cells were lysed by bead beating in Bead Ruptor Elite (Omni International) at maximum speed at 8°C for 10 min using ceramic beads (0.1 mm, Revvity, Omni International). Beads and cell debris were pelleted by centrifugation at 20°C at 11 000 × g for 1 min. Supernatants were mixed with 590 μl of 80% ethanol and RNA purification proceeded on RNeasy spin columns (Qiagen) following the manufacturer's instructions. For reverse transcription, 50 ng of total RNA was treated with ezDNase enzyme (ThermoFisher) at 37°C for 10 min, followed by reverse transcription reaction in SuperScript IV VILO Master Mix (ThermoFisher). A SuperScript IV VILO No RT control (ThermoFisher) was included. Undiluted cDNA and No RT-controls were used in qPCR reactions. The following primers were used for the detection of *gyrA* (DNA gyrase subunit A) (33),*orn*, and *diDNase* genes: *diDNase* forward – TTGAACTGGCGGCAAAAC; *diDNase* reverse – TTAAGCACGGGACTGAGAC; *orn* forward – ACCTGATCGTCAAGCCC; *orn* reverse – ATGCGGTAGTGGAGGAAG; *gyrA* forward – CAGTACGTCTTCACGATCACC; *gyrA* reverse ATGAGCATGACCTCGTCACC. qPCR reactions were assembled in 4 technical replicates by combining 1 μl template (cDNA, No RT control or water), 0.5 μl of each primer (final 10 μM), 5 μl PowerTrack SYBR Green Master Mix (ThermoFisher) and 3.5 μl water. The thermocycles were run in standard cycling mode according to the PowerTrack SYBR Green Master Mix instructions in a CFX96 Real-Time PCR System (BioRad). The data were analyzed using CFX Maestro qPCR Analysis Software.

### Genomic environment analysis

Protein sequences of diDNases from Actinomycetes and Clostridia classes were identified using BLASTp searches against the NCBI non-redundant protein sequence database with default parameters (18). All identified protein sequences fulfilled the following criteria: length of approximately 200 amino acids, diDNase-characteristic EXOII sequence (consensus HHAxFD(L/I)xxF), and a C-terminal tyrosine residue. The search produced 103 unique sequences, of which 93 had RefSeq database entries that were used as input to the webFlaGs server (34). WebFlaGs analyses were performed with default parameters using 15 flanking genes. To identify mobilome genes, annotations of all 15 flanking genes were investigated, even if the genes were not part of conserved clusters predicted by the webFlaGs tool. To map prophage regions, the Phaster server (35) was used with default parameters. All detected prophage regions, including intact, questionable and incomplete regions, were considered. To locate known and predicted phage defense systems, the Padloc v2.0.0 software tool (36) was used with default settings. *DiDNase* genes were considered to colocalize if the detected defense system was within 10 genes from the diDNase-encoding locus.

## Results

### Distinctive NrnC homologs are present in Actinomycetes and Clostridia

**Figure 1.**
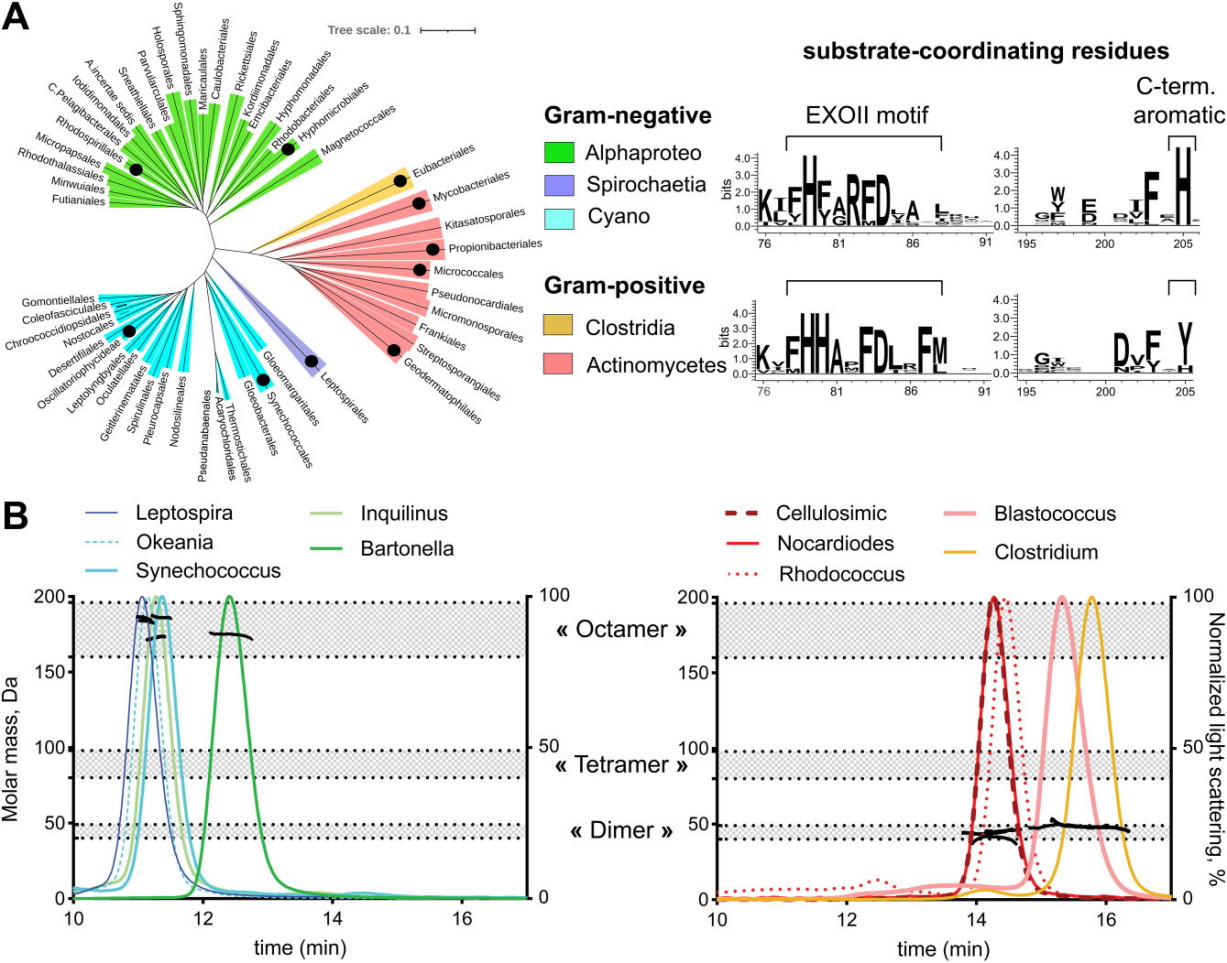
NrnC homologs from Gram-positive and Gram-negative bacteria differ in primary sequence and oligomeric state. **A**. Phylogenetic tree of NrnC homologs generated based on multiple sequence alignment (left). Taxonomic classes are indicated. Black dots mark orders from which representative homologs were further characterized: *Bartonella henselae* (Hyphonomonadales) (15), *Inquilinus* (Rhodospirillales), *Leptospira* (Leptospirales), *Synechococcus* (Synechococcales), *Okeania* (Ocsillatoriophycideae), *Clostridium* (Eubacteriales), *Nocardioides* (Mycobacteriales), *Cellulosimicrobium* (Micrococcales), *Blastococcus* (Geodermatophilales) and *Rhodococcus* (Propionibacteriales). Right: Sequence logos showing conservation at the EXOII motif and the C-terminal aromatic residue amongst NrnC homologs. Amino acid residue numbering is based on the sequence of *B. henselae* NrnC. **B**. Oligomeric state of NrnC homologs determined by SEC-MALS. The normalized light scattering signal (chromatogram lines, right axis) and molar mass values (dots across peaks, left axis) were plotted against the SEC elution volume. Shaded areas show the theoretical molar mass ranges for dimeric, tetrameric and octameric assemblies based on the primary sequence of the proteins.

NrnC homologs have been found in Alphaproteobacteria, Actinomycetes and Cyanobacteria (15), but only Alphaproteobacterial homologs have been studied in detail (9,15,17). Using an updated list of NrnC homologs, we constructed a phylogenetic tree based on the alignment of these protein sequences (Figure 1A). The tree shows a clear clustering of three distinct clades. One clade comprised NrnC homologs from Alphaproteobacteria, the second clade comprised those from Cyanobacteria and Spirochaetia, and the third covered Actinomycetal and Clostridial homologs (Figure 1A). Sequence alignments revealed several key features distinguishing NrnC homologs from the Gram-positive clade (Actinomycetes and Clostridia) and the two Gram-negative clades (Figure 1A and Supplementary Figure S1). First, in the regions important for substrate coordination, particularly the conserved EXOII motif and C-terminal aromatic residue, proteins from the Gram-negative group have a H(F/Y)(A/G)RFDxxx consensus sequence and a histidine residue, respectively. In contrast, in homologs from Gram-positive species, these amino acid positions are occupied by a HHAxFD(L/I)xF consensus sequence and a tyrosine residue (Figure 1A and Supplementary Figure S1A). Secondly, Alphaproteobacterial NrnCs from *Agrobacterium tumefaciens*, *Bartonella henselae*, and *Brucella melitensis* are known to form homo-octameric assemblies (15,17). Analysis of contacts between protomers based on the available crystal structures suggested that the residues responsible for octamerization are conserved in the Gram-negative group (Supplementary Figure S1). In contrast, these positions are variable in NrnC homologs from Gram-positive species (Supplementary Figure S1). Together, these sequence differences open the possibility of functional diversification between NrnC homologs from different species.

### NrnC homologs from Gram-positive bacteria function as diDNases *in vitro*

To investigate whether functional differences exist between NrnC homologs, we characterized representative proteins from each of the identified phylogenetic clades (Figure 1A). We recombinantly expressed and purified two NrnC homologs from Alphaproteobacteria, two from Cyanobacteria and one from Spirochaetia, as well as four NrnC-homologous proteins from Actinomycetes, and one from Clostridia. We first assessed the oligomeric state of the proteins by size-exclusion chromatography-coupled multi-angle light scattering (SEC-MALS). SEC-MALS data confirmed that proteins from Gram-negative species form octamers in solution, while proteins from Gram-positive orders appear as stable dimers, with no indication of higher-order assemblies (Figure 1B).

**Figure 2.**
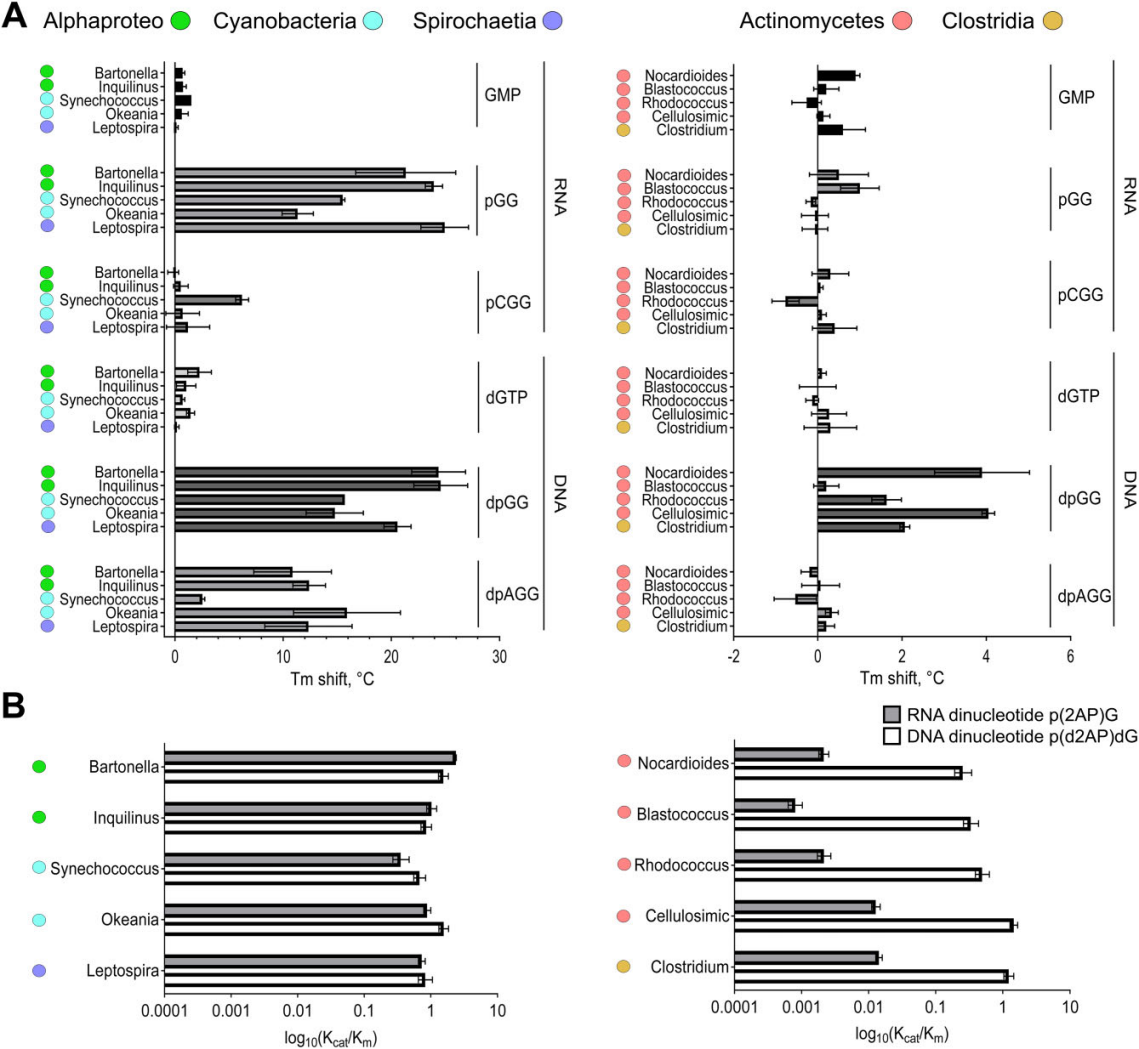
Substrate preferences of NrnC homologs from Gram-positive and Gram-negative bacteria. **A**. Graphs showing shifts in the melting temperature (T_m_) of different NrnC homologs in the presence of the indicated nucleotides. Bars show the mean value from triplicate experiments. Error bars show standard deviations. **B**. Catalytic efficiency of different NrnC homologs toward DNA and RNA dinucleotides, p(d2AP)dG and p(2AP)G, respectively. Values for k_cat_/K_m_ (× 10^6^ M^-1^ s^-1^) were plotted on a log_10_ scale. The error bars show 95% confidence interval of the model fitting.

Next, we examined by nDSF the substrate-binding profiles of NrnC homologs using ribonucleotides and deoxyribonucleotides one to three nucleotides in length as ligands (Figure 2A). Divalent cations were omitted from the buffers to suppress substrate cleavage. Proteins from the Gram-negative group showed the same substrate preference profiles as those previously established for *B. henselae* NrnC (15). Specifically, mono- and tri-ribonucleotides had little to no effect on the melting temperature of the proteins, whereas thermal stabilization by 11–28°C was observed with the 5′-phosphorylated diribonucleotide GG (pGG) (Figure 2A). Similar patterns were observed with deoxyribonucleotides, with the distinction that DNA trimers stabilized all the tested NrnCs, but to a lesser extent than deoxydinucleotide (deoxy-pGG or dpGG). In stark contrast, NrnC homologs from the Gram-positive group showed no signs of binding to any of the RNA ligands tested or to deoxynucleotides with one or three bases but experienced thermostability shifts in the presence of dpGG (Figure 2A).

Having observed that dimeric NrnC homologs from Gram-positive bacteria preferentially bind deoxydinucleotides, we next investigated whether these nucleotides are also their preferred substrates. For this, we measured the enzymatic activity of octameric and dimeric proteins and compared their catalytic efficiencies (k_cat_/K_m_) in cleaving 5′-phosphorylated DNA and RNA dinucleotides (Figure 2B and Supplementary Figure S2A–B, Supplementary Table S3). All assayed octameric NrnC proteins from Gram-negative species cleaved both RNA and DNA substrates with similar or equal efficiency (Figure 2B and Supplementary Figure S2B, Supplementary Table S3). In contrast, dimeric enzymes processed RNA dinucleotides 85–400 less efficiently, yet hydrolyzed DNA dinucleotides with an efficiency comparable to that of octameric NrnC homologs (Figure 2B and Supplementary Figure S2B, Supplementary Table S3). Even though the NrnC homolog from *Blastococcus* did not show a shift in thermal stability in the presence of dpGG (Figure 2A), it cleaved dpGG with high efficiency, suggesting that substrate binds to the enzyme without affecting its thermostability. Additionally, dimeric enzymes displayed no activity toward longer ssDNA nucleotides or dsDNA substrates (Supplementary Figures S2C and S3). In conclusion, the enzymatic activity of dimeric NrnC homologs from Gram-positive species defines these proteins as dedicated diDNases that are distinct from octameric NrnC homologs. To explore whether diDNases have preferences regarding the nucleic base composition of their substrates, we measured the activity of three combinations of purine and pyrimidine bases, using diDNase from *Nocardioides* (diDNase*_Noc_*) as a representative. The data showed that diDNase*_Noc_* had a slight preference for DNA dinucleotides containing pyrimidine at either the 5′ or 3′ position over the all-purine substrate (Supplementary Figure S2D, Supplementary Table S4).

### Conserved structural features define NrnC and diDNases as dinucleases

**Figure 3.**
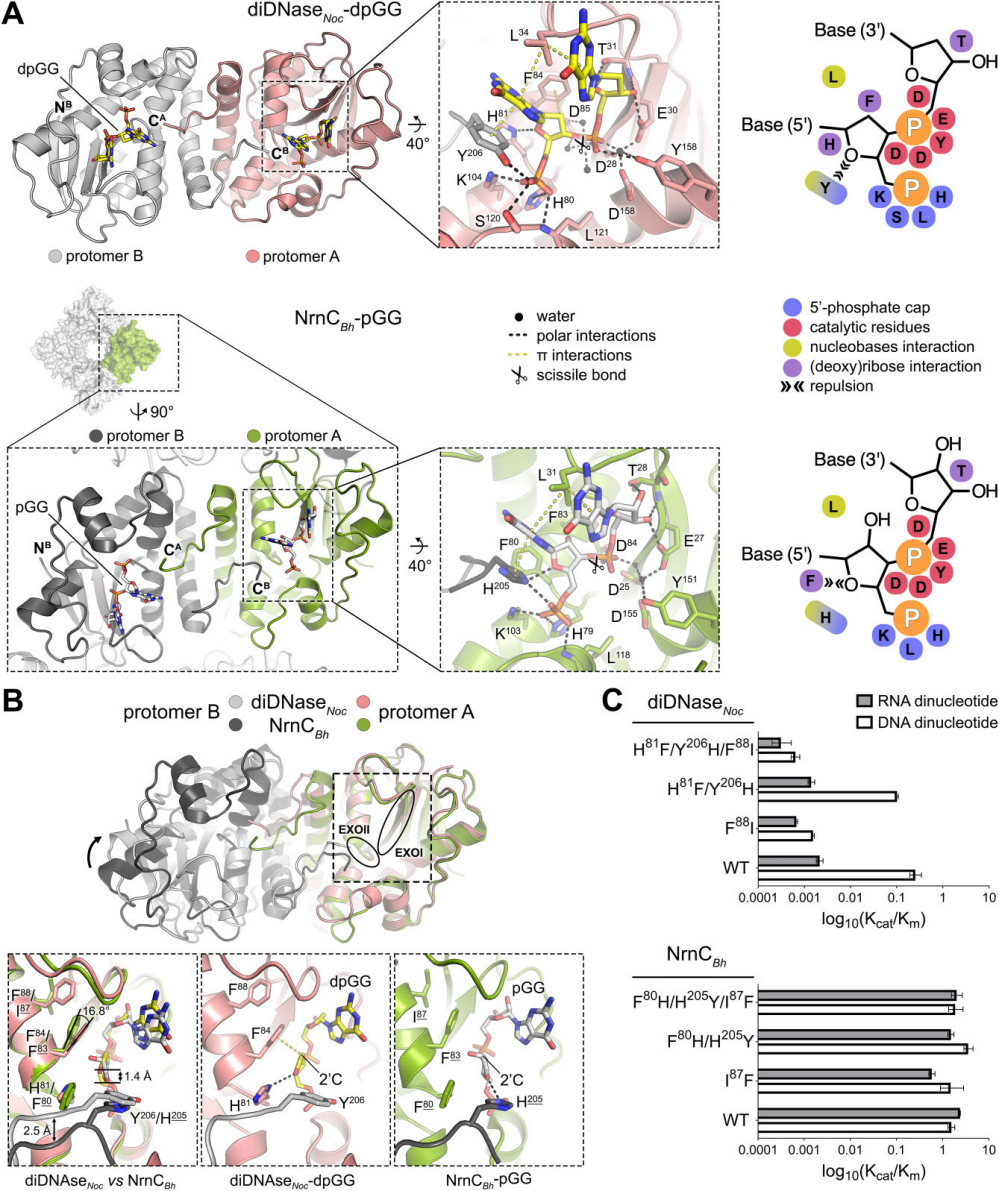
Structural basis for substrate specificity of diDNases. **A**. Structure of diDNase from *Nocardioides* (diDNase_Noc_) bound to dpGG. The top panel shows a dimeric assembly with a close-up of the substrate-bound active site. For comparison, the bottom panels show an octameric assembly of NrnC from *Bartonella henselae* (NrnC_Bh_) bound to pGG (PDB ID 7MPL), along with a dimeric subunit of the assembly and a close-up view of the active site. Schematic presentations of the substrate coordination in diDNases and NrnC homologs are shown (right panel). **B**. Structural alignments of diDNase_*Noc*_-dpGG and NrnC_*Bh*_-pGG using the EXOI and EXOII motifs (residues 28–31 and residues 78–87, respectively, per NrnC_Bh_ numbering) of one protomer as reference. The substrates were omitted for clarity. Close-ups highlight the nucleotides at the active sites and differences in the location and coordination of the 5′ sugars. The nucleobase at the 5′ residue was omitted from the two right panels for clarity. **C**. Catalytic efficiency of structure-based mutants of NrnC_*Bh*_ and diDNase_*Noc*_. Residues indicated in binding or restricting space for the 5′ pentose of substrates were swapped between NrnC and diDNase using mutagenesis. Enzyme kinetics of the mutant versions were measured and values for k_cat_/K_m_ (× 10^6^ M^-1^ s^-1^) were plotted on a log_10_ scale. The error bars show 95% confidence interval of the model fitting.

To understand the molecular basis for the specificity of dimeric NrnC homologs toward DNA substrates, we determined a set of crystal structures of diDNases: (i) a complex of diDNase*_Noc_* with dpGG to 1.55 Å (Figure 3A and Supplementary Figure S4A) and (ii) diDNase from *Rhodococcus* (diDNase*_Rho_*) in its apo state (1.72 Å resolution) and in complex with dpGG (2 Å resolution) (Supplementary Figure S4). Notably, the structure of the apo-form of diDNase*_Rho_* resulted from crystals set up in the presence of the RNA substrate pGG. However, no electron density was observed for pGG, confirming the low affinity of the protein for RNA substrates. The biologically relevant assembly in all crystal structures was determined to be a dimer using the ePISA server (37), which was consistent with the oligomeric state of the proteins in solution (Figure 1B). A comparison of structures of apo- and dpGG-bound diDNase*_Rho_* revealed no major conformational changes in the protein upon substrate binding (RMSD value of 0.45 Å for 1604 main-chain atoms of a diDNase dimer) (Supplementary Figure S4B). However, two minor differences were observed. First, region ^142^KTPRLS^147^ appeared disordered in the apo structure of diDNase*_Rho_*, but became visible and folded into a small helix when the substrate was present (Supplementary Figure S4B), owing to the direct interactions of the region with dpGG (Supplementary Figure S4C). The corresponding region in octameric NrnC was previously reported to be flexible in the apo state and its ordering was associated with substrate binding (15). Second, three side chains coordinating the substrate, H^88^, H^89,^ and Y^213^, adopt different rotamer conformations and turn toward the substrate in diDNase_*Rho*_-dpGG (Supplementary Figure S4B).

Superposition of diDNase*_Rho_*-dpGG and diDNase*_Noc_*-dpGG (RMSD of 1.32 Å for 813 main-chain atoms of a protomer) confirmed that the substrate-binding mode is almost identical between the diDNases (Supplementary Figure S4C). Small apparent differences pertain to hydrogen bonding at the 5′ end of the substrates and in contacts made by the aforementioned flexible region, ^142^KTPRLS^147^ in diDNase*_Rho_* or ^135^KGQQQS^140^ in diDNase*_Noc_*. In both structures, the 5′ phosphate of dpGG forms a set of salt bridges and hydrogen bonds with the side chains of conserved residues K^104^, H^80^ and Y^206^, the latter of which is contributed by the second protomer, and the main-chain nitrogen of L^121^ (per diDNase*_Noc_* residue numbering). Importance of the 5′ phosphate of the substrate for the enzyme-substrate interaction is evident from the absence of nDSF thermostabilization of diDNases when deoxydinucleotide without the 5′ phosphate (dGG) is used instead of dpGG (Supplementary Figure S4D). The side chain of Y^206^ also forms a π–π stacking interaction with the 5′ base of dpGG (Figure 3A). In diDNase*_Noc_*-dpGG, the side chain of the conserved residue Ser^120^ engages in another hydrogen bond with the 5′ phosphate, whereas in diDNase*_Rho_*-dpGG, it points away from the phosphate (Supplementary Figure S4C). The side chain of the invariant residue H^81^ forms a hydrogen bond with the ether oxygen of the deoxyribose cycle in both the structures (Figure 3A). An additional contact at the 5′ end of dpGG in both cases involves the partially hydrophobic cycle of the 5′ deoxyribose, which establishes a C–H–π interaction with the conserved F^84^ (Figure 3A). As expected, the phosphodiester bond is coordinated by the catalytic-site DEDDy signature residues, analogous to the coordination of RNA substrates by NrnC (Figure 3A and S4C) (15). Additionally, L^34^ splays the two bases of the substrate apart in the same manner as leucine wedges in dinucleases NrnC and Orn (Figure 3A) (14,15). The 3′ nucleotide is coordinated by the side chain of the conserved residue T^31^ and the main chain atoms spanning residues 30–34 as well as 135–140 (Figure 3A).

In summary, the overall position of the substrate and its interactions with diDNases are similar to those observed in NrnC-pGG complexes (Figure 3). The common key contacts include (1) restriction of the active site by the coordination of a 5′ phosphate by basic residues and a C-terminal residue of the second protomer, which is a histidine in NrnC and a tyrosine in diDNases; (2) π–π stacking of a C-terminal histidine or tyrosine residue with the 5′ nucleobase; (3) interactions of catalytic-site DEDDy residues with the scissile bond; (4) a leucine wedge; and (5) coordination of a 3′ nucleotide by a threonine residue and a loop region.

### Structural basis for preference of diDNases toward DNA substrates

To understand how diDNases discriminate between RNA and DNA dinucleotides, we took a closer look at the active sites of substrate-bound diDNase and octameric NrnC homologs. We confirmed that octameric NrnC coordinates both the DNA and RNA dinucleotides in the same way by determining the structure of *B. henselae* NrnC (NrnC*_Bh_*) bound to dpGG (NrnC*_Bh_*-dpGG) and comparing it with NrnC*_Bh_*-pGG (PDB ID 7MPL) (Supplementary Figure S4E). Superposition of the two structures revealed identical active site conformation and substrate engagement, regardless of whether NrnC*_Bh,_* was bound to a DNA or an RNA dinucleotide. This observation is consistent with kinetic data showing a lack of discrimination between the two substrates by NrnC*_Bh_*. The only minor structural difference pertains to the substrate dpGG, which sits slightly deeper in the active site than pGG does (Supplementary Figure S4E).

Next, we aligned the structures of the diDNase*_Noc_*-dpGG dimer with the corresponding dimeric unit of a NrnC*_Bh_*-pGG octamer using the EXOI and EXOII motifs of one of the protomers (protomer A) as reference points (Figure 3B). This allowed us to identify three elements that likely contribute to the inability of diDNases to accommodate RNA dinucleotides as substrates. First, superposition resulted in an obvious misalignment of the second protomer (protomer B) of the dimeric assemblies (Figure 3B). This result indicates that the details of the spatial arrangement of subunits within dimeric assemblies vary between diDNases and NrnC octamers despite the conservation of the general dimeric architecture. The inter-protomer difference correlates with structural changes in the active sites (Figure 3B). In the diDNase*_Noc_*-dpGG structure, the C-terminus of protomer B is pushed deeper into the active site of protomer A than that observed in the NrnC*_Bh_*-pGG structure, creating relatively less space for the 5′ end of the substrate.

The second prominent difference pertains to the interaction patterns of the enzymes with the 5′ sugar moieties of the substrates (Figure 3B). In NrnC*_Bh_*-pGG, the side chain of residue H^205^ forms a moderately strong hydrogen bond with a distance of 2.9 Å with the ether oxygen of the 5′ ribose, while in diDNase*_Noc_*-dpGG the side chain of Y^206^ repulses the ether oxygen of 5′ deoxyribose, pushing the entire nucleotide away. Coordination of the ether oxygen in diDNase*_Noc_*-dpGG is restored by the side chain of residue H^81^, which donates a hydrogen from the other side of the substrate. In NrnC*_Bh_*-pGG, the equivalent residue F^80^ repels the 5′ ribose. This position is part of the conserved EXOII motifs, and distinctions in EXOII between diDNases and NrnC homologs were already apparent in the primary sequence alignments (Figure 1 and Supplementary Figure S1). Together, the combination of a hydrogen bond involving a histidine residue and the repulsion created by a benzene ring of a phenylalanine or a tyrosine residue acting in opposing directions drives the 5′ pentose to adopt slightly different positions in the active sites of NrnC and diDNases. The position of the 5′ deoxyribose in diDNase*_Noc_*-dpGG allows for its C–H–π interaction with the conserved residue F^84^, whereas the 5′ ribose of pGG does not face the π-system of corresponding residue, F^83^, in NrnC*_Bh_*-pGG (Figure 3B).

The third discriminatory feature is the apparent restricted movement of the side chain of F^84^ in diDNase*_Noc_*, which engages in a perpendicular T-shaped stacking with the benzene ring of F^88^. F^88^ is conserved as part of the EXOII motif in dimeric diDNases, but not in octameric NrnC homologs, where the corresponding position is occupied by a residue with a volume smaller than phenylalanine (Figure 1 and Supplementary Figure S1). Specifically, in NrnC*_Bh_*-pGG, the side chain of F^83^ is buttressed by and flips 16.8° toward the side chain of I^87^ as compared to the position of Phe^84^ in diDNase*_Noc_*-dpGG (Figure 3B). Thus, the specific rotamer conformation of F^83^ in NrnC*_Bh_* creates more space to accommodate the 2′ hydroxyl group of the 5′ sugar of the RNA substrates. Such a configuration is prevented in diDNase*_Noc_* by the bulkier residue Phe^88^ in place of I^87^. Conservation of the involved residues indicates a systematic, diDNase-defining feature within the broader NrnC enzyme family.

Having identified three amino acid residues contributing to differential specificities in diDNase and NrnC, namely H^81^ or F^80^, Y^206^ or H^205^ and F^88^ or I^87^, we created mutants of diDNase*_Noc_* and NrnC*_Bh_* with those residues swapped in attempt to recreate strict diDNase substrate preference in an octameric NrnC scaffold or render a dimeric diDNase more promiscuous. All proteins harboring mutations could be purified and, according to SEC-MALS analysis, assembled into the same oligomeric state as their wild-type counterparts (Supplementary Figure S4F). Mutants of NrnC*_Bh_* with any of the residues exchanged to the corresponding residue from diDNases displayed activity levels toward both RNA and DNA dinucleotides similar to those of wild-type NrnC*_Bh_* (Figure 3C and Supplementary Figure S4G, Supplementary Table S5).

DiDNase*_Noc_* with H^81^F/Y^206^H mutations had activity analogous to that of wild-type diDNase*_Noc_*, including the high preference for DNA. However, mutating F^88^ to an isoleucine residue alone or in combination with H^81^F/Y^206^H in diDNase*_Noc_* resulted in significant loss of DNase activity without an increase in activity on diribonucleotides (Figure 3C and Supplementary Figure S4G, Supplementary Table S5). Together, this targeted mutational analysis indicated the importance of F^88^ for activity in diDNase, but also suggested more complex determinants of substrate specificity in DEDDy dinucleases.

The structural analysis argues that distinguishing between DNA and RNA in diDNases only occurs at the 5′ pentose of substrates, with the nature of the 3′ end not contributing to substrate specificity. To test this structure-based hypothesis, we measured the activity of diDNase*_Noc_* against an RNA-DNA hybrid dinucleotide with a ribonucleotide at the 5′ position as well as against a DNA-RNA hybrid with a ribonucleotide at the 3′ position. DiDNase*_Noc_* processed the RNA-DNA hybrid substrate 30–190 times less efficiently than all DNA dinucleotides, a catalytic efficiency almost as poor as for pure RNA substrates (Supplementary Figure S4H, Supplementary Table S4). In contrast, the DNA-RNA dinucleotide was cleaved with efficiency in the range of that for all DNA substrates (Supplementary Figure S4H, Supplementary Table S4). These results confirm that the 5′ position of the substrate is the main determinant of diDNase activity in this family of enzymes.

### DiDNases do not cleave RNA substrates *ex vivo*

**Figure 4.**
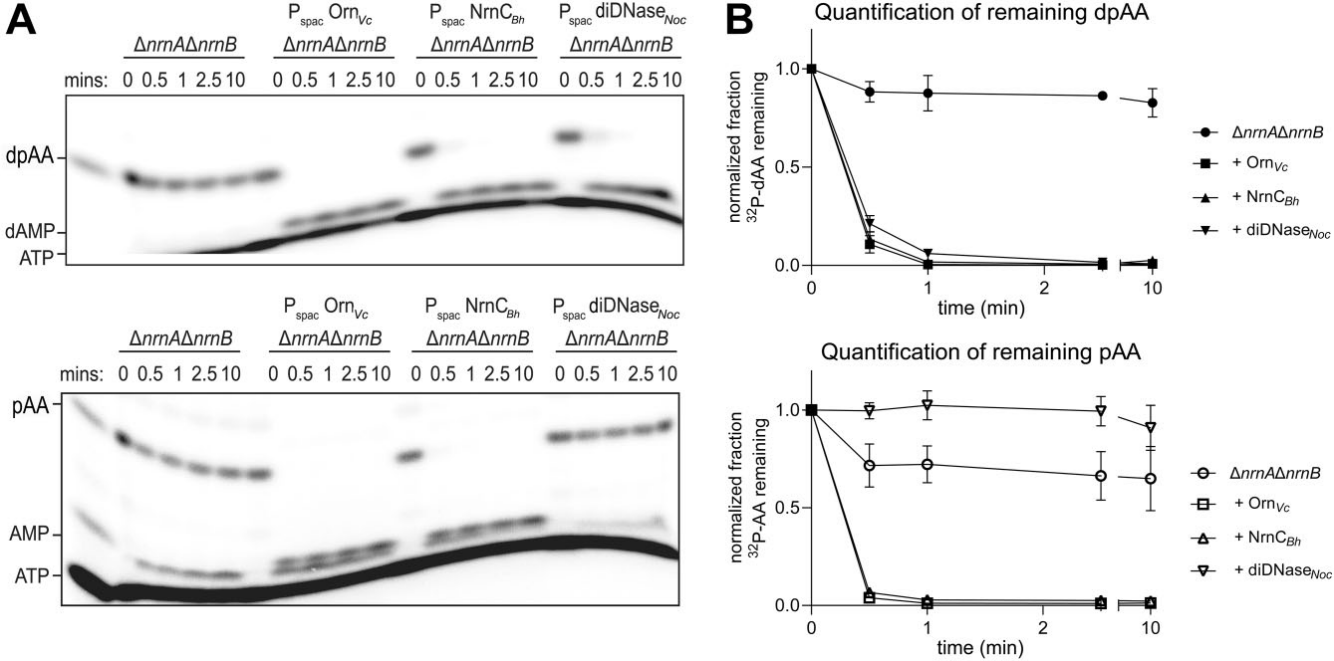
DNase and RNase activity of Orn and NrnC homologs in bacterial lysates. **A**. Denaturing 20% PAGE of control and experimental *B. subtilis ΔnrnAΔnrnB* lysates harboring an IPTG-inducible copy of Orn_*Vc*_, NrnC_*Bh*_ or diDNase_*Noc*_ in the non-essential amyE locus are shown. Reactions were stopped at the indicated intervals and hydrolysis of 5′-^32^P-radiolabeled DNA (dpAA) or RNA (pAA) substrates was evaluated. **B**. Graphs show normalized average radioactive intensity of substrate over time of reactions from three independent experiments. Error bars show standard deviations.

Thus far, enzyme function and substrate preference were investigated using purified proteins, leaving it open whether strict enzyme specificity is maintained in the complex environment of the cell. To address this, we compared the substrate preferences of representatives for Orn, NrnC, and diDNase (Orn*_Vc_*, NrnC*_Bh,_* and diDNase*_Noc_*) in *ex vivo* experiments using *Bacillus subtilis* lysates (Figure 4). Lysates of the *B. subtilis* strain lacking the two main nanoRNases A and B (*ΔnrnAΔnrnB* strain background) failed to support the degradation of deoxydinucleotide dpAA and were inefficient in breaking down the diribonucleotide pAA (38–40). The expression of either Orn*_Vc_* or NrnC*_Bh_* enabled the breakdown of both dinucleotide substrates. In contrast, lysates expressing diDNase*_Noc_* hydrolyzed only dpAA, but not pAA (Figure 4), consistent with the substrate preferences determined for the purified enzymes (Figure 2B, Supplementary Table S3). To what extent the activity of the non-discriminating dinucleases Orn*_Vc_* or NrnC*_Bh_* toward DNA dinucleotides contributes to their role in supporting cell growth is unknown. Having identified enzymes that specifically process DNA dinucleotides provided an opportunity to address this question.

### DiDNases cannot fully replace the housekeeping dinuclease function of Orn *in vivo*

**Figure 5.**
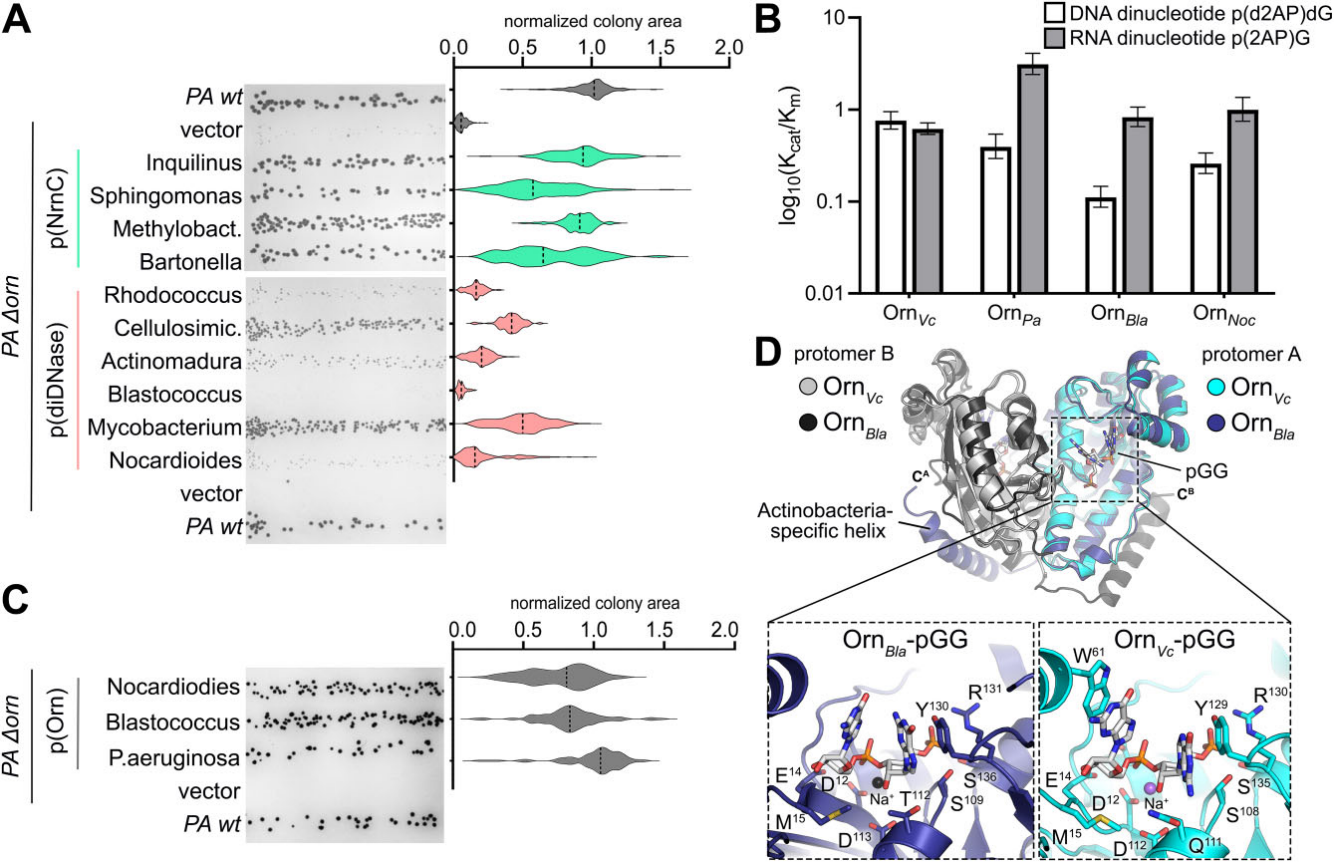
The cellular functions of Actinomycetal diDNases differ from those of Orn and NrnC. **A**. Complementation of the small-colony phenotype of *P. aeruginosa Δorn* using the indicated NrnC and diDNase homologs. Violin plots show colony sizes normalized to the average size of the wild-type *P. aeruginosa* colonies. Experiments were performed in triplicate. A minimum of 80 colonies per homolog were quantified. **B**. Catalytic efficiency k_cat_/K_m_ (× 10^6^ M^-1^ s^-1^) of different Orn homologs toward DNA and RNA dinucleotides. Error bars indicate 95% confidence interval of model fitting. **C**. Complementation of the small-colony phenotype of *P. aeruginosa Δorn* using the indicated Orn homologs. Experiments were performed as for panel A. **D**. Structural alignment of Orn_*Bla*_-pGG and Orn_*Vc*_-pGG (PDB ID 6N6A). Bottom panels highlight the sugar-phosphate backbone of pGG in the active site of the enzymes.

To untangle the effects of RNA and DNA dinucleotides on bacterial growth, we assessed whether diDNase activity was sufficient to compensate for the loss of a dinuclease capable of processing both RNA and DNA substrates. For this, we investigated whether diDNases could rescue the small-colony growth phenotype of the *Pseudomonas aeruginosa Δorn* strain. This strategy has been previously used to identify *Caulobacter crescentus* and *B. henselae* NrnC as functional analogs of Orn (9,12). As shown here, the expression of any octameric NrnC homolog tested rescued the *Δorn* phenotype, including representative enzymes from *Methylobacterium*, *Inquilinus*, and *Sphingomonas* species (Figure 5A and Supplementary Figure S5A).

The set of diDNases tested consisted of six proteins from Actinomycetes and did not include representatives from Clostridia owing to poor expression in *P. aeruginosa*. Quantification of the colony area of the *P. aeruginosa Δorn* strain expressing the different diDNases showed that their activities could only poorly or partially restore colony size, spanning a range of 0.05–0.5 of the level of wild-type *P. aeruginosa* depending on the homolog (Figure 5A). For comparison, the rescue efficiency achieved with octameric NrnC homologs ranged between 0.5 and 1 (Figure 5A). Although differences in complementation efficiency may be attributed to varying protein abundance of heterologously expressed homologs, no correlation between protein expression level and complementation efficiency was apparent (Supplementary Figure S5A). We also showed that overexpression of diDNase*_Noc_* has no apparent consequences on the growth of wild-type *P. aeruginosa* (Supplementary Figure S5B). In summary, it is likely that diDNases partially replace canonical dinucleases owing to their strong preference for DNA dinucleotides.

### DiDNase-containing organisms also encode a conserved Orn-family dinuclease

It has been reported that the genomes of Actinomycetes and Clostridia encode Orn and/or NrnA (13,15), which are likely performing a housekeeping function in these bacteria. To confirm that Actinomycetal Orn proteins function as dinucleases, we measured the *in vitro* and *in vivo* activities of Orn from *Blastococcus* (Orn*_Bla_*) and *Nocardioides* (Orn*_Noc_*) (Figure 5B–C, Supplementary Table S6). Actinomycetal Orn proteins readily cleaved both the RNA and DNA dinucleotides *in vitro* (Figure 5B and Supplementary Figure S5C, Supplementary Table S6). A comparison of their kinetic parameters with those of previously studied Orn homologs from Gammaproteobacteria *V. cholerae* and *P. aeruginosa*, Orn*_Vc_* and Orn*_Pa_*, respectively (11–14), revealed that Orn*_Vc_* did not distinguish between RNA and DNA dinucleotides and that Orn*_Pa_*, Orn*_Bla_, and* Orn*_Noc_* had a slight bias toward diribonucleotides (Figure 5B and Supplementary Figure S5C). Given that Orn*_Bla_* and Orn*_Noc_* were able to rescue the small-colony phenotype of the *P. aeruginosa Δorn* strain to the same extent as Orn*_Pa_* (Figure 5C and Supplementary Figure S5A), our results suggest a conserved cellular role for Orn proteins as housekeeping dinucleases.

The crystal structure of Orn*_Bla_* in complex with pGG further confirmed a conserved mode of substrate coordination across the bacterial Orn family, including homologs from Gram-positive species (Figure 5D and Supplementary Figure S5D). Structural superposition of Orn*_Bla_*-pGG and Orn*_Vc_*-pGG showed that the sugar-phosphate backbones of the substrates aligned perfectly. Neither structure revealed elements in the active sites that would restrict the space for the 2′OH group of the nucleotide's ribose. The residues of the active sites facing the substrate sugars are hydrophilic and can accommodate both deoxyribose and ribose (Figure 5D). The only significant structural difference between Orn*_Bla_* and Orn*_Vc_* is the additional C-terminal helix of the former, which is far from the active site and has been previously reported to stabilize the dimeric assembly of Orn from *M. smegmatis* (41).

In order to assess whether *diDNase* and *orn* genes are expressed in their Actinomycetal native host, we performed RT-qPCR with total RNA extracted from *Nocardioides alkalitolerans* grown in rich media to mid-logarithmic phase (Supplementary Figure S5E). Transcript levels for the *diDNase* gene appear comparable to those for DNA gyrase subunit A (*gyrA*), a house-keeping gene used here as a positive control. In contrast, *orn* appears to be expressed at a low level in this organism.

Taken together, our data indicate that NrnC homologs function as dedicated diDNases in Gram-positive species alongside housekeeping dinucleases, *i.e*. Orn proteins, that degrade RNA and DNA dinucleotides.

### Association of *diDNase* genes with MGEs and phage defense systems

**Figure 6.**
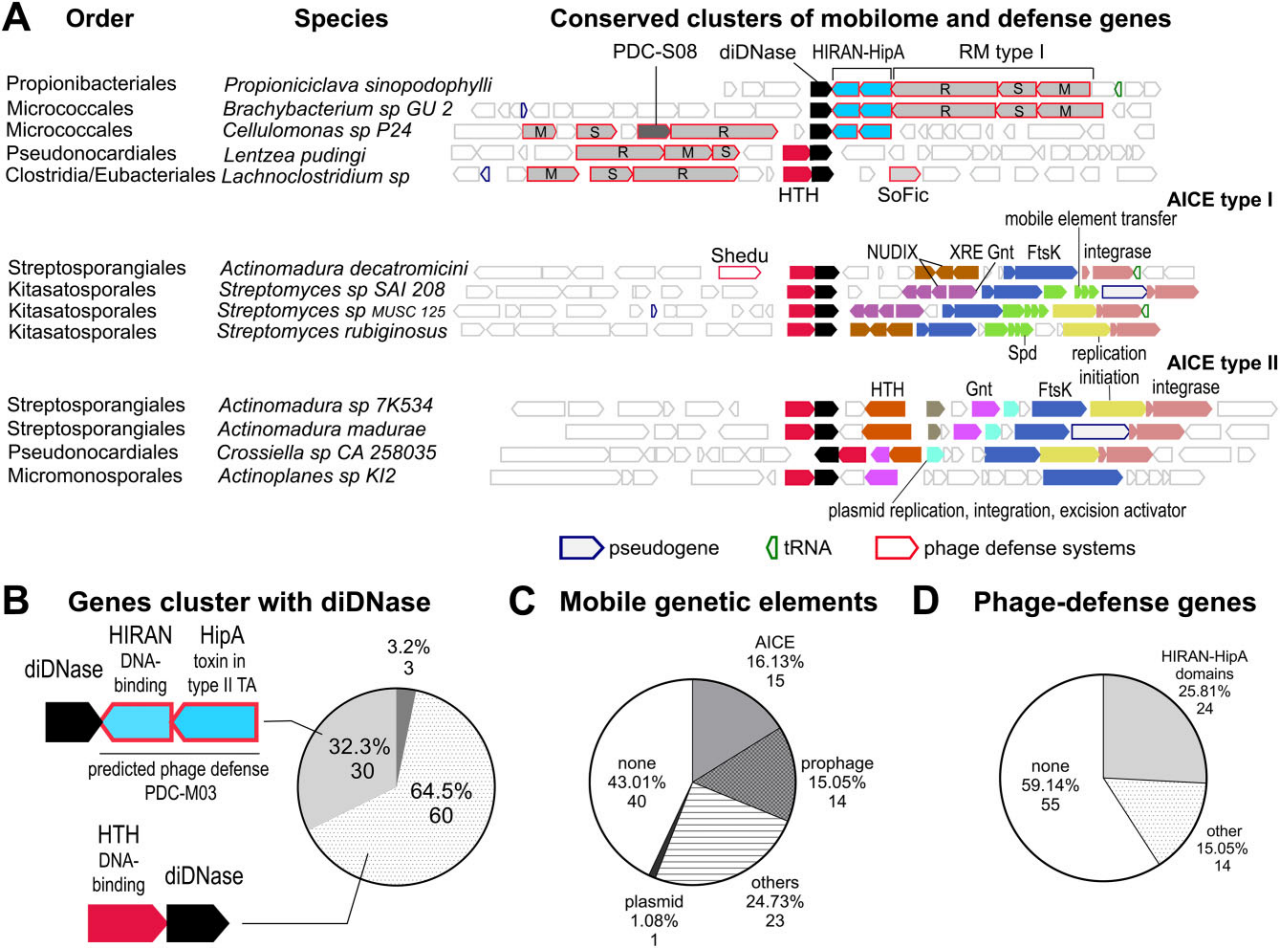
Genomic context of diDNase genes across species. **A**. Genomic environments of diDNases in representative species and orders. Phage defense systems are highlighted: restriction modification (RM) type I system (containing Restriction (R), modification (M) and specificity (S) modules), standalone protein with Fic domain (SoFic), SduA endonuclease (Shedu) and phage defense candidate (PDC)-S08. The following genes within Actinomycetal conjugative and integrative elements (AICE) are highlighted: transcriptional factor XRE, HTH, Gnt, conjugational transfer proteins Spd and FtsK, nucleoside diphosphates linked to x (NUDIX) hydrolase. **B**. Pie chart shows the frequency at which diDNase genes locate in gene clusters that include an operon with an HTH domain-containing protein, or a cluster with genes encoding HIRAN and HipA domain-containing proteins. **C**. Distribution of diDNase genes found near MGE genes, within prophage sequences, or inside AICEs. **D**. Statistics on the number of diDNase genes found near known and predicted phage defense systems.

Thus far, we have shown that the specific catalytic activity of diDNases is conserved across Actinomycetal and Clostridial homologs, suggesting selective pressure to preserve their function. At the same time, we noticed that the distribution of *diDNase* genes within the respective phylogenetic classes is patchy, which could point to horizontal gene transfer as a mode of *diDNase* gene propagation. To gain insight into their evolutionary trajectory and potential physiological role, we examined the genomic environment of *diDNase* genes using the webFlaGs tool (34). We analyzed all identified *diDNase* genes, amounting to 93 genes from different organisms (Supplemental Dataset 1; Supplementary Table S7); representative genomic arrangements are shown in Figure 6A. The query revealed that in 97% of the analyzed genomes, diDNases were encoded in one of two gene cluster architectures (Figure 6B). Of these, about two thirds were located directly next to a gene for a conserved helix-turn-helix (HTH) domain-containing protein (Figure 6B). The remaining third of *diDNase* genes were directly adjacent to genes encoding a HipA domain and a HIRAN domain protein (Figure 6B). HipA is a toxin in the HipAB type II toxin-antitoxin (TA) system and contains a serine/threonine protein kinase domain that phosphorylates different targets, leading to amino acid starvation and persistence (42–44). HIRAN domains are predicted to bind DNA and be involved in DNA damage response (45).

Suspecting horizontal gene transfer as a reason for the patchy distribution of diDNases across the tree of life, we investigated the genomic colocalization of *diDNase* genes with MGEs based on the gene annotations from the webFlaGs output. The analysis revealed that 57% of *diDNase* loci were surrounded by several classes of integrases, recombinases, transposases, and other proteins implicated in the life cycle of MGEs, also called mobilome genes (Figure 6C, Supplementary Table S7). In particular, we discovered that *diDNase* genes colocalized with genes typical of Actinomycete integrative and conjugative elements (AICEs) (16% of cases) (46). AICEs have highly conserved, modular organizations and contain tyrosine integrase, GntR, and XRE family transcriptional regulators for repression of AICE transfer; NUDIX hydrolase for prevention of redundant transfer; replication initiator protein RepSA; FtsK/SpoIIIE and Spd proteins for conjugative transfer (Figure 6A) (46). We identified two types of AICEs associated with *diDNase* genes in individual species, but their occurrence did not follow any apparent evolutionary lineage. The fact that these two AICEs types were present in some, but not every bacterial genome of a particular order suggests that horizontal gene transfer may have played a role in their phylogenetic distribution (Figure 6A). Furthermore, using the Phaster prophage mapping tool (35), the location of *diDNase* genes within prophage regions was observed in 15% of the analyzed genomes (Figure 6C). Another 23 *diDNase* genes (24.7%) were found to be within a 15 gene distance of a mobilome gene; however, the exact nature of the MGE could not be deduced (Figure 6C). One *diDNase* gene, that of *Nocardiopsis flavescens* NA01583, is encoded on a plasmid.

In total, we found that *diDNase* genes are part of one or another MGE or are located near a mobilome gene in the majority of genetic contexts (53 cases, 57%). This is in contrast to the genomic environments of the main housekeeping dinucleases in the same species, namely, *orn* in Actinomycetes (Supplemental Dataset 2, Supplementary Table S7) and *nrnA* in Clostridia (Supplemental Dataset 3, Supplementary Table S7). Both *nrnA* and most *orn* genes are located in regions of the core genome lacking mobilome genes in their vicinity.

Finally, we noticed components of the restriction-modification (RM) system type I near some *diDNase* genes in the webFlaGs output (Figure 6A). This result, together with the observation that most *diDNase* genes are associated with MGEs (47–50), prompted us to investigate whether *diDNase* genes are located near known phage defense genes. Phage defense genes tend to colocalize in so-called defense islands in bacterial genomes, and this property has been used extensively for the identification of new defense systems (47,51,52). Here, we used the Padloc tool (36) to query whether *diDNase* genes were mapped within 10 gene distances from known defense systems. In 41.9% of cases, *diDNase* genes were located near established defense genes (Figure 6D, Supplementary Table S7). Notably, the aforementioned HIRAN domain- and HipA domain-encoding genes forming a cluster with the *diDNase* genes identified in this study were predicted to have phage defense functions (36,53). As a control, we investigated the genomic association of *orn* and *nrnA* with anti-phage genes in the same set of 93 species. Using the same search distance, only two *orn* genes (2.1% frequency) were located proximal to RM system type I genes (Supplementary Table S7).

Other potentially beneficial genes were found near several *diDNase* genes, such as TA systems (10 cases, 11%) and multidrug resistance family MFS efflux pumps (15 cases, 16%), which may be useful for bacterial survival and competition (Supplementary Table S7). The close association of *diDNase* genes with MGEs and antiviral defense systems suggests a role for diDNase enzymes and deoxydinucleotide turnover in the associated processes.

## Discussion

Here, we characterized a novel enzyme group of the DEDDy family of nucleases that evolved to specifically cleave deoxydinucleotides. In analogy to the housekeeping dinucleases that are capable of hydrolyzing both RNA and DNA substrates, namely Orn and NrnC (14,15), we refer to the function of this newly defined NrnC-homologous family as ‘diDNase’.

Although diDNases share significant sequence and structural similarities with NrnCs, specific sequence motifs in diDNases result in structural changes that dictate their strict preference for DNA dinucleotides. In particular, H^81^, F^88^ and Y^206^ (per diDNase*_Noc_* numbering) were identified as diDNase-defining residues. The conservation of the substrate specificity-determining elements in diDNases suggests that the proteins were under selective pressure to discriminate between RNA and DNA substrates. However, it was also evident that targeted mutations at these specific sites were not sufficient to alter the substrate preference of a diDNase, suggesting a more complex interplay of structural motifs in the dimeric enzyme assemblies, including the exact positioning of the C-terminal tail in an adjacent subunit (Figure 3).

It is the lack of promiscuity in substrate preference that makes diDNases stand out from other di- or nano-nucleases, including structurally unrelated NrnA and NrnB (38,39). While canonical NrnC homologs are found in the core genomes of Alphaproteobacteria, Cyanobacteria and Spirochaetia classes, diDNases occur more sporadically in some but not all genomes of Actinomycetes and Clostridia. In addition to harboring these diDNase genes, Actinomycetes and Clostridia also encode housekeeping dinucleases Orn and/or NrnA. This observation suggests that there are scenarios or pathways, which require the deployment of additional diDNase activity that cannot be covered by the housekeeping dinucleases and that do not impact the diribonucleotide pool. Further support for a distinct cellular role of diDNases stems from the observation that these highly specific enzymes fail to fully replace enzymes with dinuclease activity, such as NrnA and NrnB in *B. subtilis* or Orn in *P. aeruginosa*, in cell lysates or cells, respectively (Figures 4 and 5). Therefore, it is possible that diDNases are important in processes other than clearing general dinucleotide pools, a function that is important for bacterial growth (15). At the same time, the residual potential of diDNases to counteract the loss of Orn in *P. aeruginosa* suggests that the growth defect of the *orn* deletion mutant is caused by the buildup of both RNA and DNA dinucleotides. This notion is supported by the observation that diDNase*_Noc_* only cleaved DNA-based dinucleotides in *B. subtilis ΔnrnAΔnrnB* lysates, whereas Orn and NrnC did not discriminate between RNA and DNA.

The first insight into the potential functions of diDNases arose from the analysis of their genomic context. This analysis showed that *diDNase* genes are found in diverse contexts with a high prevalence of hypothetical proteins and mobilome genes, which are hallmark features of genomic islands of probable horizontal origin (54–57). Notably, *diDNase* genes appear to be part of different types of MGEs, such as AICEs and prophages, and their association can vary even in closely related species, which may indicate a certain cost or lack of direct selective pressure to maintain specific diDNase-containing MGEs (Figure 6). Together, these observations point to the possibility that *diDNase* genes belong to an accessory genome and are mobile as cargo genes using different means for horizontal transfer between bacteria. This hypothesis is supported by the colocalization of some *diDNase* genes with other possibly beneficial cargo genes, such as TA systems and drug efflux pumps. In summary, the apparent association of diDNases with MGEs may provide an overall selective advantage to the recipient bacteria under certain conditions.

Despite the variability in MGE association, the vast majority of *diDNase* genes are directly adjacent to genes that encode one of two types of putative DNA-binding proteins containing either an HTH or a HIRAN domain. In the former case, the gene encoding the HTH domain-containing protein forms an operon with the *diDNase* gene (Figure 6). HTH domains are found in many transcriptional regulators, as well as in proteins implicated in DNA replication and repair, RNA metabolism, and protein–protein interactions in diverse signaling contexts (58). The HIRAN domain is predicted to recognize damaged DNA independently of its sequence and participates in rescuing stalled replication forks in eukaryotes (45,59). While the genetic association of diDNases with these factors suggests their involvement in chromosome-centric processes, the exact function of diDNase activity in these contexts remains to be established.

In the HIRAN domain-containing cluster, a third gene encodes a HipA domain-containing protein (Figure 6). HipA is a well-characterized toxin in *E. coli*, *C. crescentus* and *Shewanella oneidensis* (44,60,61). Several clades of HipA-related kinases and several types of putative antitoxins have been identified using phylogenetic analysis (62). HIRAN domains encoded in an operon with genes for HipA domain-containing proteins (designated as HipI) were predicted to act as antitoxins for HipA (62). A potential functional link between diDNases and HIRAN-HipA operons remains to be investigated.

In general, the antiviral defense arsenal of Actinobacteria is believed to be richer in rare systems than in other bacteria, and is predicted to be a source of novel anti-phage systems (63). In 42% of the genomes investigated, *diDNase* genes were located near a known or previously predicted antiviral system, in contrast to 2% of the corresponding housekeeping *orn* and *nrnA* genes. This association is above the 40% threshold used in a recent study, which discovered many novel defense systems using a ‘guilt-by-association’ approach (52), but lower than the stricter criteria used in earlier studies (47,51,64). However, the association of *diDNase* genes with defense islands may be underestimated because of the relatively high number of adjacent genes encoding hypothetical proteins, which may include yet to be discovered antiviral systems. The rapidly growing number of anti-phage genes being discovered and expanding knowledge about phage defense in bacteria in general (65) and Actinobacteria in particular (63) may make a more exhaustive assessment possible in the future. The frequent colocalization of *diDNase* genes with defense islands may indicate the role of their enzymatic activity in bacterial immunity and/or genotoxic stress. DNA nuclease activities are commonly found to be part of phage defense mechanisms, e.g. in restriction modification or CRISPR-Cas systems (66) and prokaryotic Argonautes (67), as well as the more recently discovered Gabija and Wadjet systems (68–70). These activities can target either phage nucleic acids to stop infection early on or host DNA as part of an abortive infection response. DiDNases may function in concert with other antiviral proteins to eliminate the accumulation of DNA fragments during these processes. The *diDNase* gene is expressed under normal growth conditions in *Nocardioides aklalitolerans* (Supplementary Figure S5E), where it is located inside a prophage. This finding may suggest a possible involvement of the diDNase in phage defense or superinfection resistance, considering that prophage-encoded defense systems have been shown to be expressed in lysogenic states of phages such as for prophages of Mycobacteria (71) and *P. aeruginosa* (72).

At the population level, diDNase function may confer an advantageous trait distributed in Actinomycetal and Clostridial pangenomes, being shared between organisms by horizontal gene transfer. At the molecular level, deoxydinucleotides may be produced by DNA damage and repair pathways, and their accumulation could lead to mis- or nano-priming, affect other enzymes in the organism, or lead to aberrant nucleotide signaling. Such effects would be analogous to the those of increased diribonucleotide levels on cellular physiology associated with the loss of housekeeping dinucleases, such as Orn, NrnC, and NrnA/NrnB (12,38,39,73,74). At the same time, the preservation of DNase activity in Orn, NrnA, NrnB, and NrnC, in addition to RNase activity, may indicate the importance of clearing DNA dinucleotides in cells. The discovery of highly specific diDNases provides a tool to untangle the effects of linear RNA- and DNA-based dinucleotides on cellular physiology.

## Supplementary Material

gkae1235_Supplemental_Files

## Data Availability

The atomic coordinates and structure factors have been deposited in the Protein Data Bank, www.rcsb.org, with the ID codes: 9F7G, 9F7J, 9F7D, 9F7L and 9F7M. Raw data for SEC-MALS, nDSF, enzymatic assays as well as uncropped blots and gels were deposited to the open repository Zenodo at the following citation: Mortensen, S. (2024). Structural and bioinformatics analyses identify deoxydinucleotide-specific nucleases [Data set]. Zenodo. https://doi.org/10.5281/zenodo.13981802.
