# Supplementary material for: Structural and bioinformatics analyses identify deoxydinucleotide-specific nucleases and their association with genomic islands in gram-positive bacteria
Source: Nucleic Acids Res. 2025 Jan 8;53(1):gkae1235. doi: 10.1093/nar/gkae1235 (PMC11706625; doi:10.1093/nar/gkae1235)
Supplement: gkae1235_Supplemental_Files [file gkae1235_supplemental_files.zip › diDNase_supplement_Datasets_NAR_R1.pdf]

Supplementary Datasets

Structural and bioinformatics analyses identify deoxydinucleotide-specific nucleases and their association with genomic islands in Gram-positive bacteria

Sofia Mortensen[1], Stanislava Kuncová[1], Justin D. Lormand[1], Tanner M. Myers[3], Soo-Kyoung Kim[4], Vincent T. Lee[4], Wade C. Winkler[3,4], and Holger Sondermann[1,2,*]

CONTENT

Supplementary Dataset 1. *Output of webFlaGs analysis of sets of diDNases.*

Supplementary Dataset 2. *Output of webFlaGs analysis of sets of orn genes from Actinomycete species that also encode diDNases.*

Supplementary Dataset 3. *Output of webFlaGs analysis of sets of nrnA genes from Clostridia species that encode diDNases.*

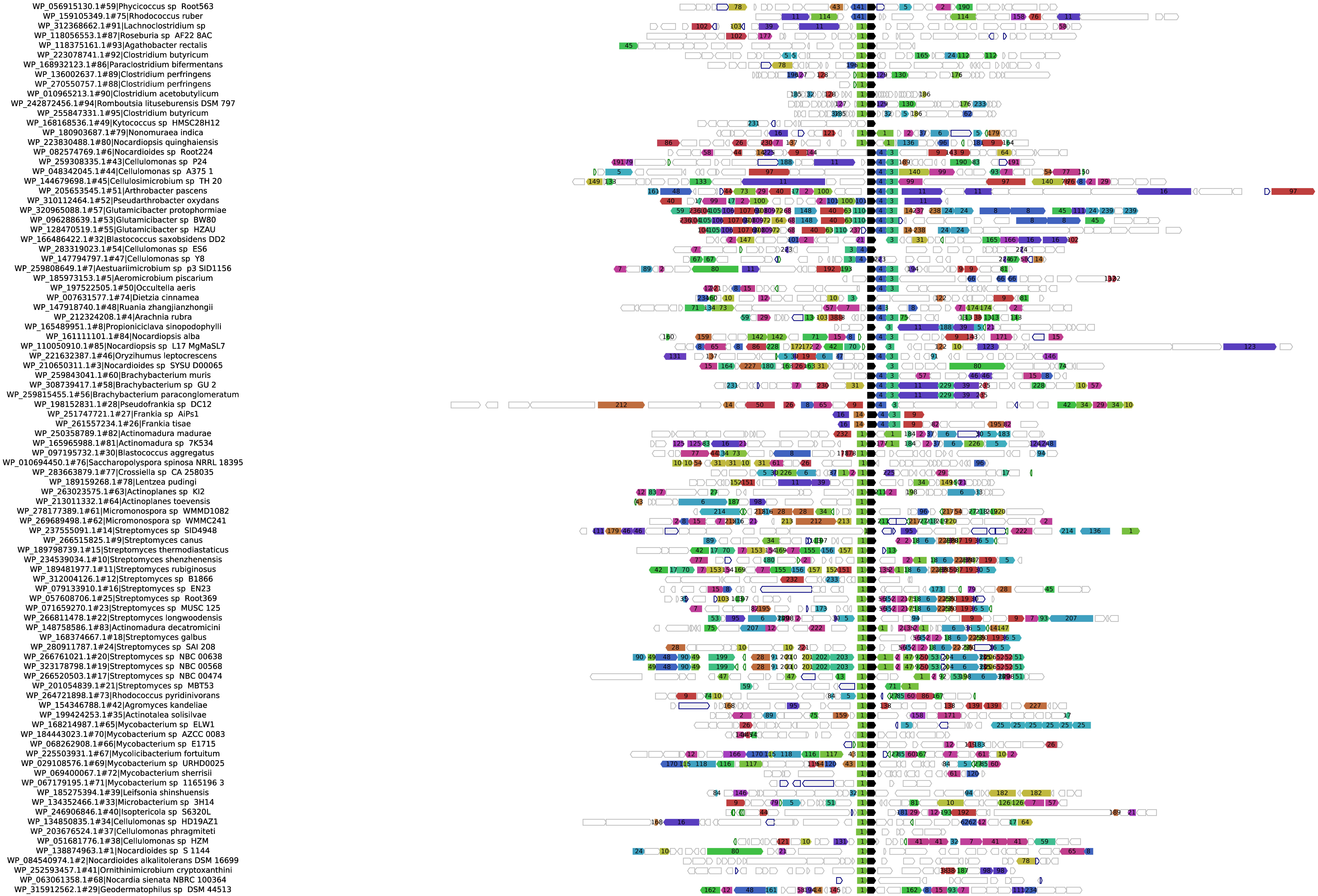
WP_056915130.1#59|Phycicoccus sp Root563
WP_159105349.1#75|Rhodococcus ruber
WP_312368662.1#91|Lachnoclostridium sp
WP_118056553.1#87|Roseburia sp AF22 8AC
WP_118375161.1#93|Agathobacter rectalis
WP_223078741.1#92|Clostridium butyricum
WP_168932123.1#86|Paraclostridium bifermentans
WP_136002637.1#89|Clostridium perfringens
WP_270550757.1#88|Clostridium perfringens
WP_010965213.1#90|Clostridium acetobutylicum
WP_242872456.1#94|Romboutsia lituseburensis DSM 797
WP_255847331.1#95|Clostridium butyricum
WP_168168536.1#49|Kytococcus sp HMSC28H12
WP_180903687.1#79|Nonomuraea indica
WP_223830488.1#80|Nocardiopsis quinghaiensis
WP_082574769.1#6|Nocardioides sp Root224
WP_259308335.1#43|Cellulomonas sp P24
WP_048342045.1#44|Cellulomonas sp A375 1
WP_144679698.1#45|Cellulosimicrobium sp TH 20
WP_205653545.1#51|Arthrobacter pascens
WP_310112464.1#52|Pseudarthrobacter oxydans
WP_320965088.1#57|Glutamicibacter protophormiae
WP_096288639.1#53|Glutamicibacter sp BW80
WP_128470519.1#55|Glutamicibacter sp HZAU
WP_166486422.1#32|Blastococcus saxobsidens DD2
WP_283319023.1#54|Cellulomonas sp ES6
WP_147794797.1#47|Cellulomonas sp Y8
WP_259808649.1#7|Aestuariimicrobium sp p3 SID1156
WP_185973153.1#5|Aeromicrobium piscarium
WP_197522505.1#50|Occultella aeris
WP_007631577.1#74|Dietzia cinnamea
WP_147918740.1#48|Ruania zhangjianzhongii
WP_212324208.1#4|Arachnia rubra
WP_165489951.1#8|Propioniciclava sinopodophylli
WP_161111101.1#84|Nocardiopsis alba
WP_110050910.1#85|Nocardiopsis sp L17 MgMaSL7
WP_221632387.1#46|Oryzihumus leptocrescens
WP_210650311.1#3|Nocardioides sp SYSU D00065
WP_259843041.1#60|Brachybacterium muris
WP_308739417.1#58|Brachybacterium sp GU 2
WP_259815455.1#56|Brachybacterium paraconglomeratum
WP_198152831.1#28|Pseudofrankia sp DC12
WP_251747721.1#27|Frankia sp AiPs1
WP_261557234.1#26|Frankia tisae
WP_250358789.1#82|Actinomadura madurae
WP_165965988.1#81|Actinomadura sp 7K534
WP_097195732.1#30|Blastococcus aggregatus
WP_010694450.1#76|Saccharopolyspora spinosa NRRL 18395
WP_283663879.1#77|Crossiella sp CA 258035
WP_189159268.1#78|Lentzea pudingi
WP_263023575.1#63|Actinoplanes sp KI2
WP_213011332.1#64|Actinoplanes toevensis
WP_278177389.1#61|Micromonospora sp WMMD1082
WP_269689498.1#62|Micromonospora sp WMMC241
WP_237555091.1#14|Streptomyces sp SID4948
WP_266515825.1#9|Streptomyces canus
WP_189798739.1#15|Streptomyces thermodiastaticus
WP_234539034.1#10|Streptomyces shenzhenensis
WP_189481977.1#11|Streptomyces rubiginosus
WP_312004126.1#12|Streptomyces sp B1866
WP_079133910.1#16|Streptomyces sp EN23
WP_057608706.1#25|Streptomyces sp Root369
WP_071659270.1#23|Streptomyces sp MUSC 125
WP_266811478.1#22|Streptomyces longwoodensis
WP_148758586.1#83|Actinomadura decatromicini
WP_168374667.1#18|Streptomyces galbus
WP_280911787.1#24|Streptomyces sp SAI 208
WP_266761021.1#20|Streptomyces sp NBC 00638
WP_323178798.1#19|Streptomyces sp NBC 00568
WP_266520503.1#17|Streptomyces sp NBC 00474
WP_201054839.1#21|Streptomyces sp MBT53
WP_264721898.1#73|Rhodococcus pyridinivorans
WP_154346788.1#42|Agromyces kandeliae
WP_199424253.1#35|Actinotalea solisilvae
WP_168214987.1#65|Mycobacterium sp ELW1
WP_184443223.1#70|Mycobacterium sp AZCC 0083
WP_068262908.1#66|Mycobacterium sp E1715
WP_225503931.1#67|Mycolicibacterium fortuitum
WP_029108576.1#69|Mycobacterium sp URHD0025
WP_069400067.1#72|Mycobacterium sherrisii
WP_067179195.1#71|Mycobacterium sp 1165196 3
WP_185275394.1#39|Leifsonia shinshuensis
WP_134352466.1#33|Microbacterium sp 3H14
WP_246906846.1#40|Isoptericola sp S6320L
WP_134850835.1#34|Cellulomonas sp HD19AZ1
WP_203676525.1#37|Cellulomonas phragmiteti
WP_051681776.1#38|Cellulomonas sp HZM
WP_138874963.1#1|Nocardioides sp S 1144
WP_084540974.1#2|Nocardioides alkalitolerans DSM 16699
WP_252593457.1#41|Ornithinimicrobium cryptoxanthini
WP_063061358.1#68|Nocardia sienata NBRC 100364
WP_315912562.1#29|Geodermatophilus sp DSM 44513

1(1)   WP_067179197.1 WP_067179197.1 hypothetical protein
1(1)   WP_057608705.1 WP_057608705.1 hypothetical protein
1(1)   WP_180903688.1 WP_180903688.1 hypothetical protein
1(1)   WP_199424254.1 WP_199424254.1 hypothetical protein
1(1)   WP_138874964.1 WP_138874964.1 hypothetical protein
1(1)   WP_068262903.1 WP_068262903.1 hypothetical protein
1(1)   WP_223078740.1 WP_223078740.1 hypothetical protein
1(1)   WP_106412447.1 WP_106412447.1 MULTISPECIES: XRE family transcriptional regulator
1(1)   WP_132045633.1 WP_132045633.1 hypothetical protein
1(1)   WP_266518742.1 WP_266518742.1 hypothetical protein
1(1)   WP_234538981.1 WP_234538981.1 hypothetical protein
1(1)   WP_039654759.1 WP_039654759.1 hypothetical protein
1(1)   WP_143539473.1 WP_143539473.1 hypothetical protein
1(1)   WP_266515830.1 WP_266515830.1 hypothetical protein
1(1)   WP_283663880.1 WP_283663880.1 hypothetical protein
1(1)   WP_118056554.1 WP_118056554.1 MULTISPECIES: hypothetical protein
1(1)   WP_185275395.1 WP_185275395.1 hypothetical protein
1(1)   WP_213011331.1 WP_213011331.1 hypothetical protein
1(1)   WP_234539032.1 WP_234539032.1 XRE family transcriptional regulator
1(1)   WP_148758585.1 WP_148758585.1 hypothetical protein
1(2)   WP_266761023.1 WP_266761023.1 MULTISPECIES: XRE family transcriptional regulator
1(1)   WP_283663882.1 WP_283663882.1 DUF5919 domain-containing protein
1(1)   WP_203676526.1 WP_203676526.1 hypothetical protein
1(1)   WP_278177388.1 WP_278177388.1 hypothetical protein
1(2)   WP_136002636.1 WP_136002636.1 hypothetical protein
1(1)   WP_243718307.1 WP_243718307.1 transcriptional regulator
1(1)   WP_252593460.1 WP_252593460.1 MULTISPECIES: DUF2157 domain-containing protein
1(1)   WP_161302355.1 WP_161302355.1 MULTISPECIES: hypothetical protein
1(1)   WP_149382910.1 WP_149382910.1 hypothetical protein
1(1)   WP_150241395.1 WP_150241395.1 XRE family transcriptional regulator
1(2)   WP_266761019.1 WP_266761019.1 MULTISPECIES: hypothetical protein
1(1)   WP_189159269.1 WP_189159269.1 hypothetical protein

1(1)	WP_141743892.1	WP_141743892.1 DUF1499 domain-containing protein
1(1)	WP_069400068.1	WP_069400068.1 hypothetical protein
1(1)	WP_148758587.1	WP_148758587.1 XRE family transcriptional regulator
1(1)	WP_189798738.1	WP_189798738.1 hypothetical protein
1(1)	WP_148758591.1	WP_148758591.1 helix-turn-helix transcriptional regulator
1(1)	WP_280911786.1	WP_280911786.1 hypothetical protein
1(1)	WP_241393077.1	WP_241393077.1 MULTISPECIES: hypothetical protein
1(1)	WP_029289512.1	WP_029289512.1 hypothetical protein
1(1)	WP_269689499.1	WP_269689499.1 hypothetical protein
1(1)	WP_255847330.1	WP_255847330.1 hypothetical protein
1(1)	WP_157571558.1	WP_157571558.1 hypothetical protein
1(1)	WP_189481978.1	WP_189481978.1 hypothetical protein
1(1)	WP_246906743.1	WP_246906743.1 hypothetical protein
1(1)	WP_236584744.1	WP_236584744.1 transcriptional regulator, partial
1(1)	WP_092727730.1	WP_092727730.1 hypothetical protein
1(1)	WP_166527625.1	WP_166527625.1 hypothetical protein
1(1)	WP_134352465.1	WP_134352465.1 hypothetical protein
1(1)	WP_118375160.1	WP_118375160.1 hypothetical protein
1(1)	WP_157106164.1	WP_157106164.1 hypothetical protein
1(1)	WP_168932126.1	WP_168932126.1 hypothetical protein
1(1)	WP_264721897.1	WP_264721897.1 hypothetical protein
1(1)	WP_250358787.1	WP_250358787.1 helix-turn-helix transcriptional regulator
1(1)	WP_312004125.1	WP_312004125.1 hypothetical protein
1(1)	WP_168374666.1	WP_168374666.1 hypothetical protein
1(1)	WP_184443024.1	WP_184443024.1 hypothetical protein
1(1)	WP_201054840.1	WP_201054840.1 hypothetical protein
1(1)	WP_250358790.1	WP_250358790.1 hypothetical protein
1(1)	WP_266518746.1	WP_266518746.1 XRE family transcriptional regulator
1(1)	WP_202451611.1	WP_202451611.1 MFS transporter
1(1)	WP_263023574.1	WP_263023574.1 hypothetical protein
1(1)	WP_097195733.1	WP_097195733.1 hypothetical protein
1(1)	WP_029108575.1	WP_029108575.1 hypothetical protein
1(1)	WP_154346786.1	WP_154346786.1 hypothetical protein
1(1)	WP_266811479.1	WP_266811479.1 hypothetical protein
1(1)	WP_225503930.1	WP_225503930.1 hypothetical protein

1(1)   WP_134850834.1 WP_134850834.1 hypothetical protein
1(1)   WP_101788517.1 WP_101788517.1 hypothetical protein
1(1)   WP_312368660.1 WP_312368660.1 hypothetical protein
1(1)   WP_150241402.1 WP_150241402.1 hypothetical protein

2(1)   WP_280911791.1 WP_280911791.1 GntR family transcriptional regulator
2(1)   WP_280913370.1 WP_280913370.1 NUDIX hydrolase
2(1)   WP_269689512.1 WP_269689512.1 NUDIX hydrolase
2(2)   WP_266761024.1 WP_266761024.1 MULTISPECIES: GntR family transcriptional regulator
2(1)   WP_148758590.1 WP_148758590.1 NUDIX domain-containing protein
2(1)   WP_014373978.1 WP_014373978.1 pyridoxal phosphate-dependent aminotransferase
2(1)   WP_263023576.1 WP_263023576.1 GntR family transcriptional regulator
2(1)   WP_266515822.1 WP_266515822.1 NUDIX domain-containing protein
2(1)   WP_039654755.1 WP_039654755.1 MULTISPECIES: GntR family transcriptional regulator
2(1)   WP_057608708.1 WP_057608708.1 NUDIX hydrolase
2(1)   WP_199424264.1 WP_199424264.1 PLP-dependent aminotransferase family protein
2(1)   WP_269689490.1 WP_269689490.1 NAD(+) diphosphatase
2(1)   WP_266518747.1 WP_266518747.1 winged helix-turn-helix domain-containing protein
2(1)   WP_310112456.1 WP_310112456.1 FCD domain-containing protein
2(1)   WP_234538987.1 WP_234538987.1 GntR family transcriptional regulator
2(1)   WP_101788515.1 WP_101788515.1 GntR family transcriptional regulator
2(1)   WP_168374680.1 WP_168374680.1 NUDIX domain-containing protein
2(1)   WP_189481975.1 WP_189481975.1 NUDIX domain-containing protein
2(1)   WP_039654756.1 WP_039654756.1 MULTISPECIES: NUDIX hydrolase
2(1)   WP_051027509.1 WP_051027509.1 MULTISPECIES: FadR/GntR family transcriptional regulator

2(1)   WP_234538979.1 WP_234538979.1 NUDIX domain-containing protein
2(1)   WP_266515819.1 WP_266515819.1 GntR family transcriptional regulator
2(1)   WP_310112452.1 WP_310112452.1 histidinol-phosphate transaminase
2(1)   WP_205653549.1 WP_205653549.1 histidinol-phosphate transaminase
2(1)   WP_259808638.1 WP_259808638.1 (deoxy)nucleoside triphosphate pyrophosphohydrolase
2(1)   WP_266811488.1 WP_266811488.1 NUDIX hydrolase
2(1)   WP_250362731.1 WP_250362731.1 GntR family transcriptional regulator
2(1)   WP_132045622.1 WP_132045622.1 GntR family transcriptional regulator
2(1)   WP_064896534.1 WP_064896534.1 GntR family transcriptional regulator
2(1)   WP_110050908.1 WP_110050908.1 GntR family transcriptional regulator
2(1)   WP_082552842.1 WP_082552842.1 MULTISPECIES: pyridoxal phosphate-dependent aminotransferase
2(1)   WP_057608709.1 WP_057608709.1 GntR family transcriptional regulator
2(1)   WP_014373984.1 WP_014373984.1 GntR family transcriptional regulator
2(1)   WP_147918752.1 WP_147918752.1 NUDIX domain-containing protein
2(1)   WP_148758588.1 WP_148758588.1 GntR family transcriptional regulator
2(1)   WP_283663881.1 WP_283663881.1 NUDIX domain-containing protein
2(1)   WP_168374671.1 WP_168374671.1 GntR family transcriptional regulator

3(1)   WP_096288641.1 WP_096288641.1 hypothetical protein
3(1)   WP_259843039.1 WP_259843039.1 HipA domain-containing protein
3(1)   WP_146223282.1 WP_146223282.1 hypothetical protein
3(1)   WP_147794799.1 WP_147794799.1 hypothetical protein
3(1)   WP_144679702.1 WP_144679702.1 HipA domain-containing protein

3(1)	WP_156391105.1	WP_156391105.1 MULTISPECIES: hypothetical protein
3(1)	WP_283319021.1	WP_283319021.1 hypothetical protein
3(1)	WP_251747723.1	WP_251747723.1 hypothetical protein
3(1)	WP_308739415.1	WP_308739415.1 hypothetical protein
3(1)	WP_246861932.1	WP_246861932.1 hypothetical protein
3(1)	WP_161111103.1	WP_161111103.1 hypothetical protein
3(1)	WP_212324204.1	WP_212324204.1 hypothetical protein
3(1)	WP_166486421.1	WP_166486421.1 HipA domain-containing protein
3(1)	WP_053071549.1	WP_053071549.1 hypothetical protein
3(1)	WP_128470517.1	WP_128470517.1 hypothetical protein
3(1)	WP_261557231.1	WP_261557231.1 hypothetical protein
3(1)	WP_131885644.1	WP_131885644.1 hypothetical protein
3(1)	WP_131166523.1	WP_131166523.1 hypothetical protein
3(1)	WP_168211886.1	WP_168211886.1 hypothetical protein
3(1)	WP_259815458.1	WP_259815458.1 hypothetical protein
3(1)	WP_205653543.1	WP_205653543.1 hypothetical protein
3(1)	WP_156741163.1	WP_156741163.1 hypothetical protein
3(1)	WP_143914083.1	WP_143914083.1 hypothetical protein
3(1)	WP_310112467.1	WP_310112467.1 hypothetical protein
3(1)	WP_259308337.1	WP_259308337.1 hypothetical protein
3(1)	WP_045876245.1	WP_045876245.1 hypothetical protein
3(1)	WP_320965090.1	WP_320965090.1 HipA domain-containing protein
3(1)	WP_141786876.1	WP_141786876.1 hypothetical protein
3(1)	WP_259808651.1	WP_259808651.1 hypothetical protein

4(1)	WP_144679700.1	WP_144679700.1 HIRAN domain-containing protein
4(1)	WP_205653544.1	WP_205653544.1 hypothetical protein
4(1)	WP_128470518.1	WP_128470518.1 HIRAN domain-containing protein
4(1)	WP_259308336.1	WP_259308336.1 hypothetical protein
4(1)	WP_143914082.1	WP_143914082.1 hypothetical protein
4(1)	WP_232303677.1	WP_232303677.1 hypothetical protein
4(1)	WP_147794798.1	WP_147794798.1 hypothetical protein
4(1)	WP_259808650.1	WP_259808650.1 hypothetical protein
4(1)	WP_308739416.1	WP_308739416.1 hypothetical protein
4(1)	WP_259815456.1	WP_259815456.1 hypothetical protein
4(1)	WP_141393599.1	WP_141393599.1 hypothetical protein

4(1)   WP_161111102.1 WP_161111102.1 hypothetical protein
4(1)   WP_212324206.1 WP_212324206.1 hypothetical protein
4(1)   WP_310112465.1 WP_310112465.1 hypothetical protein
4(1)   WP_251747722.1 WP_251747722.1 hypothetical protein
4(1)   WP_283319022.1 WP_283319022.1 hypothetical protein
4(1)   WP_259843040.1 WP_259843040.1 hypothetical protein
4(1)   WP_048342046.1 WP_048342046.1 HIRAN domain-containing protein
4(1)   WP_147918741.1 WP_147918741.1 hypothetical protein
4(1)   WP_141786875.1 WP_141786875.1 hypothetical protein
4(1)   WP_261557232.1 WP_261557232.1 hypothetical protein
4(1)   WP_156741164.1 WP_156741164.1 hypothetical protein
4(1)   WP_156391106.1 WP_156391106.1 MULTISPECIES: hypothetical protein
4(1)   WP_320965089.1 WP_320965089.1 hypothetical protein

5(1)   WP_316301477.1 WP_316301477.1 site-specific integrase

5(1)   WP_189481959.1 WP_189481959.1 site-specific integrase

5(1)   WP_280911797.1 WP_280911797.1 site-specific integrase

5(1)   WP_246178598.1 WP_246178598.1 site-specific integrase

5(1)   WP_235529051.1 WP_235529051.1 MULTISPECIES: tyrosine recombinase XerC
5(1)   WP_134352458.1 WP_134352458.1 site-specific integrase

5(1)   WP_197080755.1 WP_197080755.1 tyrosine-type recombinase/integrase
5(1)   WP_168374678.1 WP_168374678.1 site-specific integrase

5(1)   WP_250358780.1 WP_250358780.1 site-specific integrase

5(1)   WP_029108587.1 WP_029108587.1 site-specific integrase

5(1)   WP_255847336.1 WP_255847336.1 tyrosine-type recombinase/integrase
5(1)   WP_234787735.1 WP_234787735.1 MULTISPECIES: site-specific integrase
5(1)   WP_131166526.1 WP_131166526.1 tyrosine-type

recombinase/integrase
5(1) WP_266515792.1 WP_266515792.1 site-specific integrase

5(1) WP_281260300.1 WP_281260300.1 site-specific integrase

5(1) WP_141786868.1 WP_141786868.1 site-specific integrase

5(1) WP_039654747.1 WP_039654747.1 site-specific integrase

5(1) WP_316301479.1 WP_316301479.1 Arm DNA-binding domain-containing protein
5(1) WP_266811480.1 WP_266811480.1 site-specific integrase

5(1) WP_264721896.1 WP_264721896.1 site-specific integrase

5(1) WP_132045616.1 WP_132045616.1 tyrosine-type recombinase/integrase
5(1) WP_283663889.1 WP_283663889.1 tyrosine-type recombinase/integrase
5(1) WP_234538964.1 WP_234538964.1 site-specific integrase

6(1) WP_250358782.1 WP_250358782.1 FtsK/SpoIIIE domain-containing protein
6(1) WP_283663887.1 WP_283663887.1 FtsK/SpoIIIE domain-containing protein
6(1) WP_266811500.1 WP_266811500.1 cell division protein FtsK
6(1) WP_266518751.1 WP_266518751.1 cell division protein FtsK
6(1) WP_234538977.1 WP_234538977.1 FtsK/SpoIIIE domain-containing protein
6(1) WP_039654754.1 WP_039654754.1 MULTISPECIES: FtsK/SpoIIIE domain-containing protein
6(1) WP_266515814.1 WP_266515814.1 FtsK/SpoIIIE domain-containing protein
6(1) WP_243718306.1 WP_243718306.1 FtsK/SpoIIIE domain-containing protein
6(1) WP_057608711.1 WP_057608711.1 FtsK/SpoIIIE domain-containing protein
6(1) WP_168374673.1 WP_168374673.1 FtsK/SpoIIIE

domain-containing protein
6(1)   WP_101788511.1 WP_101788511.1 FtsK/SpoIIIE domain-containing protein
6(1)   WP_213011323.1 WP_213011323.1 FtsK/SpoIIIE domain-containing protein
6(1)   WP_189481972.1 WP_189481972.1 FtsK/SpoIIIE domain-containing protein
6(1)   WP_280911792.1 WP_280911792.1 FtsK/SpoIIIE domain-containing protein
6(1)   WP_221632385.1 WP_221632385.1 FtsK/SpoIIIE domain-containing protein
6(1)   WP_263023690.1 WP_263023690.1 cell division protein FtsK
6(2)   WP_266761034.1 WP_266761034.1 MULTISPECIES: cell division protein FtsK
6(1)   WP_148758594.1 WP_148758594.1 FtsK/SpoIIIE domain-containing protein

7(1)   WP_228731537.1 WP_228731537.1 ABC transporter ATP-binding protein
7(1)   WP_166527617.1 WP_166527617.1 ABC transporter ATP-binding protein
7(1)   WP_259808634.1 WP_259808634.1 ABC transporter ATP-binding protein
7(1)   WP_039654766.1 WP_039654766.1 MULTISPECIES: ATP-binding cassette domain-containing protein
7(1)   WP_150241412.1 WP_150241412.1 ABC transporter ATP-binding protein
7(1)   WP_225503933.1 WP_225503933.1 ATP-binding cassette domain-containing protein
7(1)   WP_263023562.1 WP_263023562.1 ABC transporter ATP-binding protein
7(1)   WP_269689508.1 WP_269689508.1 ABC transporter ATP-binding protein
7(1)   WP_308739422.1 WP_308739422.1 ATP-binding cassette domain-containing protein
7(1)   WP_134352475.1 WP_134352475.1 sugar ABC transporter ATP-binding protein
7(1)   WP_189798730.1 WP_189798730.1 ATP-binding cassette domain-containing protein
7(1)   WP_048342050.1 WP_048342050.1 ATP-binding cassette

domain-containing protein
7(1)   WP_283319010.1 WP_283319010.1 ABC transporter ATP-binding protein
7(1)   WP_168218095.1 WP_168218095.1 MULTISPECIES: ATP-binding cassette domain-containing protein
7(1)   WP_189798734.1 WP_189798734.1 ABC transporter ATP-binding protein
7(1)   WP_147918748.1 WP_147918748.1 phosphate ABC transporter ATP-binding protein PstB
7(1)   WP_210418138.1 WP_210418138.1 ABC-F family ATP-binding cassette domain-containing protein
7(1)   WP_029289495.1 WP_029289495.1 energy-dependent translational throttle protein EttA
7(1)   WP_006136585.1 WP_006136585.1 MULTISPECIES: ATP-binding cassette domain-containing protein

8(1)   WP_147918744.1 WP_147918744.1 response regulator transcription factor
8(1)   WP_320965097.1 WP_320965097.1 LuxR C-terminal-related transcriptional regulator
8(1)   WP_166527620.1 WP_166527620.1 response regulator transcription factor
8(1)   WP_110050898.1 WP_110050898.1 response regulator transcription factor
8(1)   WP_218108608.1 WP_218108608.1 response regulator transcription factor
8(1)   WP_017545771.1 WP_017545771.1 MULTISPECIES: response regulator transcription factor
8(1)   WP_132045608.1 WP_132045608.1 MULTISPECIES: response regulator transcription factor
8(1)   WP_042282401.1 WP_042282401.1 response regulator transcription factor
8(1)   WP_320965096.1 WP_320965096.1 helix-turn-helix transcriptional regulator
8(1)   WP_269689511.1 WP_269689511.1 response regulator transcription factor
8(1)   WP_176522975.1 WP_176522975.1 AAA family ATPase
8(1)   WP_222128412.1 WP_222128412.1 response regulator transcription factor
8(1)   WP_138874950.1 WP_138874950.1 response regulator transcription factor

8(1)   WP_045876249.1 WP_045876249.1 response regulator transcription factor
8(1)   WP_156741175.1 WP_156741175.1 response regulator transcription factor
8(1)   WP_096288655.1 WP_096288655.1 LuxR C-terminal-related transcriptional regulator
8(1)   WP_204408547.1 WP_204408547.1 response regulator transcription factor
8(1)   WP_096288653.1 WP_096288653.1 helix-turn-helix transcriptional regulator

9(1)   WP_056154328.1 WP_056154328.1 MULTISPECIES: MDR family MFS transporter
9(1)   WP_056154344.1 WP_056154344.1 MULTISPECIES: MFS transporter
9(1)   WP_134352454.1 WP_134352454.1 MFS transporter
9(1)   WP_056154321.1 WP_056154321.1 MULTISPECIES: MFS transporter
9(1)   WP_266811468.1 WP_266811468.1 MFS transporter
9(1)   WP_266814572.1 WP_266814572.1 MFS transporter
9(1)   WP_259808660.1 WP_259808660.1 MFS transporter
9(1)   WP_259808659.1 WP_259808659.1 MFS transporter
9(1)   WP_045876247.1 WP_045876247.1 multidrug effflux MFS transporter
9(1)   WP_243699659.1 WP_243699659.1 MFS transporter
9(1)   WP_264721889.1 WP_264721889.1 MFS transporter
9(1)   WP_261557239.1 WP_261557239.1 MFS transporter
9(1)   WP_251747724.1 WP_251747724.1 MFS transporter, partial
9(1)   WP_150241378.1 WP_150241378.1 MDR family MFS transporter
9(1)   WP_161111106.1 WP_161111106.1 MFS transporter

10(1)   WP_043568076.1 WP_043568076.1 SDR family NAD(P)-dependent oxidoreductase
10(1)   WP_280895070.1 WP_280895070.1 SDR family oxidoreductase
10(1)   WP_019290309.1 WP_019290309.1 SDR family oxidoreductase
10(1)   WP_138874973.1 WP_138874973.1 SDR family

NAD(P)-dependent oxidoreductase
10(1) WP_308739403.1 WP_308739403.1 SDR family oxidoreductase
10(1) WP_134352471.1 WP_134352471.1 bifunctional aldolase/short-chain dehydrogenase
10(1) WP_110050920.1 WP_110050920.1 SDR family oxidoreductase
10(1) WP_280850619.1 WP_280850619.1 SDR family oxidoreductase
10(1) WP_010694480.1 WP_010694480.1 SDR family oxidoreductase
10(1) WP_131885646.1 WP_131885646.1 SDR family NAD(P)-dependent oxidoreductase
10(1) WP_045876238.1 WP_045876238.1 SDR family NAD(P)-dependent oxidoreductase
10(1) WP_029289520.1 WP_029289520.1 SDR family oxidoreductase
10(1) WP_010694464.1 WP_010694464.1 3-oxoacyl-ACP reductase FabG
10(1) WP_010694481.1 WP_010694481.1 SDR family oxidoreductase
10(1) WP_225503935.1 WP_225503935.1 SDR family oxidoreductase

11(1) WP_205653540.1 WP_205653540.1 DEAD/DEAH box helicase
11(1) WP_310112469.1 WP_310112469.1 DUF3427 domain-containing protein
11(1) WP_259808642.1 WP_259808642.1 DEAD/DEAH box helicase
11(1) WP_312368656.1 WP_312368656.1 type I restriction endonuclease subunit R
11(1) WP_144679692.1 WP_144679692.1 DEAD/DEAH box helicase
11(1) WP_229694063.1 WP_229694063.1 DEAD/DEAH box helicase family protein
11(1) WP_205653542.1 WP_205653542.1 DEAD/DEAH box helicase
11(1) WP_064065949.1 WP_064065949.1 DEAD/DEAH box helicase family protein
11(1) WP_131166524.1 WP_131166524.1 type I restriction endonuclease subunit R
11(1) WP_259815460.1 WP_259815460.1 DEAD/DEAH box helicase family protein
11(1) WP_308739414.1 WP_308739414.1 DEAD/DEAH box helicase

family protein
11(1)   WP_259308333.1 WP_259308333.1 type I restriction endonuclease subunit R
11(1)   WP_072786296.1 WP_072786296.1 DEAD/DEAH box helicase

12(1)   WP_246906735.1 WP_246906735.1 alpha/beta fold hydrolase
12(1)   WP_087509457.1 WP_087509457.1 MULTISPECIES: alpha/beta hydrolase
12(1)   WP_166527637.1 WP_166527637.1 alpha/beta hydrolase
12(1)   WP_068262920.1 WP_068262920.1 alpha/beta hydrolase
12(1)   WP_231955282.1 WP_231955282.1 alpha/beta hydrolase
12(1)   WP_263023560.1 WP_263023560.1 alpha/beta hydrolase
12(1)   WP_061228369.1 WP_061228369.1 alpha/beta fold hydrolase
12(1)   WP_065022122.1 WP_065022122.1 haloalkane dehalogenase

12(1)   WP_148758582.1 WP_148758582.1 alpha/beta hydrolase

13(1)   WP_244980173.1 WP_244980173.1 transposase family protein
13(1)   WP_266518740.1 WP_266518740.1 transposase
13(1)   WP_223927697.1 WP_223927697.1 transposase family protein
13(1)   WP_212323263.1 WP_212323263.1 transposase family protein
13(1)   WP_212324179.1 WP_212324179.1 hypothetical protein
13(1)   WP_233975594.1 WP_233975594.1 transposase
13(1)   WP_244980174.1 WP_244980174.1 transposase
13(1)   WP_263407058.1 WP_263407058.1 hypothetical protein
13(1)   WP_212324175.1 WP_212324175.1 transposase family protein

14(1)   WP_208104787.1 WP_208104787.1 methyltransferase domain-containing protein
14(1)   WP_148758597.1 WP_148758597.1 class I SAM-dependent methyltransferase
14(1)   WP_320965091.1 WP_320965091.1 methyltransferase domain-containing protein

14(1)   WP_232303678.1 WP_232303678.1 class I SAM-dependent methyltransferase
14(1)   WP_261557236.1 WP_261557236.1 class I SAM-dependent methyltransferase
14(1)   WP_056154355.1 WP_056154355.1 MULTISPECIES: class I SAM-dependent methyltransferase
14(1)   WP_147794784.1 WP_147794784.1 arsenite methyltransferase
14(1)   WP_201739586.1 WP_201739586.1 class I SAM-dependent methyltransferase
14(1)   WP_251747720.1 WP_251747720.1 class I SAM-dependent methyltransferase

15(1)   WP_202423048.1 WP_202423048.1 histidine kinase
15(1)   WP_166527619.1 WP_166527619.1 sensor histidine kinase

15(1)   WP_156741174.1 WP_156741174.1 histidine kinase
15(1)   WP_210650324.1 WP_210650324.1 ATP-binding protein
15(1)   WP_256095505.1 WP_256095505.1 histidine kinase
15(1)   WP_161111100.1 WP_161111100.1 histidine kinase
15(1)   WP_269689510.1 WP_269689510.1 sensor domain-containing protein
15(1)   WP_259804724.1 WP_259804724.1 histidine kinase

16(1)   WP_279436312.1 WP_279436312.1 serine/threonine-protein kinase
16(1)   WP_014373959.1 WP_014373959.1 protein kinase
16(1)   WP_251747719.1 WP_251747719.1 serine/threonine-protein kinase, partial
16(1)   WP_014373958.1 WP_014373958.1 Stk1 family PASTA domain-containing Ser/Thr kinase
16(1)   WP_180903689.1 WP_180903689.1 serine/threonine protein kinase
16(1)   WP_134850829.1 WP_134850829.1 lanthionine synthetase LanC family protein
16(1)   WP_261557238.1 WP_261557238.1 serine/threonine-protein kinase, partial
16(1)   WP_205653535.1 WP_205653535.1 BREX system serine/threonine kinase PglW

17(1)   WP_283663869.1 WP_283663869.1 Lrp/AsnC family transcriptional regulator
17(1)   WP_205653550.1 WP_205653550.1 Lrp/AsnC family transcriptional regulator
17(1)   WP_070320115.1 WP_070320115.1 MULTISPECIES: Lrp/AsnC family transcriptional regulator
17(1)   WP_189798729.1 WP_189798729.1 Lrp/AsnC family transcriptional regulator
17(1)   WP_310112446.1 WP_310112446.1 hypothetical protein
17(1)   WP_031023264.1 WP_031023264.1 MULTISPECIES: Lrp/AsnC family transcriptional regulator
17(1)   WP_310112450.1 WP_310112450.1 Lrp/AsnC family transcriptional regulator
17(1)   WP_199424241.1 WP_199424241.1 Lrp/AsnC ligand binding domain-containing protein

18(1)   WP_234538978.1 WP_234538978.1 hypothetical protein
18(1)   WP_057608710.1 WP_057608710.1 hypothetical protein
18(1)   WP_069764278.1 WP_069764278.1 MULTISPECIES: hypothetical protein
18(1)   WP_062244627.1 WP_062244627.1 MULTISPECIES: hypothetical protein
18(1)   WP_168374672.1 WP_168374672.1 hypothetical protein
18(1)   WP_189481973.1 WP_189481973.1 hypothetical protein
18(1)   WP_014673667.1 WP_014673667.1 MULTISPECIES: hypothetical protein

19(1)   WP_234538967.1 WP_234538967.1 replication initiator protein RepSA
19(1)   WP_189481961.1 WP_189481961.1 replication initiator protein RepSA
19(1)   WP_266515797.1 WP_266515797.1 replication initiator protein RepSA
19(1)   WP_057608716.1 WP_057608716.1 hypothetical protein
19(1)   WP_168374676.1 WP_168374676.1 replication initiation protein
19(1)   WP_141786870.1 WP_141786870.1 replication initiation protein
19(1)   WP_039654749.1 WP_039654749.1 hypothetical protein

20(1)   WP_168374675.1 WP_168374675.1 SpdD protein
20(1)   WP_266515803.1 WP_266515803.1 SpdD-like protein
20(1)   WP_234538973.1 WP_234538973.1 SpdD-like protein
20(1)   WP_039654750.1 WP_039654750.1 MULTISPECIES: hypothetical protein
20(2)   WP_057608715.1 WP_057608715.1 MULTISPECIES: hypothetical protein
20(1)   WP_189481964.1 WP_189481964.1 SpdD-like protein

21(1)   WP_269689504.1 WP_269689504.1 SigE family RNA polymerase sigma factor
21(1)   WP_131166527.1 WP_131166527.1 sigma-70 family RNA polymerase sigma factor
21(1)   WP_243401080.1 WP_243401080.1 MULTISPECIES: sigma-70 family RNA polymerase sigma factor
21(1)   WP_197536251.1 WP_197536251.1 RNA polymerase sigma factor
21(1)   WP_189159260.1 WP_189159260.1 sigma-70 family RNA polymerase sigma factor
21(1)   WP_132045647.1 WP_132045647.1 RNA polymerase sigma factor
21(1)   WP_148240309.1 WP_148240309.1 SigE family RNA polymerase sigma factor

22(1)   WP_039654753.1 WP_039654753.1 DUF2637 domain-containing protein
22(1)   WP_057608712.1 WP_057608712.1 DUF2637 domain-containing protein
22(1)   WP_234538976.1 WP_234538976.1 DUF2637 domain-containing protein
22(1)   WP_189481968.1 WP_189481968.1 DUF2637 domain-containing protein
22(1)   WP_280911793.1 WP_280911793.1 DUF2637 domain-containing protein
22(1)   WP_168374674.1 WP_168374674.1 DUF2637 domain-containing protein
22(1)   WP_266515810.1 WP_266515810.1 DUF2637 domain-containing protein

23(1) WP_137961317.1 WP_137961317.1 MULTISPECIES: mobile element transfer protein
23(1) WP_057608713.1 WP_057608713.1 mobile element transfer protein
23(1) WP_189481966.1 WP_189481966.1 MULTISPECIES: mobile element transfer protein
23(1) WP_039654752.1 WP_039654752.1 MULTISPECIES: Mobile element transfer
23(1) WP_046708309.1 WP_046708309.1 MULTISPECIES: mobile element transfer protein
23(1) WP_280911794.1 WP_280911794.1 mobile element transfer protein
23(1) WP_234538975.1 WP_234538975.1 mobile element transfer protein

24(1) WP_320965095.1 WP_320965095.1 dTDP-4-dehydrorhamnose reductase
24(1) WP_320965094.1 WP_320965094.1 dTDP-glucose 4,6-dehydratase
24(1) WP_128470515.1 WP_128470515.1 dTDP-glucose 4,6-dehydratase
24(1) WP_128470514.1 WP_128470514.1 sugar nucleotide-binding protein
24(1) WP_320965372.1 WP_320965372.1 NAD-dependent epimerase/dehydratase family protein
24(1) WP_138874975.1 WP_138874975.1 NAD(P)-dependent oxidoreductase
24(1) WP_191022563.1 WP_191022563.1 GDP-mannose 4,6-dehydratase

25(1) WP_149382905.1 WP_149382905.1 MlaD family protein
25(1) WP_149382902.1 WP_149382902.1 MCE family protein
25(1) WP_149382900.1 WP_149382900.1 MCE family protein
25(1) WP_149382904.1 WP_149382904.1 MCE family protein
25(1) WP_149382903.1 WP_149382903.1 MCE family protein
25(1) WP_149382901.1 WP_149382901.1 MCE family protein

26(1) WP_237710511.1 WP_237710511.1 TIGR03619 family F420-dependent LLM class oxidoreductase
26(1) WP_045876250.1 WP_045876250.1 LLM class F420-dependent oxidoreductase
26(1) WP_150241418.1 WP_150241418.1 LLM class flavin-dependent oxidoreductase
26(1) WP_149382918.1 WP_149382918.1 LLM class F420-dependent oxidoreductase
26(1) WP_210650319.1 WP_210650319.1 LLM class F420-dependent oxidoreductase
26(1) WP_067761229.1 WP_067761229.1 MULTISPECIES: LLM class flavin-dependent oxidoreductase

27(1) WP_269694697.1 WP_269694697.1 nucleoside deaminase
27(1) WP_278182065.1 WP_278182065.1 tRNA adenosine(34) deaminase TadA
27(1) WP_029108588.1 WP_029108588.1 nucleoside deaminase
27(1) WP_264721899.1 WP_264721899.1 nucleoside deaminase
27(1) WP_065022099.1 WP_065022099.1 nucleoside deaminase
27(1) WP_263023565.1 WP_263023565.1 nucleoside deaminase

28(1) WP_278177381.1 WP_278177381.1 FG-GAP and VCBS repeat-containing protein
28(1) WP_278177382.1 WP_278177382.1 FG-GAP-like repeat-containing protein
28(1) WP_280911777.1 WP_280911777.1 FG-GAP-like repeat-containing protein
28(1) WP_069740787.1 WP_069740787.1 VCBS repeat-containing protein
28(2) WP_266761005.1 WP_266761005.1 MULTISPECIES: FG-GAP-like repeat-containing protein

29(1) WP_045876239.1 WP_045876239.1 NAD(P)-dependent alcohol dehydrogenase
29(1) WP_186381201.1 WP_186381201.1 alcohol dehydrogenase catalytic domain-containing protein
29(1) WP_283663873.1 WP_283663873.1 NADP-dependent oxidoreductase
29(1) WP_246906737.1 WP_246906737.1 zinc-binding

dehydrogenase
29(1)   WP_212324232.1 WP_212324232.1 NADP-dependent oxidoreductase
29(1)   WP_205653552.1 WP_205653552.1 NADP-dependent oxidoreductase

30(1)   WP_266811482.1 WP_266811482.1 helix-turn-helix domain-containing protein
30(1)   WP_250358781.1 WP_250358781.1 helix-turn-helix domain-containing protein
30(1)   WP_283663888.1 WP_283663888.1 helix-turn-helix domain-containing protein
30(1)   WP_141786869.1 WP_141786869.1 helix-turn-helix domain-containing protein
30(1)   WP_031113746.1 WP_031113746.1 helix-turn-helix domain-containing protein
30(1)   WP_031017537.1 WP_031017537.1 MULTISPECIES: helix-turn-helix domain-containing protein

31(1)   WP_010694476.1 WP_010694476.1 acyl-CoA dehydrogenase family protein
31(1)   WP_210650317.1 WP_210650317.1 acyl-CoA dehydrogenase family protein
31(1)   WP_014373967.1 WP_014373967.1 acyl-CoA dehydrogenase family protein
31(1)   WP_010694462.1 WP_010694462.1 acyl-CoA dehydrogenase family protein
31(1)   WP_010694474.1 WP_010694474.1 acyl-CoA dehydrogenase family protein
31(1)   WP_308739418.1 WP_308739418.1 acyl-CoA dehydrogenase family protein

32(1)   WP_010965223.1 WP_010965223.1 MULTISPECIES: single-stranded DNA-binding protein
32(1)   WP_255847325.1 WP_255847325.1 single-stranded DNA-binding protein
32(1)   WP_185275396.1 WP_185275396.1 single-stranded DNA-binding protein
32(1)   WP_255847333.1 WP_255847333.1 single-stranded

DNA-binding protein
32(1)   WP_156044110.1 WP_156044110.1 single-stranded DNA-binding protein

33(2)   WP_266761036.1 WP_266761036.1 MULTISPECIES: hypothetical protein
33(1)   WP_263023584.1 WP_263023584.1 hypothetical protein
33(1)   WP_266518752.1 WP_266518752.1 hypothetical protein
33(1)   WP_266811498.1 WP_266811498.1 hypothetical protein

34(1)   WP_266515851.1 WP_266515851.1 cytochrome P450
34(1)   WP_157488743.1 WP_157488743.1 cytochrome P450
34(1)   WP_189159264.1 WP_189159264.1 cytochrome P450
34(1)   WP_052710763.1 WP_052710763.1 cytochrome P450
34(1)   WP_278177384.1 WP_278177384.1 cytochrome P450

35(1)   WP_057608707.1 WP_057608707.1 ATP-binding protein
35(1)   WP_280911790.1 WP_280911790.1 ATP-binding protein
35(1)   WP_057608696.1 WP_057608696.1 ATP-binding protein
35(1)   WP_168374670.1 WP_168374670.1 ATP-binding protein
35(1)   WP_039654757.1 WP_039654757.1 MULTISPECIES: ATP-binding protein

36(1)   WP_168374677.1 WP_168374677.1 helix-turn-helix domain-containing protein
36(1)   WP_053750100.1 WP_053750100.1 MULTISPECIES: helix-turn-helix domain-containing protein
36(1)   WP_039654748.1 WP_039654748.1 helix-turn-helix domain-containing protein
36(1)   WP_148758595.1 WP_148758595.1 helix-turn-helix domain-containing protein
36(1)   WP_280911796.1 WP_280911796.1 excisionase family DNA-binding protein

37(1)   WP_250358784.1 WP_250358784.1 plasmid replication, integration and excision activator
37(1)   WP_132045620.1 WP_132045620.1 plasmid replication,

integration and excision activator
37(1) WP_283663883.1 WP_283663883.1 hypothetical protein
37(1) WP_101788513.1 WP_101788513.1 plasmid replication, integration and excision activator
37(1) WP_221632386.1 WP_221632386.1 plasmid replication, integration and excision activator

38(1) WP_256843042.1 WP_256843042.1 transposase family protein
38(1) WP_244980175.1 WP_244980175.1 transposase
38(1) WP_244980176.1 WP_244980176.1 ISAs1 family transposase
38(1) WP_306239619.1 WP_306239619.1 ISAs1 family transposase
38(1) WP_212324177.1 WP_212324177.1 ISAs1 family transposase

39(1) WP_241656147.1 WP_241656147.1 class I SAM-dependent DNA methyltransferase
39(1) WP_189159272.1 WP_189159272.1 N-6 DNA methylase
39(1) WP_259815462.1 WP_259815462.1 class I SAM-dependent DNA methyltransferase
39(1) WP_308739412.1 WP_308739412.1 class I SAM-dependent DNA methyltransferase
39(1) WP_312368653.1 WP_312368653.1 type I restriction-modification system subunit M

40(1) WP_096288633.1 WP_096288633.1 MULTISPECIES: acetolactate synthase large subunit
40(1) WP_320965086.1 WP_320965086.1 acetolactate synthase large subunit
40(1) WP_205653551.1 WP_205653551.1 thiamine pyrophosphate-binding protein
40(1) WP_310112445.1 WP_310112445.1 thiamine pyrophosphate-binding protein
40(1) WP_038991385.1 WP_038991385.1 MULTISPECIES: acetolactate synthase large subunit

41(1)   WP_029289491.1 WP_029289491.1 GTPase
41(1)   WP_051681775.1 WP_051681775.1 GTPase domain-containing protein
41(1)   WP_081861536.1 WP_081861536.1 dynamin family protein
41(1)   WP_034648421.1 WP_034648421.1 GTPase

42(1)   WP_198152830.1 WP_198152830.1 aldehyde dehydrogenase family protein
42(1)   WP_189798728.1 WP_189798728.1 gamma-aminobutyraldehyde dehydrogenase
42(1)   WP_189481993.1 WP_189481993.1 gamma-aminobutyraldehyde dehydrogenase
42(1)   WP_017545778.1 WP_017545778.1 MULTISPECIES: NAD-dependent succinate-semialdehyde dehydrogenase

43(1)   WP_056915133.1 WP_056915133.1 hypothetical protein
43(1)   WP_155946647.1 WP_155946647.1 restriction endonuclease
43(1)   WP_213011318.1 WP_213011318.1 hypothetical protein
43(1)   WP_225503929.1 WP_225503929.1 hypothetical protein

44(1)   WP_056154361.1 WP_056154361.1 MULTISPECIES: dihydrofolate reductase family protein
44(1)   WP_205653554.1 WP_205653554.1 dihydrofolate reductase family protein
44(1)   WP_097195741.1 WP_097195741.1 dihydrofolate reductase family protein
44(1)   WP_246906746.1 WP_246906746.1 dihydrofolate reductase family protein

45(1)   WP_096288657.1 WP_096288657.1 sugar transferase
45(1)   WP_256095506.1 WP_256095506.1 sugar transferase
45(1)   WP_118375147.1 WP_118375147.1 sugar transferase
45(1)   WP_053797892.1 WP_053797892.1 sugar transferase

46(1)   WP_259804727.1 WP_259804727.1 sugar ABC transporter permease

46(1)   WP_093738715.1 WP_093738715.1 MULTISPECIES: sugar ABC transporter permease
46(1)   WP_093738713.1 WP_093738713.1 MULTISPECIES: carbohydrate ABC transporter permease
46(1)   WP_259804728.1 WP_259804728.1 carbohydrate ABC transporter permease

47(1)   WP_266518733.1 WP_266518733.1 IS5 family transposase
47(2)   WP_266755043.1 WP_266755043.1 MULTISPECIES: IS5 family transposase
47(1)   WP_266520501.1 WP_266520501.1 IS5 family transposase

48(2)   WP_266760997.1 WP_266760997.1 MULTISPECIES: putative PEP-binding protein
48(1)   WP_166527636.1 WP_166527636.1 phosphoenolpyruvate synthase
48(1)   WP_205653558.1 WP_205653558.1 phosphoenolpyruvate synthase

49(2)   WP_266761001.1 WP_266761001.1 MULTISPECIES: dihydroxyacetone kinase subunit DhaL
49(2)   WP_266760995.1 WP_266760995.1 MULTISPECIES: dihydroxyacetone kinase subunit DhaL

50(2)   WP_266761028.1 WP_266761028.1 MULTISPECIES: GGDEF domain-containing protein
50(1)   WP_045876251.1 WP_045876251.1 bifunctional diguanylate cyclase/phosphodiesterase
50(1)   WP_189481962.1 WP_189481962.1 MULTISPECIES: GGDEF domain-containing protein

51(1)   WP_208526487.1 WP_208526487.1 bifunctional DNA primase/polymerase
51(1)   WP_266518755.1 WP_266518755.1 bifunctional DNA primase/polymerase
51(2)   WP_266761044.1 WP_266761044.1 MULTISPECIES: bifunctional DNA primase/polymerase

52(2)   WP_266761042.1 WP_266761042.1 MULTISPECIES: DNA cytosine methyltransferase
52(2)   WP_266765782.1 WP_266765782.1 MULTISPECIES: SAM-dependent methyltransferase

53(1)   WP_266520505.1 WP_266520505.1 DUF2637 domain-containing protein
53(1)   WP_266811506.1 WP_266811506.1 DUF2637 domain-containing protein
53(2)   WP_266761030.1 WP_266761030.1 MULTISPECIES: DUF2637 domain-containing protein

54(1)   WP_010694479.1 WP_010694479.1 TetR/AcrR family transcriptional regulator
54(1)   WP_051288858.1 WP_051288858.1 TetR/AcrR family transcriptional regulator
54(1)   WP_048342053.1 WP_048342053.1 TetR/AcrR family transcriptional regulator
54(1)   WP_278177395.1 WP_278177395.1 TetR/AcrR family transcriptional regulator

55(1)   WP_004986817.1 WP_004986817.1 MULTISPECIES: hypothetical protein
55(1)   WP_039654751.1 WP_039654751.1 hypothetical protein
55(1)   WP_057608714.1 WP_057608714.1 hypothetical protein
55(1)   WP_280911795.1 WP_280911795.1 hypothetical protein

56(1)   WP_280913369.1 WP_280913369.1 hypothetical protein
56(1)   WP_039654758.1 WP_039654758.1 hypothetical protein
56(1)   WP_168374679.1 WP_168374679.1 hypothetical protein
56(1)   WP_057608837.1 WP_057608837.1 hypothetical protein

57(1)   WP_147918738.1 WP_147918738.1 substrate-binding domain-containing protein
57(1)   WP_308739402.1 WP_308739402.1 LacI family DNA-binding

transcriptional regulator
57(1)   WP_259843035.1 WP_259843035.1 LacI family DNA-binding transcriptional regulator
57(1)   WP_047524222.1 WP_047524222.1 MULTISPECIES: LacI family DNA-binding transcriptional regulator

58(1)   WP_235547684.1 WP_235547684.1 MULTISPECIES: metalloregulator ArsR/SmtB family transcription factor
58(1)   WP_312103511.1 WP_312103511.1 N-acetyltransferase family protein
58(1)   WP_168216914.1 WP_168216914.1 GNAT family N-acetyltransferase
58(1)   WP_315912320.1 WP_315912320.1 GNAT family N-acetyltransferase

59(1)   WP_244980196.1 WP_244980196.1 NAD(P)H-binding protein

59(1)   WP_320965078.1 WP_320965078.1 SDR family oxidoreductase
59(1)   WP_081861535.1 WP_081861535.1 SDR family oxidoreductase
59(1)   WP_201054846.1 WP_201054846.1 SDR family oxidoreductase

60(1)   WP_082937608.1 WP_082937608.1 MULTISPECIES: prephenate dehydrogenase
60(1)   WP_029108590.1 WP_029108590.1 prephenate dehydrogenase
60(1)   WP_064900713.1 WP_064900713.1 prephenate dehydrogenase

61(1)   WP_084229285.1 WP_084229285.1 phosphotransferase family protein
61(1)   WP_010694460.1 WP_010694460.1 phosphotransferase family protein
61(1)   WP_038567543.1 WP_038567543.1 phosphotransferase family protein

62(1) WP_070780165.1 WP_070780165.1 MULTISPECIES: Clp protease ClpP
62(1) WP_046528531.1 WP_046528531.1 MULTISPECIES: ATP-dependent Clp protease proteolytic subunit
62(1) WP_087509459.1 WP_087509459.1 MULTISPECIES: ATP-dependent Clp protease proteolytic subunit

63(1) WP_038991387.1 WP_038991387.1 MULTISPECIES: acetolactate synthase small subunit
63(1) WP_053797884.1 WP_053797884.1 MULTISPECIES: acetolactate synthase small subunit
63(1) WP_096288635.1 WP_096288635.1 MULTISPECIES: acetolactate synthase small subunit

64(1) WP_134850841.1 WP_134850841.1 alanine dehydrogenase
64(1) WP_056154316.1 WP_056154316.1 MULTISPECIES: NAD(P) transhydrogenase subunit alpha
64(1) WP_096288627.1 WP_096288627.1 N(5)-(carboxyethyl)ornithine synthase

65(1) WP_138874951.1 WP_138874951.1 HAMP domain-containing sensor histidine kinase
65(1) WP_110050900.1 WP_110050900.1 HAMP domain-containing sensor histidine kinase
65(1) WP_045876248.1 WP_045876248.1 HAMP domain-containing sensor histidine kinase

66(1) WP_143914088.1 WP_143914088.1 hypothetical protein
66(1) WP_143914086.1 WP_143914086.1 hypothetical protein
66(1) WP_143914089.1 WP_143914089.1 hypothetical protein

67(1) WP_147794786.1 WP_147794786.1 ATP-dependent DNA ligase
67(1) WP_147794805.1 WP_147794805.1 ATP-dependent DNA ligase
67(1) WP_147794806.1 WP_147794806.1 ATP-dependent DNA

ligase

68(1) WP_188947919.1 WP_188947919.1 hypothetical protein
68(1) WP_096288629.1 WP_096288629.1 hypothetical protein
68(1) WP_161567488.1 WP_161567488.1 hypothetical protein

69(1) WP_128470526.1 WP_128470526.1 lycopene cyclase domain-containing protein
69(1) WP_096288619.1 WP_096288619.1 MULTISPECIES: lycopene cyclase domain-containing protein
69(1) WP_188947923.1 WP_188947923.1 lycopene cyclase domain-containing protein

70(1) WP_189481991.1 WP_189481991.1 aspartate aminotransferase family protein
70(1) WP_017545779.1 WP_017545779.1 MULTISPECIES: aminotransferase class III-fold pyridoxal phosphate-dependent enzyme
70(1) WP_189798744.1 WP_189798744.1 aspartate aminotransferase family protein

71(1) WP_017534492.1 WP_017534492.1 serine hydrolase domain-containing protein
71(1) WP_147918732.1 WP_147918732.1 serine hydrolase domain-containing protein
71(1) WP_201054838.1 WP_201054838.1 serine hydrolase domain-containing protein

72(1) WP_204677503.1 WP_204677503.1 isopentenyl-diphosphate Delta-isomerase
72(1) WP_096288625.1 WP_096288625.1 isopentenyl-diphosphate Delta-isomerase
72(1) WP_128470524.1 WP_128470524.1 isopentenyl-diphosphate Delta-isomerase

73(1) WP_205653553.1 WP_205653553.1 SulP family inorganic

anion transporter
73(1)   WP_147918734.1 WP_147918734.1 SulP family inorganic anion transporter
73(1)   WP_097195739.1 WP_097195739.1 SulP family inorganic anion transporter

74(1)   WP_006552534.1 WP_006552534.1 MULTISPECIES: metallophosphoesterase
74(1)   WP_184443033.1 WP_184443033.1 metallophosphoesterase
74(1)   WP_210650301.1 WP_210650301.1 metallophosphoesterase

75(1)   WP_148758580.1 WP_148758580.1 helix-turn-helix domain-containing protein
75(1)   WP_199424258.1 WP_199424258.1 DJ-1/PfpI family protein
75(1)   WP_223927699.1 WP_223927699.1 DJ-1/PfpI family protein

76(1)   WP_234420178.1 WP_234420178.1 HNH endonuclease
76(1)   WP_222128411.1 WP_222128411.1 HNH endonuclease
76(1)   WP_222128410.1 WP_222128410.1 hypothetical protein

77(1)   WP_082156215.1 WP_082156215.1 SpoIIE family protein phosphatase
77(1)   WP_234538993.1 WP_234538993.1 PP2C family protein-serine/threonine phosphatase
77(1)   WP_097195742.1 WP_097195742.1 SpoIIE family protein phosphatase

78(1)   WP_235530755.1 WP_235530755.1 recombinase family protein
78(1)   WP_052336343.1 WP_052336343.1 recombinase family protein
78(1)   WP_168932136.1 WP_168932136.1 recombinase family protein

79(1)   WP_006126276.1 WP_006126276.1 MULTISPECIES: dCTP deaminase
79(1)   WP_259308323.1 WP_259308323.1 dCTP deaminase
79(1)   WP_047524231.1 WP_047524231.1 MULTISPECIES: dCTP deaminase

80(1)   WP_138874971.1 WP_138874971.1 phosphodiester glycosidase family protein
80(1)   WP_259808641.1 WP_259808641.1 phosphodiester glycosidase family protein
80(1)   WP_210650306.1 WP_210650306.1 phosphodiester glycosidase family protein

81(1)   WP_134352469.1 WP_134352469.1 type 1 glutamine amidotransferase domain-containing protein
81(1)   WP_131885637.1 WP_131885637.1 type 1 glutamine amidotransferase domain-containing protein
81(1)   WP_259808663.1 WP_259808663.1 type 1 glutamine amidotransferase domain-containing protein

82(1)   WP_052270093.1 WP_052270093.1 MULTISPECIES: BlaI/MecI/CopY family transcriptional regulator
82(1)   WP_261557229.1 WP_261557229.1 BlaI/MecI/CopY family transcriptional regulator
82(1)   WP_261557225.1 WP_261557225.1 BlaI/MecI/CopY family transcriptional regulator

83(1)   WP_243718311.1 WP_243718311.1 PadR family transcriptional regulator
83(1)   WP_259308346.1 WP_259308346.1 PadR family transcriptional regulator
83(1)   WP_263023561.1 WP_263023561.1 PadR family transcriptional regulator

84(1)   WP_036475967.1 WP_036475967.1 hypothetical protein
84(1)   WP_185275407.1 WP_185275407.1 hypothetical protein
84(1)   WP_264721894.1 WP_264721894.1 helix-turn-helix

domain-containing protein

85(1)   WP_264721900.1 WP_264721900.1 tRNA adenosine deaminase-associated protein
85(1)   WP_003883664.1 WP_003883664.1 MULTISPECIES: tRNA adenosine deaminase-associated protein
85(1)   WP_029108589.1 WP_029108589.1 tRNA adenosine deaminase-associated protein

86(1)   WP_150241428.1 WP_150241428.1 allophanate hydrolase
86(1)   WP_264721901.1 WP_264721901.1 amidase
86(1)   WP_017545772.1 WP_017545772.1 MULTISPECIES: amidase

87(1)   WP_234538972.1 WP_234538972.1 hypothetical protein
87(1)   WP_266515800.1 WP_266515800.1 hypothetical protein
87(1)   WP_078634963.1 WP_078634963.1 MULTISPECIES: hypothetical protein

88(1)   WP_137308903.1 WP_137308903.1 MULTISPECIES: hypothetical protein
88(1)   WP_234538974.1 WP_234538974.1 DUF4175 domain-containing protein
88(1)   WP_266515806.1 WP_266515806.1 hypothetical protein

89(1)   WP_199424261.1 WP_199424261.1 aldo/keto reductase family protein
89(1)   WP_266518617.1 WP_266518617.1 aldo/keto reductase
89(1)   WP_259808636.1 WP_259808636.1 aldo/keto reductase

90(2)   WP_266760999.1 WP_266760999.1 MULTISPECIES: dihydroxyacetone kinase subunit DhaK
90(1)   WP_266760993.1 WP_266760993.1 MULTISPECIES: dihydroxyacetone kinase subunit DhaK

91(1)   WP_141786880.1 WP_141786880.1 DUF2750

domain-containing protein
91(2)   WP_266761007.1 WP_266761007.1 MULTISPECIES: DUF2750 domain-containing protein

92(1)   WP_266518748.1 WP_266518748.1 hypothetical protein
92(2)   WP_266761026.1 WP_266761026.1 MULTISPECIES: hypothetical protein

93(1)   WP_166527618.1 WP_166527618.1 ABC transporter permease
93(1)   WP_048342049.1 WP_048342049.1 MULTISPECIES: ABC transporter permease
93(1)   WP_266811463.1 WP_266811463.1 ABC transporter permease

94(1)   WP_175337891.1 WP_175337891.1 MULTISPECIES: DUF305 domain-containing protein
94(1)   WP_266811475.1 WP_266811475.1 DUF305 domain-containing protein
94(1)   WP_097195718.1 WP_097195718.1 DUF305 domain-containing protein

95(1)   WP_154346777.1 WP_154346777.1 DNA cytosine methyltransferase
95(1)   WP_161302359.1 WP_161302359.1 MULTISPECIES: DNA cytosine methyltransferase
95(1)   WP_266811502.1 WP_266811502.1 DNA cytosine methyltransferase

96(1)   WP_223830458.1 WP_223830458.1 helix-turn-helix transcriptional regulator
96(1)   WP_278177392.1 WP_278177392.1 helix-turn-helix transcriptional regulator
96(1)   WP_237710510.1 WP_237710510.1 Scr1 family TA system antitoxin-like transcriptional regulator

97(1) WP_205653532.1 WP_205653532.1 helicase-related protein
97(1) WP_197080757.1 WP_197080757.1 helicase-related protein
97(1) WP_304487060.1 WP_304487060.1 helicase-related protein

98(1) WP_306239620.1 WP_306239620.1 DnaJ domain-containing protein
98(1) WP_256843046.1 WP_256843046.1 hypothetical protein
98(1) WP_213011326.1 WP_213011326.1 hypothetical protein

99(1) WP_144679704.1 WP_144679704.1 DUF262 domain-containing protein
99(1) WP_310112448.1 WP_310112448.1 DUF262 domain-containing protein
99(1) WP_053071551.1 WP_053071551.1 DUF262 domain-containing protein

100(1) WP_310112460.1 WP_310112460.1 APC family permease
100(1) WP_310112453.1 WP_310112453.1 amino acid permease
100(1) WP_205653548.1 WP_205653548.1 amino acid permease

101(1) WP_014373979.1 WP_014373979.1 carbon-nitrogen hydrolase
101(1) WP_310112458.1 WP_310112458.1 carbon-nitrogen hydrolase family protein
101(1) WP_310112462.1 WP_310112462.1 carbon-nitrogen hydrolase family protein

102(1) WP_118056559.1 WP_118056559.1 MULTISPECIES: glutamine-hydrolyzing GMP synthase
102(1) WP_014373957.1 WP_014373957.1 aminodeoxychorismate/anthranilate synthase component II
102(1) WP_312102786.1 WP_312102786.1 glutamine-hydrolyzing GMP synthase

103(1) WP_057608698.1 WP_057608698.1 IS3 family transposase
103(1) WP_223927909.1 WP_223927909.1 IS3 family transposase
103(1) WP_312368650.1 WP_312368650.1 IS3 family transposase

104(1) WP_128470530.1 WP_128470530.1 MarR family transcriptional regulator
104(1) WP_320965080.1 WP_320965080.1 MarR family transcriptional regulator
104(1) WP_096288611.1 WP_096288611.1 MarR family transcriptional regulator

105(1) WP_096288613.1 WP_096288613.1 polyprenyl synthetase family protein
105(1) WP_320965081.1 WP_320965081.1 polyprenyl synthetase family protein
105(1) WP_128470529.1 WP_128470529.1 polyprenyl synthetase family protein

106(1) WP_096288615.1 WP_096288615.1 squalene/phytoene synthase family protein
106(1) WP_320965082.1 WP_320965082.1 squalene/phytoene synthase family protein
106(1) WP_128470528.1 WP_128470528.1 squalene/phytoene synthase family protein

107(1) WP_128470527.1 WP_128470527.1 phytoene desaturase family protein
107(1) WP_320965083.1 WP_320965083.1 phytoene desaturase family protein
107(1) WP_096288617.1 WP_096288617.1 phytoene desaturase family protein

108(1) WP_096288621.1 WP_096288621.1 lycopene cyclase domain-containing protein
108(1) WP_128470525.1 WP_128470525.1 lycopene cyclase domain-containing protein

108(1) WP_320965084.1 WP_320965084.1 lycopene cyclase domain-containing protein

109(1) WP_096288623.1 WP_096288623.1 prenyltransferase
109(1) WP_073707539.1 WP_073707539.1 MULTISPECIES: prenyltransferase
109(1) WP_320965085.1 WP_320965085.1 prenyltransferase

110(1) WP_176484284.1 WP_176484284.1 MULTISPECIES: ketol-acid reductoisomerase
110(1) WP_128470521.1 WP_128470521.1 ketol-acid reductoisomerase
110(1) WP_320965087.1 WP_320965087.1 ketol-acid reductoisomerase

111(1) WP_320965098.1 WP_320965098.1 Gfo/Idh/MocA family oxidoreductase
111(1) WP_272919922.1 WP_272919922.1 Gfo/Idh/MocA family oxidoreductase
111(1) WP_166527614.1 WP_166527614.1 Gfo/Idh/MocA family oxidoreductase

112(1) WP_043666280.1 WP_043666280.1 MULTISPECIES: putative nucleotide-diphospho-sugar transferase
112(1) WP_024040326.1 WP_024040326.1 hypothetical protein

113(2) WP_057608699.1 WP_057608699.1 MULTISPECIES: hypothetical protein

114(1) WP_064065950.1 WP_064065950.1 AAA family ATPase
114(1) WP_064065955.1 WP_064065955.1 DNA sulfur modification protein DndD

115(1) WP_064900709.1 WP_064900709.1 MULTISPECIES: diol dehydratase small subunit

115(1) WP_029108566.1 WP_029108566.1 diol dehydratase small subunit

116(1) WP_064900710.1 WP_064900710.1 MULTISPECIES: wax ester/triacylglycerol synthase family O-acyltransferase
116(1) WP_029108568.1 WP_029108568.1 wax ester/triacylglycerol synthase family O-acyltransferase

117(1) WP_029108569.1 WP_029108569.1 peroxidase family protein
117(1) WP_064900711.1 WP_064900711.1 peroxidase

118(1) WP_036476089.1 WP_036476089.1 propanediol/glycerol family dehydratase large subunit
118(1) WP_065022120.1 WP_065022120.1 propanediol/glycerol family dehydratase large subunit

119(1) WP_029108573.1 WP_029108573.1 nitroreductase family deazaflavin-dependent oxidoreductase
119(1) WP_067747677.1 WP_067747677.1 MULTISPECIES: nitroreductase family deazaflavin-dependent oxidoreductase

120(1) WP_069400055.1 WP_069400055.1 class I SAM-dependent methyltransferase
120(1) WP_036475948.1 WP_036475948.1 SAM-dependent methyltransferase

121(1) WP_101788520.1 WP_101788520.1 LysR family transcriptional regulator
121(1) WP_029289522.1 WP_029289522.1 LysR family transcriptional regulator

122(1) WP_110050918.1 WP_110050918.1 MarR family winged helix-turn-helix transcriptional regulator
122(1) WP_061228375.1 WP_061228375.1 MULTISPECIES: MarR

family transcriptional regulator

123(1) WP_110050924.1 WP_110050924.1
(2,3-dihydroxybenzoyl)adenylate synthase
123(1) WP_110050934.1 WP_110050934.1 non-ribosomal peptide
synthetase

124(1) WP_132045612.1 WP_132045612.1 phospholipid
scramblase-related protein
124(1) WP_132045610.1 WP_132045610.1 phospholipid
scramblase-related protein

125(1) WP_132045653.1 WP_132045653.1 hypothetical protein
125(1) WP_132045655.1 WP_132045655.1 hypothetical protein

126(1) WP_047524259.1 WP_047524259.1 MULTISPECIES: ABC
transporter permease
126(1) WP_134352474.1 WP_134352474.1 ABC transporter
permease

127(1) WP_092727753.1 WP_092727753.1 RusA family crossover
junction endodeoxyribonuclease
127(1) WP_136002631.1 WP_136002631.1 RusA family crossover
junction endodeoxyribonuclease

128(1) WP_010965219.1 WP_010965219.1 MULTISPECIES:
hypothetical protein
128(1) WP_136002633.1 WP_136002633.1 sigma factor-like
helix-turn-helix DNA-binding protein

129(1) WP_092727728.1 WP_092727728.1 hypothetical protein
129(1) WP_136002638.1 WP_136002638.1 hypothetical protein

130(1) WP_092727752.1 WP_092727752.1 PBSX family phage

terminase large subunit
130(1) WP_136002640.1 WP_136002640.1 PBSX family phage terminase large subunit

131(1) WP_141786862.1 WP_141786862.1 competence protein CoiA family protein
131(1) WP_029289516.1 WP_029289516.1 competence protein CoiA family protein

132(1) WP_143914096.1 WP_143914096.1 MULTISPECIES: helix-turn-helix transcriptional regulator
132(1) WP_143914095.1 WP_143914095.1 helix-turn-helix transcriptional regulator

133(1) WP_144679688.1 WP_144679688.1 NERD domain-containing protein
133(1) WP_144679676.1 WP_144679676.1 ATP-binding domain-containing protein

134(1) WP_147918733.1 WP_147918733.1 metalloregulator ArsR/SmtB family transcription factor
134(1) WP_097195740.1 WP_097195740.1 metalloregulator ArsR/SmtB family transcription factor

135(1) WP_030767823.1 WP_030767823.1 MULTISPECIES: HDIG domain-containing protein
135(1) WP_148758589.1 WP_148758589.1 HD domain-containing protein

136(1) WP_107407188.1 WP_107407188.1 MULTISPECIES: asparagine synthase-related protein
136(1) WP_150241391.1 WP_150241391.1 albusnodin/ikarugamycin family macrolactam cyclase

137(1) WP_150241411.1 WP_150241411.1 hypothetical protein

137(1) WP_141786864.1 WP_141786864.1 hypothetical protein

138(1) WP_154346790.1 WP_154346790.1 hypothetical protein
138(1) WP_154346800.1 WP_154346800.1 hypothetical protein

139(1) WP_154346805.1 WP_154346805.1 hypothetical protein
139(1) WP_154346803.1 WP_154346803.1 hypothetical protein

140(1) WP_156186546.1 WP_156186546.1 AAA family ATPase
140(1) WP_144679710.1 WP_144679710.1 AAA family ATPase

141(1) WP_157581370.1 WP_157581370.1 hypothetical protein
141(1) WP_139278871.1 WP_139278871.1 hypothetical protein

142(1) WP_161111098.1 WP_161111098.1 NCS1 family transporter

142(1) WP_161111099.1 WP_161111099.1 NCS1 family nucleobase:cation symporter-1

143(1) WP_161111107.1 WP_161111107.1 helix-turn-helix domain-containing protein
143(1) WP_056154325.1 WP_056154325.1 MULTISPECIES: helix-turn-helix domain-containing protein

144(1) WP_056154341.1 WP_056154341.1 MULTISPECIES: TetR/AcrR family transcriptional regulator
144(1) WP_184443034.1 WP_184443034.1 TetR/AcrR family transcriptional regulator

145(1) WP_166527629.1 WP_166527629.1 MmcQ/YjbR family DNA-binding protein
145(1) WP_184443471.1 WP_184443471.1 MmcQ/YjbR family DNA-binding protein

146(1) WP_141786889.1 WP_141786889.1 NlpC/P60 family protein

146(1) WP_185275405.1 WP_185275405.1 hypothetical protein

147(1) WP_187437834.1 WP_187437834.1 metallophosphoesterase
147(1) WP_014373983.1 WP_014373983.1 metallophosphoesterase

148(1) WP_096288631.1 WP_096288631.1 MULTISPECIES: dihydroxy-acid dehydratase
148(1) WP_188947918.1 WP_188947918.1 dihydroxy-acid dehydratase

149(1) WP_189159261.1 WP_189159261.1 saccharopine dehydrogenase NADP-binding domain-containing protein
149(1) WP_186381198.1 WP_186381198.1 saccharopine dehydrogenase NADP-binding domain-containing protein

150(1) WP_048342054.1 WP_048342054.1 MULTISPECIES: CsbD family protein
150(1) WP_189159327.1 WP_189159327.1 CsbD family protein

151(1) WP_189481980.1 WP_189481980.1 three-Cys-motif partner protein TcmP
151(1) WP_189159275.1 WP_189159275.1 three-Cys-motif partner protein TcmP

152(1) WP_189481982.1 WP_189481982.1 phage Gp37/Gp68 family protein
152(1) WP_189159276.1 WP_189159276.1 phage Gp37/Gp68 family protein

153(1) WP_031023267.1 WP_031023267.1 MULTISPECIES: hypothetical protein
153(1) WP_189798731.1 WP_189798731.1 hypothetical protein

154(1) WP_006136587.1 WP_006136587.1 MULTISPECIES: VOC family protein
154(1) WP_189798732.1 WP_189798732.1 glyoxalase

155(1) WP_037817687.1 WP_037817687.1 iron ABC transporter permease
155(1) WP_189798735.1 WP_189798735.1 iron ABC transporter permease

156(1) WP_189798736.1 WP_189798736.1 thiamine ABC transporter substrate-binding protein
156(1) WP_189481987.1 WP_189481987.1 thiamine ABC transporter substrate-binding protein

157(1) WP_189798737.1 WP_189798737.1 23S rRNA (adenine(2503)-C(2))-methyltransferase RlmN
157(1) WP_189481984.1 WP_189481984.1 23S rRNA (adenine(2503)-C(2))-methyltransferase RlmN

158(1) WP_064065957.1 WP_064065957.1 cysteine desulfurase DndA
158(1) WP_199424251.1 WP_199424251.1 aminotransferase class V-fold PLP-dependent enzyme

159(1) WP_199424256.1 WP_199424256.1 glycerate kinase
159(1) WP_017534486.1 WP_017534486.1 glycerate kinase

160(1) WP_131885648.1 WP_131885648.1 helix-turn-helix domain-containing protein
160(1) WP_202423045.1 WP_202423045.1 metalloregulator ArsR/SmtB family transcription factor

161(1) WP_205653559.1 WP_205653559.1 pyruvate, water

dikinase regulatory protein
161(1) WP_166527635.1 WP_166527635.1 pyruvate, water dikinase regulatory protein

162(1) WP_208104786.1 WP_208104786.1 NAD(P)/FAD-dependent oxidoreductase
162(1) WP_166527638.1 WP_166527638.1 NAD(P)/FAD-dependent oxidoreductase

163(1) WP_210650320.1 WP_210650320.1 hypothetical protein
163(1) WP_210650318.1 WP_210650318.1 hypothetical protein

164(1) WP_150241376.1 WP_150241376.1 PspC domain-containing protein
164(1) WP_210650323.1 WP_210650323.1 PspC domain-containing protein

165(1) WP_014373961.1 WP_014373961.1 FtsW/RodA/SpoVE family cell cycle protein
165(1) WP_223078745.1 WP_223078745.1 rod shape-determining protein RodA

166(1) WP_014373960.1 WP_014373960.1 penicillin-binding protein 2
166(1) WP_225503928.1 WP_225503928.1 penicillin-binding transpeptidase domain-containing protein

167(1) WP_225503932.1 WP_225503932.1 putative glycolipid-binding domain-containing protein
167(1) WP_072740572.1 WP_072740572.1 MULTISPECIES: putative glycolipid-binding domain-containing protein

168(1) WP_134850827.1 WP_134850827.1 nucleotidyltransferase family protein
168(1) WP_229276234.1 WP_229276234.1 nucleotidyltransferase

domain-containing protein

169(1) WP_229863452.1 WP_229863452.1 LAETG motif-containing sortase-dependent surface protein
169(1) WP_006136588.1 WP_006136588.1 MULTISPECIES: LAETG motif-containing sortase-dependent surface protein

170(1) WP_207552803.1 WP_207552803.1 diol dehydratase reactivase
170(1) WP_231395951.1 WP_231395951.1 diol dehydratase reactivase ATPase-like domain-containing protein

171(1) WP_161111109.1 WP_161111109.1 cytochrome c oxidase subunit I
171(1) WP_233404941.1 WP_233404941.1 cytochrome c oxidase subunit I

172(1) WP_233515837.1 WP_233515837.1 Asp/Glu racemase
172(1) WP_110050906.1 WP_110050906.1 maleate cis-trans isomerase

173(1) WP_069740785.1 WP_069740785.1 helix-turn-helix transcriptional regulator
173(1) WP_234432663.1 WP_234432663.1 XRE family transcriptional regulator

174(1) WP_147918749.1 WP_147918749.1 phosphate ABC transporter permease PstA
174(1) WP_235912470.1 WP_235912470.1 phosphate ABC transporter permease subunit PstC

175(1) WP_234367440.1 WP_234367440.1 MULTISPECIES: hypothetical protein
175(1) WP_242435778.1 WP_242435778.1 hypothetical protein

176(1) WP_092727721.1 WP_092727721.1 phage scaffolding protein
176(1) WP_243109666.1 WP_243109666.1 phage scaffolding protein

177(1) WP_117946277.1 WP_117946277.1 MULTISPECIES: HAD-IA family hydrolase
177(1) WP_243718308.1 WP_243718308.1 HAD family hydrolase

178(1) WP_245852821.1 WP_245852821.1 hypothetical protein
178(1) WP_097195734.1 WP_097195734.1 HhH-GPD-type base excision DNA repair protein

179(1) WP_093738711.1 WP_093738711.1 MULTISPECIES: ROK family protein
179(1) WP_245899381.1 WP_245899381.1 ROK family protein

180(1) WP_246861933.1 WP_246861933.1 glycerophosphodiester phosphodiesterase family protein
180(1) WP_234538989.1 WP_234538989.1 glycerophosphodiester phosphodiesterase

181(1) WP_246906739.1 WP_246906739.1 TetR/AcrR family transcriptional regulator
181(1) WP_150241379.1 WP_150241379.1 TetR/AcrR family transcriptional regulator

182(1) WP_175337886.1 WP_175337886.1 MULTISPECIES: heavy metal translocating P-type ATPase
182(1) WP_249776717.1 WP_249776717.1 MULTISPECIES: heavy metal translocating P-type ATPase

183(1) WP_231969138.1 WP_231969138.1 MULTISPECIES: glycoside hydrolase

183(1) WP_250358779.1 WP_250358779.1 hypothetical protein

184(1) WP_132045625.1 WP_132045625.1 hypothetical protein
184(1) WP_250358786.1 WP_250358786.1 hypothetical protein

185(1) WP_255847326.1 WP_255847326.1 hypothetical protein
185(1) WP_010965227.1 WP_010965227.1 MULTISPECIES: hypothetical protein

186(1) WP_010965201.1 WP_010965201.1 MULTISPECIES: HNH endonuclease signature motif containing protein
186(1) WP_255847338.1 WP_255847338.1 HNH endonuclease signature motif containing protein

187(1) WP_256843044.1 WP_256843044.1 VWA domain-containing protein
187(1) WP_213011324.1 WP_213011324.1 VWA domain-containing protein

188(1) WP_131166525.1 WP_131166525.1 restriction endonuclease subunit S
188(1) WP_259308330.1 WP_259308330.1 restriction endonuclease subunit S

189(1) WP_259308338.1 WP_259308338.1 transferase
189(1) WP_246906730.1 WP_246906730.1 transferase

190(1) WP_082580592.1 WP_082580592.1 MFS transporter
190(1) WP_259308345.1 WP_259308345.1 MFS transporter

191(1) WP_259308348.1 WP_259308348.1 hypothetical protein
191(1) WP_259308322.1 WP_259308322.1 hypothetical protein

192(1) WP_246906733.1 WP_246906733.1 fumarate reductase/succinate dehydrogenase flavoprotein subunit
192(1) WP_259808647.1 WP_259808647.1 fumarate reductase/succinate dehydrogenase flavoprotein subunit

193(1) WP_259808648.1 WP_259808648.1 succinate dehydrogenase/fumarate reductase iron-sulfur subunit
193(1) WP_246906734.1 WP_246906734.1 succinate dehydrogenase/fumarate reductase iron-sulfur subunit

194(1) WP_259808653.1 WP_259808653.1 SRPBCC family protein
194(1) WP_166527632.1 WP_166527632.1 hypothetical protein

195(1) WP_071659272.1 WP_071659272.1 MULTISPECIES: M56 family metallopeptidase
195(1) WP_261557226.1 WP_261557226.1 M56 family metallopeptidase

196(1) WP_243109665.1 WP_243109665.1 ATP-binding protein
196(1) WP_264318381.1 WP_264318381.1 ATP-binding protein

197(1) WP_266515842.1 WP_266515842.1 hypothetical protein
197(1) WP_057608700.1 WP_057608700.1 hypothetical protein

198(1) WP_266518750.1 WP_266518750.1 RRQRL motif-containing zinc-binding protein
198(1) WP_263023578.1 WP_263023578.1 RRQRL motif-containing zinc-binding protein

199(2) WP_266761003.1 WP_266761003.1 MULTISPECIES: nitrate- and nitrite sensing domain-containing protein

200(2) WP_266761009.1 WP_266761009.1 MULTISPECIES: hypothetical protein

201(2) WP_266761013.1 WP_266761013.1 MULTISPECIES: hypothetical protein

202(2) WP_266761015.1 WP_266761015.1 MULTISPECIES: transcriptional regulator

203(2) WP_266761017.1 WP_266761017.1 MULTISPECIES: DEAD/DEAH box helicase

204(2) WP_266761032.1 WP_266761032.1 MULTISPECIES: hypothetical protein

205(2) WP_266761038.1 WP_266761038.1 MULTISPECIES: hypothetical protein

206(2) WP_266761040.1 WP_266761040.1 MULTISPECIES: hypothetical protein

207(1) WP_222869875.1 WP_222869875.1 BTAD domain-containing putative transcriptional regulator
207(1) WP_266811461.1 WP_266811461.1 BTAD domain-containing putative transcriptional regulator

208(1) WP_266518754.1 WP_266518754.1 hypothetical protein
208(1) WP_266811494.1 WP_266811494.1 hypothetical protein

209(1) WP_266811496.1 WP_266811496.1 hypothetical protein
209(1) WP_266518753.1 WP_266518753.1 hypothetical protein

210(1) WP_266761011.1 WP_266761011.1 hypothetical protein
210(1) WP_266848394.1 WP_266848394.1 hypothetical protein

211(1) WP_269689497.1 WP_269689497.1 HAD family phosphatase
211(1) WP_263023689.1 WP_263023689.1 HAD family phosphatase

212(1) WP_084011283.1 WP_084011283.1 UvrD-helicase domain-containing protein
212(1) WP_269689501.1 WP_269689501.1 ATP-dependent DNA helicase

213(1) WP_269689500.1 WP_269689500.1 DUF3578 domain-containing protein
213(1) WP_269689502.1 WP_269689502.1 DUF3883 domain-containing protein

214(1) WP_093738749.1 WP_093738749.1 MULTISPECIES: DNA polymerase III subunit delta'
214(1) WP_278177377.1 WP_278177377.1 DNA polymerase III subunit gamma and tau

215(1) WP_278177379.1 WP_278177379.1 hypothetical protein
215(1) WP_269689507.1 WP_269689507.1 hypothetical protein

216(1) WP_269689506.1 WP_269689506.1 hypothetical protein
216(1) WP_278177380.1 WP_278177380.1 hypothetical protein

217(1) WP_278177394.1 WP_278177394.1 purine-nucleoside phosphorylase
217(1) WP_091070383.1 WP_091070383.1 MULTISPECIES: purine-nucleoside phosphorylase

218(1) WP_269689496.1 WP_269689496.1 tRNA adenosine deaminase-associated protein
218(1) WP_278177399.1 WP_278177399.1 tRNA adenosine deaminase-associated protein

219(1) WP_269694696.1 WP_269694696.1 hypothetical protein
219(1) WP_278177401.1 WP_278177401.1 hypothetical protein

220(1) WP_269689495.1 WP_269689495.1 M23 family metallopeptidase
220(1) WP_278177403.1 WP_278177403.1 M23 family metallopeptidase

221(1) WP_156741178.1 WP_156741178.1 hypothetical protein
221(1) WP_280911782.1 WP_280911782.1 hypothetical protein

222(1) WP_281292207.1 WP_281292207.1 alpha/beta hydrolase
222(1) WP_093738745.1 WP_093738745.1 MULTISPECIES: alpha/beta hydrolase

223(1) WP_283319017.1 WP_283319017.1 hypothetical protein
223(1) WP_147794795.1 WP_147794795.1 hypothetical protein

224(1) WP_283319032.1 WP_283319032.1 hypothetical protein
224(1) WP_147794787.1 WP_147794787.1 hypothetical protein

225(1) WP_056154352.1 WP_056154352.1 MULTISPECIES: NAD-dependent deacylase
225(1) WP_283663878.1 WP_283663878.1 Sir2 family NAD-dependent protein deacetylase

226(1) WP_283666125.1 WP_283666125.1 replication initiator protein
226(1) WP_243718312.1 WP_243718312.1 replication initiation protein

227(1) WP_307861938.1 WP_307861938.1 aminotransferase class

V-fold PLP-dependent enzyme
227(1) WP_154347002.1 WP_154347002.1 aminotransferase class V-fold PLP-dependent enzyme

228(1) WP_110050904.1 WP_110050904.1 D-2-hydroxyacid dehydrogenase
228(1) WP_308739406.1 WP_308739406.1 D-2-hydroxyacid dehydrogenase

229(1) WP_259815461.1 WP_259815461.1 hypothetical protein
229(1) WP_308739413.1 WP_308739413.1 restriction endonuclease subunit S

230(1) WP_150241414.1 WP_150241414.1 ABC transporter permease
230(1) WP_308739420.1 WP_308739420.1 ABC transporter permease

231(1) WP_070706184.1 WP_070706184.1 MULTISPECIES: AAA family ATPase
231(1) WP_308739428.1 WP_308739428.1 AAA family ATPase

232(1) WP_312004117.1 WP_312004117.1 pentapeptide repeat-containing protein
232(1) WP_250358791.1 WP_250358791.1 pentapeptide repeat-containing protein

233(1) WP_092727719.1 WP_092727719.1 major capsid protein
233(1) WP_312004121.1 WP_312004121.1 major capsid protein

234(1) WP_315912318.1 WP_315912318.1 hypothetical protein
234(1) WP_243699660.1 WP_243699660.1 hydrolase

235(1) WP_259815463.1 WP_259815463.1 hypothetical protein,

partial
235(1) WP_318603642.1 WP_318603642.1 hypothetical protein

236(1) WP_320965079.1 WP_320965079.1 tryptophan-rich sensory protein
236(1) WP_096288609.1 WP_096288609.1 tryptophan-rich sensory protein

237(1) WP_320965092.1 WP_320965092.1 hypothetical protein
237(1) WP_236641098.1 WP_236641098.1 hypothetical protein

238(1) WP_320965093.1 WP_320965093.1 glucose-1-phosphate thymidylyltransferase RfbA
238(1) WP_128470516.1 WP_128470516.1 glucose-1-phosphate thymidylyltransferase RfbA

239(1) WP_320965099.1 WP_320965099.1 DegT/DnrJ/EryC1/StrS family aminotransferase
239(1) WP_320965101.1 WP_320965101.1 DegT/DnrJ/EryC1/StrS family aminotransferase

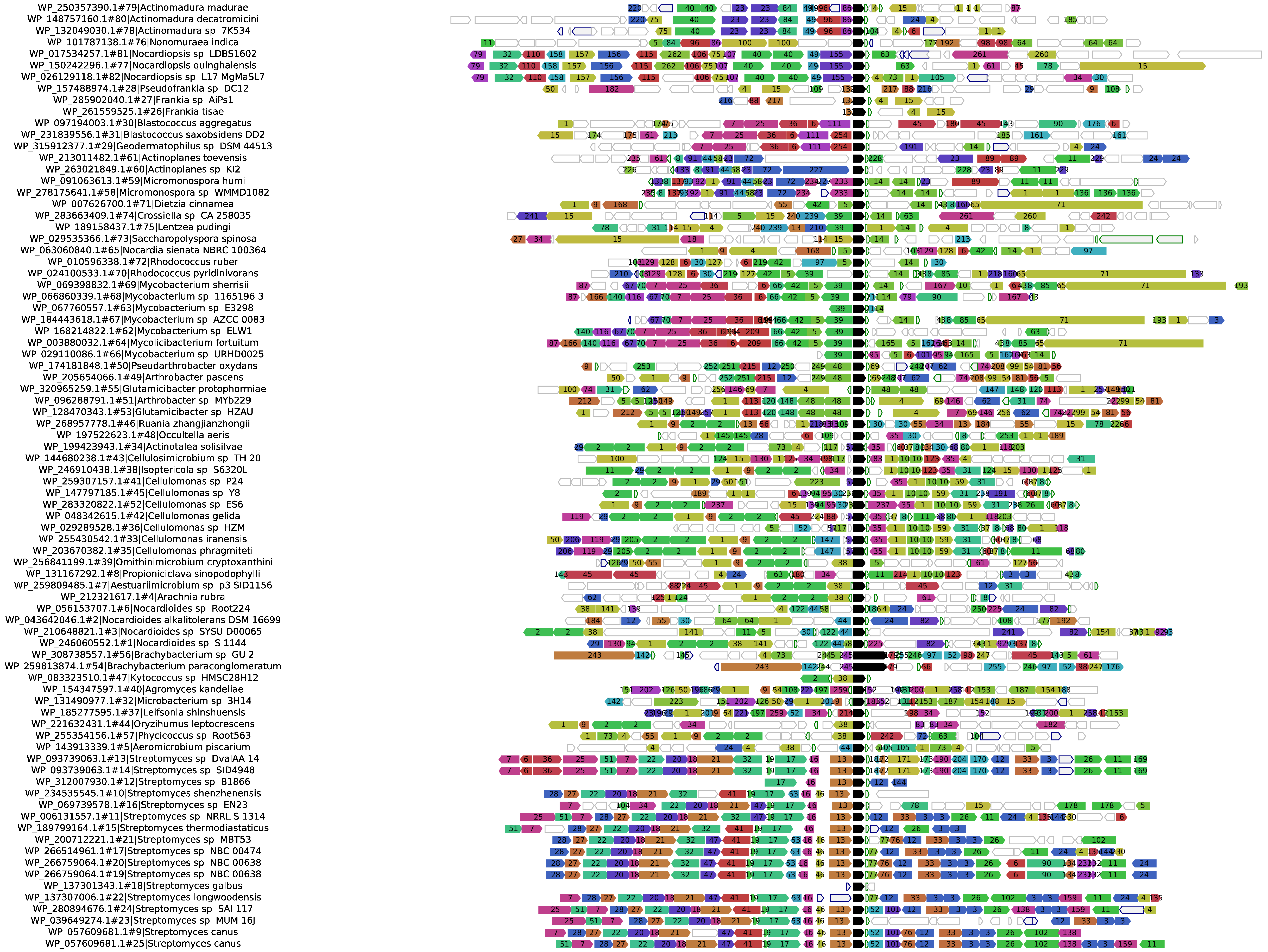
WP_250357390.1#79|Actinomadura madurae
WP_148757160.1#80|Actinomadura decatromicini
WP_132049030.1#78|Actinomadura sp 7K534
WP_101787138.1#76|Nonomuraea indica
WP_017534257.1#81|Nocardiopsis sp LDBS1602
WP_150242296.1#77|Nocardiopsis quinghaiensis
WP_026129118.1#82|Nocardiopsis sp L17 MgMaSL7
WP_157488974.1#28|Pseudofrankia sp DC12
WP_285902040.1#27|Frankia sp AiPs1
WP_261559525.1#26|Frankia tisae
WP_097194003.1#30|Blastococcus aggregatus
WP_231839556.1#31|Blastococcus saxobsidens DD2
WP_315912377.1#29|Geodermatophilus sp DSM 44513
WP_213011482.1#61|Actinoplanes toevensis
WP_263021849.1#60|Actinoplanes sp KI2
WP_091063613.1#59|Micromonospora humi
WP_278175641.1#58|Micromonospora sp WMMD1082
WP_007626700.1#71|Dietzia cinnamea
WP_283663409.1#74|Crossiella sp CA 258035
WP_189158437.1#75|Lentzea pudingi
WP_029535366.1#73|Saccharopolyspora spinosa
WP_063060840.1#65|Nocardia sienata NBRC 100364
WP_010596338.1#72|Rhodococcus ruber
WP_024100533.1#70|Rhodococcus pyridinivorans
WP_069398832.1#69|Mycobacterium sherrisii
WP_066860339.1#68|Mycobacterium sp 1165196 3
WP_067760552.1#63|Mycobacterium sp E3298
WP_184443618.1#67|Mycobacterium sp AZCC 0083
WP_168214822.1#62|Mycobacterium sp ELW1
WP_003880032.1#64|Mycolicibacterium fortuitum
WP_029110086.1#66|Mycobacterium sp URHD0025
WP_174181848.1#50|Pseudarthrobacter oxydans
WP_205654066.1#49|Arthrobacter pascens
WP_320965259.1#55|Glutamicibacter protophormiae
WP_096288791.1#51|Arthrobacter sp MYb229
WP_128470343.1#53|Glutamicibacter sp HZAU
WP_268957778.1#46|Ruania zhangjianzhongii
WP_197522623.1#48|Occultella aeris
WP_199423943.1#34|Actinotalea solisilvae
WP_144680238.1#43|Cellulosimicrobium sp TH 20
WP_246910438.1#38|Isoptericola sp S6320L
WP_259307157.1#41|Cellulomonas sp P24
WP_147797185.1#45|Cellulomonas sp Y8
WP_283320822.1#52|Cellulomonas sp ES6
WP_048342615.1#42|Cellulomonas gelida
WP_029289528.1#36|Cellulomonas sp HZM
WP_255430542.1#33|Cellulomonas iranensis
WP_203670382.1#35|Cellulomonas phragmiteti
WP_256841199.1#39|Ornithinimicrobium cryptoxanthini
WP_131167292.1#8|Propioniciclava sinopodophylli
WP_259809485.1#7|Aestuariimicrobium sp p3 SID1156
WP_212321617.1#4|Arachnia rubra
WP_056153707.1#6|Nocardioides sp Root224
WP_043642046.1#2|Nocardioides alkalitolerans DSM 16699
WP_210648821.1#3|Nocardioides sp SYSU D00065
WP_246060552.1#1|Nocardioides sp S 1144
WP_308738557.1#56|Brachybacterium sp GU 2
WP_259813874.1#54|Brachybacterium paraconglomeratum
WP_083323510.1#47|Kytococcus sp HMSC28H12
WP_154347597.1#40|Agromyces kandeliae
WP_131490977.1#32|Microbacterium sp 3H14
WP_185277595.1#37|Leifsonia shinshuensis
WP_221632431.1#44|Oryzihumus leptocrescens
WP_255354156.1#57|Phycicoccus sp Root563
WP_143913339.1#5|Aeromicrobium piscarium
WP_093739063.1#13|Streptomyces sp DvalAA 14
WP_093739063.1#14|Streptomyces sp SID4948
WP_312007930.1#12|Streptomyces sp B1866
WP_234535545.1#10|Streptomyces shenzhenensis
WP_069739578.1#16|Streptomyces sp EN23
WP_006131557.1#11|Streptomyces sp NRRL S 1314
WP_189799164.1#15|Streptomyces thermodiastaticus
WP_200712221.1#21|Streptomyces sp MBT53
WP_266514961.1#17|Streptomyces sp NBC 00474
WP_266759064.1#20|Streptomyces sp NBC 00638
WP_266759064.1#19|Streptomyces sp NBC 00638
WP_137301343.1#18|Streptomyces galbus
WP_137307006.1#22|Streptomyces longwoodensis
WP_280894676.1#24|Streptomyces sp SAI 117
WP_039649274.1#23|Streptomyces sp MUM 16J
WP_057609681.1#9|Streptomyces canus
WP_057609681.1#25|Streptomyces canus

1(1)   WP_137292551.1 WP_137292551.1 MULTISPECIES: energy-dependent translational throttle protein EttA
1(1)   WP_063060726.1 WP_063060726.1 energy-dependent translational throttle protein EttA
1(1)   WP_278175632.1 WP_278175632.1 ABC transporter ATP-binding protein
1(1)   WP_245852229.1 WP_245852229.1 ABC transporter ATP-binding protein
1(1)   WP_324613570.1 WP_324613570.1 ABC transporter ATP-binding protein
1(1)   WP_188950306.1 WP_188950306.1 ABC-F family ATP-binding cassette domain-containing protein
1(1)   WP_034654058.1 WP_034654058.1 MULTISPECIES: ATP-binding cassette domain-containing protein
1(1)   WP_189158447.1 WP_189158447.1 ATP-binding cassette domain-containing protein
1(1)   WP_091063588.1 WP_091063588.1 ABC transporter ATP-binding protein
1(1)   WP_189158438.1 WP_189158438.1 ABC-F family ATP-binding cassette domain-containing protein
1(1)   WP_046528008.1 WP_046528008.1 MULTISPECIES: ABC transporter ATP-binding protein
1(1)   WP_210648831.1 WP_210648831.1 ATP-binding cassette domain-containing protein
1(1)   WP_063060740.1 WP_063060740.1 ATP-binding cassette domain-containing protein
1(1)   WP_232326894.1 WP_232326894.1 ABC transporter ATP-binding protein
1(1)   WP_324613568.1 WP_324613568.1 energy-dependent translational throttle protein EttA
1(1)   WP_106266538.1 WP_106266538.1 MULTISPECIES: energy-dependent translational throttle protein EttA
1(1)   WP_147916394.1 WP_147916394.1 energy-dependent translational throttle protein EttA
1(1)   WP_199423933.1 WP_199423933.1 energy-dependent translational throttle protein EttA
1(1)   WP_150242304.1 WP_150242304.1 ATP-binding cassette domain-containing protein
1(1)   WP_143913334.1 WP_143913334.1 ABC transporter ATP-binding protein
1(1)   WP_137292536.1 WP_137292536.1 MULTISPECIES: ABC transporter ATP-binding protein

1(1)   WP_278175649.1 WP_278175649.1 ABC transporter ATP-binding protein
1(1)   WP_096288781.1 WP_096288781.1 MULTISPECIES: ABC-F family ATP-binding cassette domain-containing protein
1(1)   WP_255277158.1 WP_255277158.1 hypothetical protein
1(1)   WP_231955480.1 WP_231955480.1 ABC transporter ATP-binding protein
1(1)   WP_246910442.1 WP_246910442.1 ATP-binding cassette domain-containing protein
1(1)   WP_084540989.1 WP_084540989.1 ABC transporter ATP-binding protein
1(1)   WP_144680724.1 WP_144680724.1 ABC transporter ATP-binding protein
1(1)   WP_147796144.1 WP_147796144.1 ATP-binding cassette domain-containing protein
1(1)   WP_046528014.1 WP_046528014.1 MULTISPECIES: energy-dependent translational throttle protein EttA
1(1)   WP_082581092.1 WP_082581092.1 ATP-binding cassette domain-containing protein
1(1)   WP_156742050.1 WP_156742050.1 ATP-binding cassette domain-containing protein
1(1)   WP_205654057.1 WP_205654057.1 energy-dependent translational throttle protein EttA
1(1)   WP_156742070.1 WP_156742070.1 ABC transporter ATP-binding protein
1(1)   WP_110047914.1 WP_110047914.1 ABC transporter ATP-binding protein
1(1)   WP_212321609.1 WP_212321609.1 energy-dependent translational throttle protein EttA
1(1)   WP_147796152.1 WP_147796152.1 ABC transporter ATP-binding protein
1(1)   WP_106266576.1 WP_106266576.1 MULTISPECIES: ATP-binding cassette domain-containing protein
1(1)   WP_141368714.1 WP_141368714.1 ABC-F family ATP-binding cassette domain-containing protein
1(1)   WP_278175631.1 WP_278175631.1 ABC transporter ATP-binding protein
1(1)   WP_259307160.1 WP_259307160.1 ATP-binding cassette domain-containing protein
1(1)   WP_259809480.1 WP_259809480.1 energy-dependent translational throttle protein EttA
1(1)   WP_029289549.1 WP_029289549.1 ABC-F family

ATP-binding cassette domain-containing protein
1(1)   WP_056917802.1 WP_056917802.1 energy-dependent translational throttle protein EttA
1(1)   WP_128470332.1 WP_128470332.1 ABC transporter ATP-binding protein
1(1)   WP_239068968.1 WP_239068968.1 ABC transporter ATP-binding protein
1(1)   WP_132049041.1 WP_132049041.1 ABC transporter ATP-binding protein
1(1)   WP_147916385.1 WP_147916385.1 ATP-binding cassette domain-containing protein
1(1)   WP_246910454.1 WP_246910454.1 ABC transporter ATP-binding protein
1(1)   WP_184443607.1 WP_184443607.1 ABC transporter ATP-binding protein
1(1)   WP_203670390.1 WP_203670390.1 energy-dependent translational throttle protein EttA
1(1)   WP_212321602.1 WP_212321602.1 ABC transporter ATP-binding protein
1(1)   WP_307844839.1 WP_307844839.1 ATP-binding cassette domain-containing protein
1(1)   WP_128470340.1 WP_128470340.1 ABC-F family ATP-binding cassette domain-containing protein
1(1)   WP_147796153.1 WP_147796153.1 MULTISPECIES: ATP-binding cassette domain-containing protein
1(1)   WP_185277604.1 WP_185277604.1 ATP-binding cassette domain-containing protein
1(1)   WP_185277586.1 WP_185277586.1 energy-dependent translational throttle protein EttA
1(1)   WP_252619911.1 WP_252619911.1 energy-dependent translational throttle protein EttA
1(1)   WP_019288999.1 WP_019288999.1 MULTISPECIES: ATP-binding cassette domain-containing protein
1(1)   WP_051681790.1 WP_051681790.1 ABC transporter ATP-binding protein
1(1)   WP_283320808.1 WP_283320808.1 energy-dependent translational throttle protein EttA
1(1)   WP_132049043.1 WP_132049043.1 ABC transporter ATP-binding protein
1(1)   WP_070721217.1 WP_070721217.1 MULTISPECIES: energy-dependent translational throttle protein EttA
1(1)   WP_283320824.1 WP_283320824.1 ATP-binding cassette

domain-containing protein
1(1)   WP_141368726.1 WP_141368726.1 energy-dependent translational throttle protein EttA
1(1)   WP_047523319.1 WP_047523319.1 MULTISPECIES: energy-dependent translational throttle protein EttA
1(1)   WP_131167295.1 WP_131167295.1 ATP-binding cassette domain-containing protein
1(1)   WP_255268938.1 WP_255268938.1 ATP-binding cassette domain-containing protein
1(1)   WP_255275941.1 WP_255275941.1 ATP-binding cassette domain-containing protein
1(1)   WP_141787616.1 WP_141787616.1 energy-dependent translational throttle protein EttA
1(1)   WP_259307151.1 WP_259307151.1 energy-dependent translational throttle protein EttA

2(1)   WP_259809482.1 WP_259809482.1 GTPase
2(1)   WP_199423935.1 WP_199423935.1 GTPase
2(1)   WP_252619913.1 WP_252619913.1 GTPase
2(1)   WP_259307149.1 WP_259307149.1 GTPase
2(1)   WP_306254375.1 WP_306254375.1 dynamin family protein
2(1)   WP_210648810.1 WP_210648810.1 YfjP family GTPase
2(1)   WP_070320852.1 WP_070320852.1 GTPase domain-containing protein
2(1)   WP_283320811.1 WP_283320811.1 dynamin family protein
2(1)   WP_187366827.1 WP_187366827.1 GTPase
2(1)   WP_246092051.1 WP_246092051.1 50S ribosome-binding GTPase
2(1)   WP_229747450.1 WP_229747450.1 dynamin family protein
2(1)   WP_147916391.1 WP_147916391.1 GTPase domain-containing protein
2(1)   WP_262347582.1 WP_262347582.1 dynamin family protein
2(1)   WP_199423931.1 WP_199423931.1 GTPase
2(1)   WP_212321613.1 WP_212321613.1 ABC transporter
2(1)   WP_237769746.1 WP_237769746.1 ABC transporter, partial
2(1)   WP_246855037.1 WP_246855037.1 dynamin family protein
2(1)   WP_283320810.1 WP_283320810.1 50S ribosome-binding GTPase
2(1)   WP_203670388.1 WP_203670388.1 GTPase
2(1)   WP_147916392.1 WP_147916392.1 GTP-binding protein

2(1)   WP_307415669.1 WP_307415669.1 dynamin family protein
2(1)   WP_246910426.1 WP_246910426.1 GTPase
2(1)   WP_212321611.1 WP_212321611.1 50S ribosome-binding GTPase
2(1)   WP_070319959.1 WP_070319959.1 GTPase
2(1)   WP_246910418.1 WP_246910418.1 dynamin family protein
2(1)   WP_246861436.1 WP_246861436.1 dynamin family protein
2(1)   WP_229747451.1 WP_229747451.1 GTPase domain-containing protein
2(1)   WP_056917804.1 WP_056917804.1 GTPase
2(1)   WP_246910416.1 WP_246910416.1 GTPase
2(1)   WP_141368730.1 WP_141368730.1 GTPase
2(1)   WP_141787614.1 WP_141787614.1 ABC transporter
2(1)   WP_203670392.1 WP_203670392.1 GTPase
2(1)   WP_141368724.1 WP_141368724.1 MULTISPECIES: GTPase
2(1)   WP_203670391.1 WP_203670391.1 dynamin family protein
2(1)   WP_070320851.1 WP_070320851.1 MULTISPECIES: GTPase
2(1)   WP_199423988.1 WP_199423988.1 dynamin family protein
2(1)   WP_246910429.1 WP_246910429.1 GTPase
2(1)   WP_056917805.1 WP_056917805.1 GTPase domain-containing protein
2(1)   WP_203670387.1 WP_203670387.1 GTPase domain-containing protein
2(1)   WP_259307148.1 WP_259307148.1 dynamin family protein
2(1)   WP_259809483.1 WP_259809483.1 dynamin family protein
2(1)   WP_199423932.1 WP_199423932.1 dynamin family protein

3(1)   WP_266383313.1 WP_266383313.1 MULTISPECIES: carbohydrate ABC transporter permease
3(1)   WP_039649281.1 WP_039649281.1 MULTISPECIES: sugar ABC transporter permease
3(1)   WP_057609687.1 WP_057609687.1 MULTISPECIES: sugar ABC transporter permease
3(1)   WP_280851330.1 WP_280851330.1 sugar ABC transporter permease
3(1)   WP_266383310.1 WP_266383310.1 sugar ABC transporter permease
3(1)   WP_189799168.1 WP_189799168.1 sugar ABC transporter permease
3(1)   WP_131167302.1 WP_131167302.1 MULTISPECIES: sugar ABC transporter permease

3(1)   WP_131167301.1 WP_131167301.1 carbohydrate ABC transporter permease
3(1)   WP_006131561.1 WP_006131561.1 MULTISPECIES: carbohydrate ABC transporter permease
3(1)   WP_202451647.1 WP_202451647.1 sugar ABC transporter permease
3(1)   WP_189799167.1 WP_189799167.1 carbohydrate ABC transporter permease
3(1)   WP_093739047.1 WP_093739047.1 sugar ABC transporter permease
3(1)   WP_255506995.1 WP_255506995.1 ABC transporter permease
3(1)   WP_201049334.1 WP_201049334.1 carbohydrate ABC transporter permease
3(1)   WP_137307010.1 WP_137307010.1 MULTISPECIES: sugar ABC transporter permease
3(1)   WP_266514969.1 WP_266514969.1 MULTISPECIES: sugar ABC transporter permease
3(1)   WP_137301349.1 WP_137301349.1 MULTISPECIES: carbohydrate ABC transporter permease
3(1)   WP_280853442.1 WP_280853442.1 carbohydrate ABC transporter permease
3(2)   WP_266759073.1 WP_266759073.1 MULTISPECIES: sugar ABC transporter permease
3(1)   WP_031017392.1 WP_031017392.1 MULTISPECIES: sugar ABC transporter permease
3(1)   WP_201049333.1 WP_201049333.1 sugar ABC transporter permease
3(1)   WP_266383325.1 WP_266383325.1 carbohydrate ABC transporter permease
3(1)   WP_280897757.1 WP_280897757.1 sugar ABC transporter permease
3(1)   WP_280851329.1 WP_280851329.1 carbohydrate ABC transporter permease
3(1)   WP_266514971.1 WP_266514971.1 MULTISPECIES: carbohydrate ABC transporter permease
3(1)   WP_326780452.1 WP_326780452.1 carbohydrate ABC transporter permease
3(1)   WP_039649283.1 WP_039649283.1 MULTISPECIES: carbohydrate ABC transporter permease
3(2)   WP_266759074.1 WP_266759074.1 MULTISPECIES: carbohydrate ABC transporter permease

3(1) WP_199873685.1 WP_199873685.1 MULTISPECIES: carbohydrate ABC transporter permease
3(1) WP_329243486.1 WP_329243486.1 sugar ABC transporter permease
3(1) WP_326780453.1 WP_326780453.1 sugar ABC transporter permease

4(1) WP_246065147.1 WP_246065147.1 response regulator transcription factor
4(1) WP_056917799.1 WP_056917799.1 response regulator transcription factor
4(1) WP_144680246.1 WP_144680246.1 response regulator transcription factor
4(1) WP_067236204.1 WP_067236204.1 MULTISPECIES: response regulator transcription factor
4(1) WP_031017388.1 WP_031017388.1 response regulator transcription factor
4(1) WP_166527892.1 WP_166527892.1 response regulator transcription factor
4(1) WP_189158440.1 WP_189158440.1 helix-turn-helix transcriptional regulator
4(1) WP_045877958.1 WP_045877958.1 response regulator transcription factor
4(1) WP_251746087.1 WP_251746087.1 response regulator transcription factor
4(1) WP_320965258.1 WP_320965258.1 LuxR C-terminal-related transcriptional regulator
4(1) WP_131167285.1 WP_131167285.1 response regulator transcription factor
4(1) WP_110047912.1 WP_110047912.1 response regulator transcription factor
4(1) WP_161567472.1 WP_161567472.1 LuxR family transcriptional regulator
4(1) WP_187437667.1 WP_187437667.1 response regulator transcription factor
4(1) WP_056153723.1 WP_056153723.1 MULTISPECIES: response regulator transcription factor
4(1) WP_132049035.1 WP_132049035.1 response regulator transcription factor
4(1) WP_266514979.1 WP_266514979.1 response regulator transcription factor

4(1)   WP_056154174.1 WP_056154174.1 MULTISPECIES: response regulator transcription factor
4(1)   WP_136448721.1 WP_136448721.1 MULTISPECIES: response regulator transcription factor
4(1)   WP_143913345.1 WP_143913345.1 MtrAB system response regulator MtrA
4(1)   WP_063060727.1 WP_063060727.1 AAA family ATPase
4(1)   WP_143913332.1 WP_143913332.1 response regulator transcription factor
4(1)   WP_308738551.1 WP_308738551.1 response regulator transcription factor
4(1)   WP_181148254.1 WP_181148254.1 MULTISPECIES: helix-turn-helix transcriptional regulator
4(1)   WP_255268934.1 WP_255268934.1 response regulator transcription factor
4(1)   WP_189158432.1 WP_189158432.1 helix-turn-helix transcriptional regulator
4(1)   WP_199423938.1 WP_199423938.1 response regulator transcription factor
4(1)   WP_280898546.1 WP_280898546.1 response regulator transcription factor

5(1)   WP_038991000.1 WP_038991000.1 MULTISPECIES: beta-ketoacyl-ACP reductase
5(1)   WP_029110083.1 WP_029110083.1 SDR family oxidoreductase
5(1)   WP_029289520.1 WP_029289520.1 SDR family oxidoreductase
5(1)   WP_184443620.1 WP_184443620.1 mycolate reductase
5(1)   WP_029110077.1 WP_029110077.1 SDR family oxidoreductase
5(1)   WP_105552070.1 WP_105552070.1 MULTISPECIES: SDR family oxidoreductase
5(1)   WP_143913329.1 WP_143913329.1 SDR family NAD(P)-dependent oxidoreductase
5(1)   WP_143913337.1 WP_143913337.1 SDR family NAD(P)-dependent oxidoreductase
5(1)   WP_070721251.1 WP_070721251.1 MULTISPECIES: mycolate reductase
5(1)   WP_069738596.1 WP_069738596.1 MULTISPECIES: SDR family oxidoreductase

5(1)   WP_210648815.1 WP_210648815.1 SDR family oxidoreductase
5(1)   WP_066860333.1 WP_066860333.1 MULTISPECIES: mycolate reductase
5(1)   WP_308738555.1 WP_308738555.1 SDR family oxidoreductase
5(1)   WP_128470335.1 WP_128470335.1 SDR family oxidoreductase
5(1)   WP_006554373.1 WP_006554373.1 MULTISPECIES: mycolate reductase
5(1)   WP_252619917.1 WP_252619917.1 3-oxoacyl-ACP reductase FabG
5(1)   WP_054602803.1 WP_054602803.1 MULTISPECIES: SDR family oxidoreductase
5(1)   WP_010596339.1 WP_010596339.1 MULTISPECIES: mycolate reductase
5(1)   WP_101787144.1 WP_101787144.1 SDR family NAD(P)-dependent oxidoreductase
5(1)   WP_205654077.1 WP_205654077.1 oxidoreductase
5(1)   WP_308738567.1 WP_308738567.1 SDR family oxidoreductase
5(1)   WP_149378846.1 WP_149378846.1 mycolate reductase
5(1)   WP_283663414.1 WP_283663414.1 SDR family oxidoreductase
5(1)   WP_063060730.1 WP_063060730.1 mycolate reductase
5(1)   WP_003880030.1 WP_003880030.1 mycolate reductase
5(1)   WP_181148253.1 WP_181148253.1 MULTISPECIES: beta-ketoacyl-ACP reductase
5(1)   WP_069398834.1 WP_069398834.1 mycolate reductase

6(1)   WP_014375111.1 WP_014375111.1 TetR/AcrR family transcriptional regulator
6(1)   WP_102030791.1 WP_102030791.1 MULTISPECIES: TetR/AcrR family transcriptional regulator
6(1)   WP_029110082.1 WP_029110082.1 TetR/AcrR family transcriptional regulator
6(1)   WP_132049037.1 WP_132049037.1 TetR/AcrR family transcriptional regulator
6(1)   WP_069398837.1 WP_069398837.1 TetR/AcrR family transcriptional regulator
6(1)   WP_286199267.1 WP_286199267.1 TetR/AcrR family

transcriptional regulator
6(2)   WP_266759078.1 WP_266759078.1 MULTISPECIES: TetR/AcrR family transcriptional regulator
6(1)   WP_069398827.1 WP_069398827.1 TetR/AcrR family transcriptional regulator
6(1)   WP_082972524.1 WP_082972524.1 MULTISPECIES: TetR/AcrR family transcriptional regulator
6(2)   WP_093739082.1 WP_093739082.1 MULTISPECIES: TetR/AcrR family transcriptional regulator
6(1)   WP_006554379.1 WP_006554379.1 MULTISPECIES: TetR family transcriptional regulator
6(1)   WP_054602799.1 WP_054602799.1 TetR/AcrR family transcriptional regulator
6(1)   WP_166527905.1 WP_166527905.1 TetR/AcrR family transcriptional regulator
6(1)   WP_147796150.1 WP_147796150.1 TetR/AcrR family transcriptional regulator
6(1)   WP_176522774.1 WP_176522774.1 TetR/AcrR family transcriptional regulator
6(1)   WP_040271738.1 WP_040271738.1 MULTISPECIES: TetR family transcriptional regulator
6(1)   WP_184443625.1 WP_184443625.1 TetR/AcrR family transcriptional regulator
6(1)   WP_097193157.1 WP_097193157.1 TetR/AcrR family transcriptional regulator
6(1)   WP_245665509.1 WP_245665509.1 TetR family transcriptional regulator
6(1)   WP_147916367.1 WP_147916367.1 TetR family transcriptional regulator
6(1)   WP_031017381.1 WP_031017381.1 TetR/AcrR family transcriptional regulator
6(1)   WP_156742036.1 WP_156742036.1 TetR/AcrR family transcriptional regulator

7(1)   WP_202451649.1 WP_202451649.1 acyl-CoA dehydrogenase family protein
7(1)   WP_006131543.1 WP_006131543.1 MULTISPECIES: acyl-CoA dehydrogenase family protein
7(1)   WP_184443628.1 WP_184443628.1 acyl-CoA dehydrogenase family protein
7(1)   WP_166527909.1 WP_166527909.1 acyl-CoA dehydrogenase

family protein
7(1)   WP_188947756.1 WP_188947756.1 acyl-CoA dehydrogenase family protein
7(1)   WP_128470345.1 WP_128470345.1 acyl-CoA dehydrogenase family protein
7(1)   WP_059205177.1 WP_059205177.1 MULTISPECIES: acyl-CoA dehydrogenase family protein
7(1)   WP_149378853.1 WP_149378853.1 acyl-CoA dehydrogenase family protein
7(1)   WP_266383258.1 WP_266383258.1 acyl-CoA dehydrogenase family protein
7(1)   WP_137306991.1 WP_137306991.1 MULTISPECIES: acyl-CoA dehydrogenase family protein
7(1)   WP_069398842.1 WP_069398842.1 acyl-CoA dehydrogenase family protein
7(1)   WP_189799151.1 WP_189799151.1 acyl-CoA dehydrogenase family protein
7(1)   WP_093739084.1 WP_093739084.1 acyl-CoA dehydrogenase family protein
7(1)   WP_066860320.1 WP_066860320.1 MULTISPECIES: acyl-CoA dehydrogenase family protein
7(1)   WP_039649410.1 WP_039649410.1 MULTISPECIES: acyl-CoA dehydrogenase family protein
7(1)   WP_065148211.1 WP_065148211.1 acyl-CoA dehydrogenase family protein
7(1)   WP_014375114.1 WP_014375114.1 acyl-CoA dehydrogenase family protein
7(1)   WP_280851346.1 WP_280851346.1 acyl-CoA dehydrogenase family protein
7(1)   WP_069742604.1 WP_069742604.1 acyl-CoA dehydrogenase family protein
7(1)   WP_097193142.1 WP_097193142.1 acyl-CoA dehydrogenase family protein
7(2)   WP_176725144.1 WP_176725144.1 MULTISPECIES: acyl-CoA dehydrogenase family protein

8(1)   WP_069398826.1 WP_069398826.1 thioredoxin-dependent thiol peroxidase
8(1)   WP_283320832.1 WP_283320832.1 thioredoxin-dependent thiol peroxidase
8(1)   WP_003880042.1 WP_003880042.1 thioredoxin-dependent

thiol peroxidase
8(1)   WP_070320437.1 WP_070320437.1 MULTISPECIES: thioredoxin-dependent thiol peroxidase
8(1)   WP_263021842.1 WP_263021842.1 thioredoxin-dependent thiol peroxidase
8(1)   WP_259307168.1 WP_259307168.1 thioredoxin-dependent thiol peroxidase
8(1)   WP_278175654.1 WP_278175654.1 thioredoxin-dependent thiol peroxidase
8(1)   WP_006554761.1 WP_006554761.1 MULTISPECIES: thioredoxin-dependent thiol peroxidase
8(1)   WP_148240390.1 WP_148240390.1 thioredoxin-dependent thiol peroxidase
8(1)   WP_199423947.1 WP_199423947.1 peroxiredoxin
8(1)   WP_048342602.1 WP_048342602.1 MULTISPECIES: thioredoxin-dependent thiol peroxidase
8(1)   WP_147796134.1 WP_147796134.1 thioredoxin-dependent thiol peroxidase
8(1)   WP_029289543.1 WP_029289543.1 thioredoxin-dependent thiol peroxidase
8(1)   WP_189158445.1 WP_189158445.1 peroxiredoxin
8(1)   WP_131167305.1 WP_131167305.1 thioredoxin-dependent thiol peroxidase
8(1)   WP_091064410.1 WP_091064410.1 thioredoxin-dependent thiol peroxidase
8(1)   WP_070721257.1 WP_070721257.1 MULTISPECIES: peroxiredoxin
8(1)   WP_212321635.1 WP_212321635.1 peroxiredoxin
8(1)   WP_213011486.1 WP_213011486.1 thioredoxin-dependent thiol peroxidase
8(1)   WP_156742046.1 WP_156742046.1 peroxiredoxin
8(1)   WP_184443612.1 WP_184443612.1 thioredoxin-dependent thiol peroxidase
8(1)   WP_203670374.1 WP_203670374.1 thioredoxin-dependent thiol peroxidase

9(1)   WP_246910422.1 WP_246910422.1 single-stranded DNA-binding protein
9(1)   WP_053071779.1 WP_053071779.1 MULTISPECIES: single-stranded DNA-binding protein
9(1)   WP_259307150.1 WP_259307150.1 single-stranded

DNA-binding protein
9(1) WP_185277588.1 WP_185277588.1 single-stranded DNA-binding protein
9(1) WP_141787615.1 WP_141787615.1 single-stranded DNA-binding protein
9(1) WP_082740322.1 WP_082740322.1 single-stranded DNA-binding protein
9(1) WP_134353682.1 WP_134353682.1 single-stranded DNA-binding protein
9(1) WP_147916393.1 WP_147916393.1 single-stranded DNA-binding protein
9(1) WP_070721220.1 WP_070721220.1 MULTISPECIES: single-stranded DNA-binding protein
9(1) WP_205654058.1 WP_205654058.1 single-stranded DNA-binding protein
9(1) WP_157581647.1 WP_157581647.1 single-stranded DNA-binding protein
9(1) WP_174179903.1 WP_174179903.1 MULTISPECIES: single-stranded DNA-binding protein
9(1) WP_203670389.1 WP_203670389.1 single-stranded DNA-binding protein
9(1) WP_259809481.1 WP_259809481.1 single-stranded DNA-binding protein
9(1) WP_084487475.1 WP_084487475.1 single-stranded DNA-binding protein
9(1) WP_154347592.1 WP_154347592.1 single-stranded DNA-binding protein
9(1) WP_252619912.1 WP_252619912.1 single-stranded DNA-binding protein
9(1) WP_052711063.1 WP_052711063.1 single-stranded DNA-binding protein
9(1) WP_283320809.1 WP_283320809.1 single-stranded DNA-binding protein
9(1) WP_199423934.1 WP_199423934.1 single-stranded DNA-binding protein

10(1) WP_084229135.1 WP_084229135.1 ABC transporter permease
10(1) WP_203670379.1 WP_203670379.1 ABC transporter permease subunit
10(1) WP_203670380.1 WP_203670380.1 ABC transporter

permease subunit
10(1)   WP_246855406.1 WP_246855406.1 MULTISPECIES: ABC transporter permease subunit
10(1)   WP_070320439.1 WP_070320439.1 ABC transporter permease subunit
10(1)   WP_259307161.1 WP_259307161.1 ABC transporter permease
10(1)   WP_085388184.1 WP_085388184.1 MULTISPECIES: ABC transporter permease subunit
10(1)   WP_131167296.1 WP_131167296.1 ABC transporter permease subunit
10(1)   WP_147796142.1 WP_147796142.1 ABC transporter permease
10(1)   WP_106266555.1 WP_106266555.1 MULTISPECIES: ABC transporter permease subunit
10(1)   WP_046528006.1 WP_046528006.1 MULTISPECIES: ABC transporter permease
10(1)   WP_283320825.1 WP_283320825.1 ABC transporter permease subunit
10(1)   WP_029289533.1 WP_029289533.1 ABC transporter permease
10(1)   WP_029289535.1 WP_029289535.1 ABC transporter permease subunit
10(1)   WP_259307162.1 WP_259307162.1 ABC transporter permease subunit
10(1)   WP_131167297.1 WP_131167297.1 ABC transporter permease
10(1)   WP_283320826.1 WP_283320826.1 ABC transporter permease
10(1)   WP_106266557.1 WP_106266557.1 MULTISPECIES: ABC transporter permease subunit
10(1)   WP_144680242.1 WP_144680242.1 ABC transporter permease subunit

11(1)   WP_329243493.1 WP_329243493.1 cellulose binding domain-containing protein
11(1)   WP_091063642.1 WP_091063642.1 cellulose binding domain-containing protein
11(1)   WP_266514975.1 WP_266514975.1 cellulose binding domain-containing protein
11(1)   WP_239068932.1 WP_239068932.1 glycosyl hydrolase

11(1) WP_213011475.1 WP_213011475.1 cellulose binding domain-containing protein
11(1) WP_131167293.1 WP_131167293.1 polysaccharide deacetylase family protein
11(1) WP_263021862.1 WP_263021862.1 cellulose binding domain-containing protein
11(2) WP_093739041.1 WP_093739041.1 MULTISPECIES: cellulose binding domain-containing protein
11(1) WP_031017390.1 WP_031017390.1 chitinase
11(1) WP_330345284.1 WP_330345284.1 cellulose binding domain-containing protein
11(1) WP_101787151.1 WP_101787151.1 polysaccharide deacetylase family protein
11(1) WP_210648814.1 WP_210648814.1 PASTA domain-containing protein
11(1) WP_246910412.1 WP_246910412.1 cellulose binding domain-containing protein
11(1) WP_280907740.1 WP_280907740.1 cellulose binding domain-containing protein
11(1) WP_245716412.1 WP_245716412.1 polysaccharide deacetylase family protein
11(1) WP_141368716.1 WP_141368716.1 cellulose-binding domain-containing protein
11(2) WP_266759087.1 WP_266759087.1 MULTISPECIES: cellulose binding domain-containing protein

12(1) WP_259809491.1 WP_259809491.1 LacI family DNA-binding transcriptional regulator
12(1) WP_137307008.1 WP_137307008.1 MULTISPECIES: LacI family DNA-binding transcriptional regulator
12(1) WP_266514965.1 WP_266514965.1 MULTISPECIES: LacI family DNA-binding transcriptional regulator
12(2) WP_093739051.1 WP_093739051.1 MULTISPECIES: LacI family DNA-binding transcriptional regulator
12(1) WP_052336380.1 WP_052336380.1 LacI family DNA-binding transcriptional regulator
12(1) WP_057609685.1 WP_057609685.1 MULTISPECIES: LacI family DNA-binding transcriptional regulator
12(2) WP_266759070.1 WP_266759070.1 MULTISPECIES: LacI family DNA-binding transcriptional regulator
12(1) WP_031017394.1 WP_031017394.1 MULTISPECIES: LacI

family DNA-binding transcriptional regulator
12(1)   WP_312007929.1 WP_312007929.1 LacI family DNA-binding transcriptional regulator
12(1)   WP_189799165.1 WP_189799165.1 LacI family DNA-binding transcriptional regulator
12(1)   WP_266383304.1 WP_266383304.1 LacI family DNA-binding transcriptional regulator
12(1)   WP_205654063.1 WP_205654063.1 LacI family DNA-binding transcriptional regulator
12(1)   WP_039649277.1 WP_039649277.1 MULTISPECIES: LacI family DNA-binding transcriptional regulator
12(1)   WP_310112505.1 WP_310112505.1 LacI family DNA-binding transcriptional regulator
12(1)   WP_280851332.1 WP_280851332.1 LacI family DNA-binding transcriptional regulator
12(1)   WP_200712225.1 WP_200712225.1 MULTISPECIES: LacI family DNA-binding transcriptional regulator

13(1)   WP_069739542.1 WP_069739542.1 MULTISPECIES: helix-turn-helix domain-containing protein
13(1)   WP_312007931.1 WP_312007931.1 helix-turn-helix domain-containing protein
13(1)   WP_062049626.1 WP_062049626.1 helix-turn-helix domain-containing protein
13(1)   WP_147916375.1 WP_147916375.1 AraC family transcriptional regulator
13(1)   WP_266514959.1 WP_266514959.1 MULTISPECIES: helix-turn-helix domain-containing protein
13(1)   WP_201049329.1 WP_201049329.1 helix-turn-helix domain-containing protein
13(1)   WP_006131556.1 WP_006131556.1 MULTISPECIES: helix-turn-helix domain-containing protein
13(2)   WP_093739064.1 WP_093739064.1 MULTISPECIES: helix-turn-helix domain-containing protein
13(1)   WP_189158434.1 WP_189158434.1 helix-turn-helix domain-containing protein
13(1)   WP_234535546.1 WP_234535546.1 helix-turn-helix domain-containing protein
13(1)   WP_189799163.1 WP_189799163.1 helix-turn-helix domain-containing protein
13(1)   WP_147916390.1 WP_147916390.1 helix-turn-helix

domain-containing protein
13(1)   WP_057609680.1 WP_057609680.1 MULTISPECIES: helix-turn-helix domain-containing protein
13(1)   WP_280851336.1 WP_280851336.1 helix-turn-helix domain-containing protein
13(1)   WP_039649272.1 WP_039649272.1 MULTISPECIES: helix-turn-helix domain-containing protein
13(2)   WP_266759062.1 WP_266759062.1 MULTISPECIES: helix-turn-helix domain-containing protein

14(1)   WP_029110073.1 WP_029110073.1 Ig-like domain-containing protein
14(1)   WP_109148412.1 WP_109148412.1 MULTISPECIES: Ig-like domain-containing protein
14(1)   WP_232326895.1 WP_232326895.1 Ig-like domain-containing protein
14(1)   WP_283663408.1 WP_283663408.1 Ig-like domain-containing protein
14(1)   WP_066860342.1 WP_066860342.1 MULTISPECIES: Ig-like domain-containing protein
14(1)   WP_225223300.1 WP_225223300.1 MULTISPECIES: Ig-like domain-containing protein
14(1)   WP_187764536.1 WP_187764536.1 Ig-like domain-containing protein
14(1)   WP_003880039.1 WP_003880039.1 MULTISPECIES: Ig-like domain-containing protein
14(1)   WP_315912376.1 WP_315912376.1 Ig-like domain-containing protein
14(1)   WP_278175638.1 WP_278175638.1 Ig-like domain-containing protein
14(1)   WP_278175639.1 WP_278175639.1 Ig-like domain-containing protein
14(1)   WP_156736625.1 WP_156736625.1 L,D-transpeptidase, partial
14(1)   WP_229693945.1 WP_229693945.1 Ig-like domain-containing protein
14(1)   WP_024100531.1 WP_024100531.1 MULTISPECIES: Ig-like domain-containing protein
14(1)   WP_184443615.1 WP_184443615.1 Ig-like domain-containing protein
14(1)   WP_091063616.1 WP_091063616.1 Ig-like

domain-containing protein
14(1) WP_091063620.1 WP_091063620.1 Ig-like
domain-containing protein
14(1) WP_063060739.1 WP_063060739.1 Ig-like
domain-containing protein

15(1) WP_147916370.1 WP_147916370.1 CoA-acylating
methylmalonate-semialdehyde dehydrogenase
15(1) WP_051005136.1 WP_051005136.1 acetoacetate--CoA
ligase
15(1) WP_251746092.1 WP_251746092.1 aldehyde dehydrogenase
family protein
15(1) WP_144680228.1 WP_144680228.1 class I
adenylate-forming enzyme family protein
15(1) WP_283663420.1 WP_283663420.1 aldehyde dehydrogenase
family protein
15(1) WP_189158431.1 WP_189158431.1 AMP-binding protein
15(1) WP_134353674.1 WP_134353674.1
long-chain-fatty-acid--CoA ligase
15(1) WP_261559523.1 WP_261559523.1 aldehyde dehydrogenase
family protein
15(1) WP_232303938.1 WP_232303938.1 aldehyde dehydrogenase
family protein
15(1) WP_283663413.1 WP_283663413.1 AMP-binding protein
15(1) WP_283320816.1 WP_283320816.1 aldehyde dehydrogenase
family protein
15(1) WP_250357391.1 WP_250357391.1 AMP-binding protein
15(1) WP_150242312.1 WP_150242312.1 amino acid adenylation
domain-containing protein, partial
15(1) WP_010696042.1 WP_010696042.1 AMP-binding protein
15(1) WP_246910449.1 WP_246910449.1 AMP-binding protein
15(1) WP_256095699.1 WP_256095699.1 fatty acid--CoA ligase
family protein
15(1) WP_010696031.1 WP_010696031.1 non-ribosomal peptide
synthetase

16(1) WP_039649271.1 WP_039649271.1 MULTISPECIES: universal
stress protein
16(1) WP_312007932.1 WP_312007932.1 universal stress
protein

16(2)   WP_266759060.1 WP_266759060.1 MULTISPECIES: universal stress protein
16(1)   WP_069739543.1 WP_069739543.1 MULTISPECIES: universal stress protein
16(3)   WP_020123262.1 WP_020123262.1 MULTISPECIES: universal stress protein
16(1)   WP_067236238.1 WP_067236238.1 MULTISPECIES: universal stress protein
16(2)   WP_093739066.1 WP_093739066.1 MULTISPECIES: universal stress protein
16(1)   WP_266514955.1 WP_266514955.1 MULTISPECIES: universal stress protein
16(1)   WP_019065779.1 WP_019065779.1 MULTISPECIES: universal stress protein
16(1)   WP_125498912.1 WP_125498912.1 MULTISPECIES: universal stress protein
16(1)   WP_089105170.1 WP_089105170.1 MULTISPECIES: universal stress protein
16(1)   WP_006131555.1 WP_006131555.1 MULTISPECIES: universal stress protein

17(1)   WP_137307001.1 WP_137307001.1 MULTISPECIES: glutamine--fructose-6-phosphate transaminase (isomerizing)
17(1)   WP_312007933.1 WP_312007933.1 glutamine--fructose-6-phosphate transaminase (isomerizing)
17(1)   WP_069742612.1 WP_069742612.1 glutamine--fructose-6-phosphate transaminase (isomerizing)
17(2)   WP_093739068.1 WP_093739068.1 MULTISPECIES: glutamine--fructose-6-phosphate transaminase (isomerizing)
17(1)   WP_200712217.1 WP_200712217.1 MULTISPECIES: glutamine--fructose-6-phosphate transaminase (isomerizing)
17(1)   WP_189799162.1 WP_189799162.1 glutamine--fructose-6-phosphate transaminase (isomerizing)
17(2)   WP_266759056.1 WP_266759056.1 MULTISPECIES: glutamine--fructose-6-phosphate transaminase (isomerizing)
17(1)   WP_234535552.1 WP_234535552.1 glutamine--fructose-6-phosphate transaminase (isomerizing)
17(1)   WP_266514952.1 WP_266514952.1 MULTISPECIES: glutamine--fructose-6-phosphate transaminase (isomerizing)
17(2)   WP_057609678.1 WP_057609678.1 MULTISPECIES: glutamine--fructose-6-phosphate transaminase (isomerizing)

17(1)   WP_039649267.1 WP_039649267.1 MULTISPECIES: glutamine--fructose-6-phosphate transaminase (isomerizing)
17(1)   WP_280851338.1 WP_280851338.1 glutamine--fructose-6-phosphate transaminase (isomerizing)
17(1)   WP_031017396.1 WP_031017396.1 glutamine--fructose-6-phosphate transaminase (isomerizing)

18(2)   WP_093739074.1 WP_093739074.1 MULTISPECIES: GNAT family N-acetyltransferase
18(1)   WP_234535561.1 WP_234535561.1 GNAT family N-acetyltransferase
18(1)   WP_059205172.1 WP_059205172.1 MULTISPECIES: GNAT family N-acetyltransferase
18(1)   WP_006131548.1 WP_006131548.1 MULTISPECIES: GNAT family N-acetyltransferase
18(2)   WP_266759044.1 WP_266759044.1 MULTISPECIES: GNAT family N-acetyltransferase
18(1)   WP_069742610.1 WP_069742610.1 MULTISPECIES: GNAT family N-acetyltransferase
18(1)   WP_189799157.1 WP_189799157.1 GNAT family N-acetyltransferase
18(1)   WP_266514940.1 WP_266514940.1 GNAT family N-acetyltransferase
18(1)   WP_329243451.1 WP_329243451.1 GNAT family N-acetyltransferase
18(1)   WP_200712213.1 WP_200712213.1 MULTISPECIES: GNAT family N-acetyltransferase
18(1)   WP_039649260.1 WP_039649260.1 MULTISPECIES: GNAT family N-acetyltransferase
18(1)   WP_237710551.1 WP_237710551.1 GNAT family N-acetyltransferase
18(1)   WP_137306996.1 WP_137306996.1 MULTISPECIES: GNAT family N-acetyltransferase
18(1)   WP_280898533.1 WP_280898533.1 GNAT family N-acetyltransferase

19(1)   WP_093739070.1 WP_093739070.1 hypothetical protein
19(1)   WP_202451648.1 WP_202451648.1 hypothetical protein
19(1)   WP_006131553.1 WP_006131553.1 MULTISPECIES: hypothetical protein

19(1)   WP_003968990.1 WP_003968990.1 MULTISPECIES: hypothetical protein
19(1)   WP_189799161.1 WP_189799161.1 hypothetical protein
19(2)   WP_057609677.1 WP_057609677.1 MULTISPECIES: hypothetical protein
19(1)   WP_020123260.1 WP_020123260.1 MULTISPECIES: hypothetical protein
19(1)   WP_137307000.1 WP_137307000.1 MULTISPECIES: hypothetical protein
19(1)   WP_019065776.1 WP_019065776.1 MULTISPECIES: hypothetical protein
19(1)   WP_210921833.1 WP_210921833.1 MULTISPECIES: hypothetical protein
19(1)   WP_039649264.1 WP_039649264.1 MULTISPECIES: hypothetical protein
19(1)   WP_089105178.1 WP_089105178.1 hypothetical protein
19(2)   WP_266759054.1 WP_266759054.1 MULTISPECIES: hypothetical protein

20(1)   WP_189799156.1 WP_189799156.1 lysine N(6)-hydroxylase/L-ornithine N(5)-oxygenase family protein
20(1)   WP_059205173.1 WP_059205173.1 MULTISPECIES: SidA/IucD/PvdA family monooxygenase
20(1)   WP_266514938.1 WP_266514938.1 MULTISPECIES: SidA/IucD/PvdA family monooxygenase
20(1)   WP_202451646.1 WP_202451646.1 lysine N(6)-hydroxylase/L-ornithine N(5)-oxygenase family protein
20(2)   WP_266759042.1 WP_266759042.1 MULTISPECIES: lysine N(6)-hydroxylase/L-ornithine N(5)-oxygenase family protein
20(1)   WP_329243448.1 WP_329243448.1 SidA/IucD/PvdA family monooxygenase
20(1)   WP_234535563.1 WP_234535563.1 SidA/IucD/PvdA family monooxygenase
20(1)   WP_280894671.1 WP_280894671.1 lysine N(6)-hydroxylase/L-ornithine N(5)-oxygenase family protein
20(1)   WP_069739548.1 WP_069739548.1 MULTISPECIES: lysine N(6)-hydroxylase/L-ornithine N(5)-oxygenase family protein
20(1)   WP_093739138.1 WP_093739138.1 lysine N(6)-hydroxylase/L-ornithine N(5)-oxygenase family protein
20(1)   WP_201049326.1 WP_201049326.1 SidA/IucD/PvdA family monooxygenase

20(1)   WP_039649257.1 WP_039649257.1 MULTISPECIES: lysine N(6)-hydroxylase/L-ornithine N(5)-oxygenase family protein
20(1)   WP_137306995.1 WP_137306995.1 MULTISPECIES: SidA/IucD/PvdA family monooxygenase
20(1)   WP_006131547.1 WP_006131547.1 MULTISPECIES: lysine N(6)-hydroxylase/L-ornithine N(5)-oxygenase family protein

21(1)   WP_330345290.1 WP_330345290.1 IucA/IucC family siderophore biosynthesis protein
21(1)   WP_031017402.1 WP_031017402.1 IucA/IucC family siderophore biosynthesis protein
21(1)   WP_201049327.1 WP_201049327.1 IucA/IucC family siderophore biosynthesis protein
21(1)   WP_189799158.1 WP_189799158.1 IucA/IucC family siderophore biosynthesis protein
21(1)   WP_059205171.1 WP_059205171.1 MULTISPECIES: IucA/IucC family siderophore biosynthesis protein
21(1)   WP_329243454.1 WP_329243454.1 IucA/IucC family siderophore biosynthesis protein
21(2)   WP_266759046.1 WP_266759046.1 MULTISPECIES: IucA/IucC family siderophore biosynthesis protein
21(1)   WP_039649262.1 WP_039649262.1 MULTISPECIES: IucA/IucC family siderophore biosynthesis protein
21(1)   WP_280907741.1 WP_280907741.1 IucA/IucC family siderophore biosynthesis protein
21(1)   WP_069739546.1 WP_069739546.1 MULTISPECIES: IucA/IucC family siderophore biosynthesis protein
21(1)   WP_234535559.1 WP_234535559.1 IucA/IucC family siderophore biosynthesis protein
21(2)   WP_093739072.1 WP_093739072.1 IucA/IucC family siderophore biosynthesis protein
21(1)   WP_266514942.1 WP_266514942.1 IucA/IucC family siderophore biosynthesis protein

22(1)   WP_059205174.1 WP_059205174.1 MULTISPECIES: lysine decarboxylase DesA
22(1)   WP_189799155.1 WP_189799155.1 aspartate aminotransferase family protein
22(1)   WP_234535565.1 WP_234535565.1 lysine decarboxylase DesA

22(1)   WP_330345291.1 WP_330345291.1 lysine decarboxylase DesA
22(1)   WP_069742609.1 WP_069742609.1 aspartate aminotransferase family protein
22(2)   WP_266759040.1 WP_266759040.1 MULTISPECIES: lysine decarboxylase DesA
22(1)   WP_201049325.1 WP_201049325.1 lysine decarboxylase DesA
22(1)   WP_280894670.1 WP_280894670.1 lysine decarboxylase DesA
22(1)   WP_039649254.1 WP_039649254.1 MULTISPECIES: lysine decarboxylase DesA
22(1)   WP_266514937.1 WP_266514937.1 lysine decarboxylase DesA
22(2)   WP_093739076.1 WP_093739076.1 MULTISPECIES: aspartate aminotransferase family protein
22(1)   WP_329243445.1 WP_329243445.1 lysine decarboxylase DesA
22(1)   WP_031017407.1 WP_031017407.1 lysine decarboxylase DesA

23(1)   WP_175441351.1 WP_175441351.1 sigma-70 family RNA polymerase sigma factor
23(1)   WP_246607920.1 WP_246607920.1 sigma-70 family RNA polymerase sigma factor
23(1)   WP_263021846.1 WP_263021846.1 sigma-70 family RNA polymerase sigma factor
23(1)   WP_187437665.1 WP_187437665.1 sigma-70 family RNA polymerase sigma factor
23(1)   WP_148757154.1 WP_148757154.1 sigma-70 family RNA polymerase sigma factor
23(1)   WP_255279850.1 WP_255279850.1 hypothetical protein
23(1)   WP_278181959.1 WP_278181959.1 sigma-70 family RNA polymerase sigma factor
23(1)   WP_185277583.1 WP_185277583.1 sigma-70 family RNA polymerase sigma factor
23(1)   WP_213011484.1 WP_213011484.1 sigma-70 family RNA polymerase sigma factor
23(1)   WP_263021859.1 WP_263021859.1 right-handed parallel beta-helix repeat-containing protein
23(1)   WP_255279851.1 WP_255279851.1 sigma-70 family RNA

polymerase sigma factor
23(1) WP_132049022.1 WP_132049022.1 sigma-70 family RNA polymerase sigma factor
23(1) WP_132049020.1 WP_132049020.1 sigma-70 family RNA polymerase sigma factor
23(1) WP_091063598.1 WP_091063598.1 sigma-70 family RNA polymerase sigma factor

24(1) WP_241656205.1 WP_241656205.1 HAMP domain-containing sensor histidine kinase
24(2) WP_266759089.1 WP_266759089.1 MULTISPECIES: HAMP domain-containing sensor histidine kinase
24(1) WP_213011470.1 WP_213011470.1 hybrid sensor histidine kinase/response regulator
24(1) WP_213011471.1 WP_213011471.1 ATP-binding protein
24(1) WP_187437666.1 WP_187437666.1 HAMP domain-containing sensor histidine kinase
24(1) WP_028472266.1 WP_028472266.1 PAS domain-containing sensor histidine kinase
24(1) WP_143913346.1 WP_143913346.1 MtrAB system histidine kinase MtrB
24(1) WP_266514977.1 WP_266514977.1 MULTISPECIES: HAMP domain-containing sensor histidine kinase
24(1) WP_315912374.1 WP_315912374.1 HAMP domain-containing sensor histidine kinase
24(1) WP_156391073.1 WP_156391073.1 MULTISPECIES: ATP-binding protein
24(1) WP_266803684.1 WP_266803684.1 HAMP domain-containing sensor histidine kinase
24(1) WP_006131564.1 WP_006131564.1 MULTISPECIES: HAMP domain-containing sensor histidine kinase
24(1) WP_056153702.1 WP_056153702.1 MULTISPECIES: HAMP domain-containing sensor histidine kinase

25(1) WP_149378852.1 WP_149378852.1 biotin carboxylase N-terminal domain-containing protein
25(1) WP_039649247.1 WP_039649247.1 MULTISPECIES: biotin carboxylase N-terminal domain-containing protein
25(1) WP_097193143.1 WP_097193143.1 biotin carboxylase N-terminal domain-containing protein

25(1)   WP_166527908.1 WP_166527908.1 biotin carboxylase N-terminal domain-containing protein
25(1)   WP_014375113.1 WP_014375113.1 biotin carboxylase N-terminal domain-containing protein
25(1)   WP_031017415.1 WP_031017415.1 acetyl/propionyl/methylcrotonyl-CoA carboxylase subunit alpha
25(2)   WP_093739078.1 WP_093739078.1 MULTISPECIES: biotin carboxylase N-terminal domain-containing protein
25(1)   WP_067178796.1 WP_067178796.1 acetyl/propionyl/methylcrotonyl-CoA carboxylase subunit alpha
25(1)   WP_184443627.1 WP_184443627.1 acetyl/propionyl/methylcrotonyl-CoA carboxylase subunit alpha
25(1)   WP_064913724.1 WP_064913724.1 acetyl/propionyl/methylcrotonyl-CoA carboxylase subunit alpha
25(1)   WP_280907742.1 WP_280907742.1 acetyl/propionyl/methylcrotonyl-CoA carboxylase subunit alpha
25(1)   WP_069398841.1 WP_069398841.1 acetyl/propionyl/methylcrotonyl-CoA carboxylase subunit alpha

26(2)   WP_093739043.1 WP_093739043.1 MULTISPECIES: GH1 family beta-glucosidase
26(1)   WP_266383316.1 WP_266383316.1 GH1 family beta-glucosidase
26(1)   WP_189799166.1 WP_189799166.1 GH1 family beta-glucosidase
26(1)   WP_201049335.1 WP_201049335.1 family 1 glycosylhydrolase
26(1)   WP_280894678.1 WP_280894678.1 GH1 family beta-glucosidase
26(1)   WP_137307011.1 WP_137307011.1 MULTISPECIES: GH1 family beta-glucosidase
26(1)   WP_059205163.1 WP_059205163.1 MULTISPECIES: GH1 family beta-glucosidase
26(2)   WP_266759076.1 WP_266759076.1 MULTISPECIES: GH1 family beta-glucosidase
26(1)   WP_006131562.1 WP_006131562.1 MULTISPECIES: GH1 family beta-glucosidase
26(1)   WP_266514972.1 WP_266514972.1 MULTISPECIES: GH1 family beta-glucosidase
26(1)   WP_283320830.1 WP_283320830.1 GH1 family beta-glucosidase

27(1) WP_280894669.1 WP_280894669.1 siderophore-interacting protein
27(1) WP_031017409.1 WP_031017409.1 MULTISPECIES: siderophore-interacting protein
27(1) WP_266514935.1 WP_266514935.1 siderophore-interacting protein
27(1) WP_234535567.1 WP_234535567.1 siderophore-interacting protein
27(1) WP_201049324.1 WP_201049324.1 siderophore-interacting protein
27(1) WP_010696027.1 WP_010696027.1 siderophore-interacting protein
27(1) WP_189799154.1 WP_189799154.1 siderophore-interacting protein
27(2) WP_266759038.1 WP_266759038.1 MULTISPECIES: siderophore-interacting protein
27(1) WP_059205175.1 WP_059205175.1 MULTISPECIES: siderophore-interacting protein
27(1) WP_326780385.1 WP_326780385.1 siderophore-interacting protein
27(1) WP_329243443.1 WP_329243443.1 siderophore-interacting protein
27(1) WP_240103243.1 WP_240103243.1 siderophore-interacting protein

28(1) WP_201049323.1 WP_201049323.1 ABC transporter substrate-binding protein
28(1) WP_234535569.1 WP_234535569.1 ABC transporter substrate-binding protein
28(1) WP_280851345.1 WP_280851345.1 ABC transporter substrate-binding protein
28(2) WP_266759036.1 WP_266759036.1 MULTISPECIES: ABC transporter substrate-binding protein
28(1) WP_329243440.1 WP_329243440.1 ABC transporter substrate-binding protein
28(1) WP_330345292.1 WP_330345292.1 ABC transporter substrate-binding protein
28(1) WP_240103244.1 WP_240103244.1 ABC transporter substrate-binding protein

28(1)   WP_189799153.1 WP_189799153.1 ABC transporter substrate-binding protein
28(1)   WP_266514933.1 WP_266514933.1 ABC transporter substrate-binding protein
28(1)   WP_006131544.1 WP_006131544.1 MULTISPECIES: ABC transporter substrate-binding protein
28(1)   WP_156742033.1 WP_156742033.1 ABC transporter substrate-binding protein
28(1)   WP_059205176.1 WP_059205176.1 MULTISPECIES: ABC transporter substrate-binding protein

29(1)   WP_129235643.1 WP_129235643.1 MULTISPECIES: thioesterase family protein
29(1)   WP_046528017.1 WP_046528017.1 MULTISPECIES: thioesterase family protein
29(1)   WP_259307152.1 WP_259307152.1 thioesterase family protein
29(1)   WP_199423930.1 WP_199423930.1 thioesterase family protein
29(1)   WP_246910414.1 WP_246910414.1 thioesterase family protein
29(1)   WP_252619905.1 WP_252619905.1 thioesterase family protein
29(1)   WP_141368732.1 WP_141368732.1 MULTISPECIES: thioesterase family protein
29(1)   WP_131490980.1 WP_131490980.1 MULTISPECIES: thioesterase family protein
29(1)   WP_045877966.1 WP_045877966.1 hotdog domain-containing protein
29(1)   WP_203670394.1 WP_203670394.1 thioesterase family protein
29(1)   WP_148240393.1 WP_148240393.1 thioesterase family protein
29(1)   WP_185277585.1 WP_185277585.1 thioesterase family protein

30(1)   WP_156742043.1 WP_156742043.1 prolyl aminopeptidase
30(1)   WP_010596348.1 WP_010596348.1 MULTISPECIES: alpha/beta hydrolase
30(1)   WP_043642042.1 WP_043642042.1 alpha/beta hydrolase

30(1) WP_168211686.1 WP_168211686.1 alpha/beta hydrolase
30(1) WP_191902812.1 WP_191902812.1 alpha/beta hydrolase
30(1) WP_283320820.1 WP_283320820.1 dienelactone hydrolase family protein
30(1) WP_307844833.1 WP_307844833.1 alpha/beta hydrolase
30(1) WP_110047923.1 WP_110047923.1 alpha/beta hydrolase
30(1) WP_187368371.1 WP_187368371.1 alpha/beta hydrolase
30(1) WP_147796146.1 WP_147796146.1 dienelactone hydrolase family protein
30(1) WP_210648817.1 WP_210648817.1 alpha/beta hydrolase
30(1) WP_024100537.1 WP_024100537.1 MULTISPECIES: alpha/beta hydrolase

31(1) WP_283320828.1 WP_283320828.1 NAD(P)/FAD-dependent oxidoreductase
31(1) WP_246910445.1 WP_246910445.1 NAD(P)/FAD-dependent oxidoreductase
31(1) WP_144680260.1 WP_144680260.1 NAD(P)/FAD-dependent oxidoreductase
31(1) WP_203670377.1 WP_203670377.1 NAD(P)/FAD-dependent oxidoreductase
31(1) WP_320965252.1 WP_320965252.1 NAD(P)/FAD-dependent oxidoreductase
31(1) WP_029289539.1 WP_029289539.1 NAD(P)/FAD-dependent oxidoreductase
31(1) WP_189158429.1 WP_189158429.1 FAD-dependent oxidoreductase
31(1) WP_258133863.1 WP_258133863.1 MULTISPECIES: NAD(P)/FAD-dependent oxidoreductase
31(1) WP_233421197.1 WP_233421197.1 NAD(P)/FAD-dependent oxidoreductase
31(1) WP_259809492.1 WP_259809492.1 dihydrolipoyl dehydrogenase
31(1) WP_259307164.1 WP_259307164.1 NAD(P)/FAD-dependent oxidoreductase
31(1) WP_147796140.1 WP_147796140.1 NAD(P)/FAD-dependent oxidoreductase

32(1) WP_110047899.1 WP_110047899.1 beta-N-acetylhexosaminidase

32(1)   WP_323178399.1 WP_323178399.1
beta-N-acetylhexosaminidase
32(2)   WP_093739136.1 WP_093739136.1 MULTISPECIES:
beta-N-acetylhexosaminidase
32(1)   WP_150242272.1 WP_150242272.1
beta-N-acetylhexosaminidase
32(1)   WP_308401904.1 WP_308401904.1
beta-N-acetylhexosaminidase
32(1)   WP_201049375.1 WP_201049375.1
beta-N-acetylhexosaminidase
32(2)   WP_266759048.1 WP_266759048.1 MULTISPECIES:
beta-N-acetylhexosaminidase
32(1)   WP_308115084.1 WP_308115084.1
beta-N-acetylhexosaminidase
32(1)   WP_031017400.1 WP_031017400.1
beta-N-acetylhexosaminidase
32(1)   WP_042281214.1 WP_042281214.1 MULTISPECIES:
beta-N-acetylhexosaminidase

33(2)   WP_093739049.1 WP_093739049.1 MULTISPECIES:
extracellular solute-binding protein
33(2)   WP_266759072.1 WP_266759072.1 ABC transporter
substrate-binding protein
33(1)   WP_039649279.1 WP_039649279.1 MULTISPECIES: ABC
transporter substrate-binding protein
33(1)   WP_059205164.1 WP_059205164.1 MULTISPECIES: ABC
transporter substrate-binding protein
33(1)   WP_006131559.1 WP_006131559.1 MULTISPECIES:
extracellular solute-binding protein
33(1)   WP_280851331.1 WP_280851331.1 ABC transporter
substrate-binding protein
33(1)   WP_200712226.1 WP_200712226.1 MULTISPECIES: ABC
transporter substrate-binding protein
33(1)   WP_266514967.1 WP_266514967.1 MULTISPECIES: ABC
transporter substrate-binding protein
33(1)   WP_137307009.1 WP_137307009.1 MULTISPECIES:
extracellular solute-binding protein
33(1)   WP_329243477.1 WP_329243477.1 ABC transporter
substrate-binding protein

34(1) WP_147916376.1 WP_147916376.1 MFS transporter
34(1) WP_185277598.1 WP_185277598.1 MFS transporter
34(1) WP_221632429.1 WP_221632429.1 MFS transporter
34(1) WP_029535362.1 WP_029535362.1 MDR family MFS transporter
34(1) WP_141787612.1 WP_141787612.1 MFS transporter
34(1) WP_246910432.1 WP_246910432.1 MFS transporter
34(1) WP_131167290.1 WP_131167290.1 MFS transporter
34(1) WP_069742608.1 WP_069742608.1 MULTISPECIES: MFS transporter
34(1) WP_144680232.1 WP_144680232.1 MFS transporter
34(1) WP_185278771.1 WP_185278771.1 MFS transporter
34(1) WP_110047922.1 WP_110047922.1 MFS transporter

35(1) WP_029289529.1 WP_029289529.1 hypothetical protein
35(1) WP_203670381.1 WP_203670381.1 hypothetical protein
35(1) WP_106266561.1 WP_106266561.1 MULTISPECIES: hypothetical protein
35(1) WP_144680244.1 WP_144680244.1 hypothetical protein
35(1) WP_156742042.1 WP_156742042.1 hypothetical protein
35(1) WP_283321848.1 WP_283321848.1 hypothetical protein
35(1) WP_048342603.1 WP_048342603.1 MULTISPECIES: hypothetical protein
35(1) WP_199423944.1 WP_199423944.1 hypothetical protein
35(1) WP_070320440.1 WP_070320440.1 hypothetical protein
35(1) WP_147797184.1 WP_147797184.1 hypothetical protein
35(1) WP_259307159.1 WP_259307159.1 hypothetical protein

36(1) WP_067142838.1 WP_067142838.1 MULTISPECIES: carboxyl transferase domain-containing protein
36(1) WP_166527906.1 WP_166527906.1 carboxyl transferase domain-containing protein
36(1) WP_061265148.1 WP_061265148.1 MULTISPECIES: carboxyl transferase domain-containing protein
36(1) WP_014375112.1 WP_014375112.1 carboxyl transferase domain-containing protein
36(1) WP_184443626.1 WP_184443626.1 carboxyl transferase domain-containing protein
36(1) WP_097193144.1 WP_097193144.1 carboxyl transferase domain-containing protein

36(1)   WP_069398840.1 WP_069398840.1 methylcrotonoyl-CoA carboxylase
36(2)   WP_176725146.1 WP_176725146.1 MULTISPECIES: carboxyl transferase domain-containing protein
36(1)   WP_149378851.1 WP_149378851.1 carboxyl transferase domain-containing protein

37(1)   WP_239068967.1 WP_239068967.1 co-chaperone GroES
37(1)   WP_210648829.1 WP_210648829.1 co-chaperone GroES
37(1)   WP_255596302.1 WP_255596302.1 co-chaperone GroES
37(1)   WP_246855405.1 WP_246855405.1 MULTISPECIES: co-chaperone GroES
37(1)   WP_070320444.1 WP_070320444.1 MULTISPECIES: co-chaperone GroES
37(1)   WP_199423989.1 WP_199423989.1 co-chaperone GroES
37(1)   WP_259310269.1 WP_259310269.1 co-chaperone GroES
37(1)   WP_138875249.1 WP_138875249.1 co-chaperone GroES
37(1)   WP_048342614.1 WP_048342614.1 MULTISPECIES: co-chaperone GroES
37(1)   WP_051681791.1 WP_051681791.1 co-chaperone GroES

38(1)   WP_070704854.1 WP_070704854.1 PrsW family intramembrane metalloprotease
38(1)   WP_212321615.1 WP_212321615.1 PrsW family intramembrane metalloprotease
38(1)   WP_056917808.1 WP_056917808.1 MULTISPECIES: PrsW family intramembrane metalloprotease
38(1)   WP_185973091.1 WP_185973091.1 PrsW family intramembrane metalloprotease
38(1)   WP_056154178.1 WP_056154178.1 MULTISPECIES: PrsW family intramembrane metalloprotease
38(1)   WP_259809484.1 WP_259809484.1 PrsW family intramembrane metalloprotease
38(1)   WP_138875256.1 WP_138875256.1 PrsW family intramembrane metalloprotease
38(1)   WP_252619916.1 WP_252619916.1 PrsW family intramembrane metalloprotease
38(1)   WP_141787606.1 WP_141787606.1 PrsW family intramembrane metalloprotease
38(1)   WP_210648811.1 WP_210648811.1 PrsW family

intramembrane metalloprotease

39(1)   WP_067178803.1 WP_067178803.1 DUF853 family protein
39(1)   WP_283663410.1 WP_283663410.1 DUF853 family protein
39(1)   WP_184443619.1 WP_184443619.1 DUF853 family protein
39(1)   WP_029110087.1 WP_029110087.1 DUF853 family protein
39(1)   WP_003880031.1 WP_003880031.1 helicase HerA-like domain-containing protein
39(1)   WP_067781071.1 WP_067781071.1 MULTISPECIES: DUF853 family protein, partial
39(1)   WP_254584113.1 WP_254584113.1 DUF853 family protein
39(1)   WP_189158436.1 WP_189158436.1 DUF853 family protein
39(1)   WP_069398833.1 WP_069398833.1 DUF853 family protein
39(1)   WP_149378845.1 WP_149378845.1 DUF853 family protein

40(1)   WP_150242292.1 WP_150242292.1 substrate-binding domain-containing protein
40(1)   WP_255279848.1 WP_255279848.1 VWA domain-containing protein
40(1)   WP_017534255.1 WP_017534255.1 MULTISPECIES: substrate-binding domain-containing protein
40(1)   WP_110048082.1 WP_110048082.1 substrate-binding domain-containing protein
40(1)   WP_148757150.1 WP_148757150.1 substrate-binding domain-containing protein
40(1)   WP_132049018.1 WP_132049018.1 substrate-binding domain-containing protein
40(1)   WP_326338223.1 WP_326338223.1 substrate-binding domain-containing protein
40(1)   WP_110047909.1 WP_110047909.1 substrate-binding and VWA domain-containing protein
40(1)   WP_150242290.1 WP_150242290.1 substrate-binding domain-containing protein
40(1)   WP_255279847.1 WP_255279847.1 substrate-binding domain-containing protein

41(1)   WP_280894674.1 WP_280894674.1 metallophosphoesterase family protein
41(1)   WP_189799160.1 WP_189799160.1 metallophosphoesterase

family protein
41(1) WP_059205168.1 WP_059205168.1 MULTISPECIES: metallophosphoesterase family protein
41(1) WP_234535554.1 WP_234535554.1 metallophosphoesterase family protein
41(1) WP_329243457.1 WP_329243457.1 metallophosphoesterase family protein
41(2) WP_266759052.1 WP_266759052.1 MULTISPECIES: metallophosphoesterase family protein
41(1) WP_266514947.1 WP_266514947.1 MULTISPECIES: metallophosphoesterase family protein
41(1) WP_200712216.1 WP_200712216.1 MULTISPECIES: metallophosphoesterase family protein
41(1) WP_330345288.1 WP_330345288.1 metallophosphoesterase family protein

42(1) WP_070721244.1 WP_070721244.1 MULTISPECIES: MFS transporter
42(1) WP_063060738.1 WP_063060738.1 MFS transporter
42(1) WP_010596342.1 WP_010596342.1 MULTISPECIES: MFS transporter
42(1) WP_065148210.1 WP_065148210.1 MFS transporter
42(1) WP_067178798.1 WP_067178798.1 MFS transporter
42(1) WP_184443621.1 WP_184443621.1 MFS transporter
42(1) WP_193903552.1 WP_193903552.1 MFS transporter
42(1) WP_149378847.1 WP_149378847.1 MFS transporter
42(1) WP_069398835.1 WP_069398835.1 MFS transporter

43(1) WP_007626708.1 WP_007626708.1 MULTISPECIES: DUF3618 domain-containing protein
43(1) WP_156748726.1 WP_156748726.1 MULTISPECIES: DUF3618 domain-containing protein, partial
43(1) WP_137292537.1 WP_137292537.1 MULTISPECIES: DUF3618 domain-containing protein
43(1) WP_131167304.1 WP_131167304.1 DUF3618 domain-containing protein
43(1) WP_210648830.1 WP_210648830.1 DUF3618 domain-containing protein
43(1) WP_006554762.1 WP_006554762.1 MULTISPECIES: DUF3618 domain-containing protein

43(1)   WP_184443613.1 WP_184443613.1 DUF3618 domain-containing protein
43(1)   WP_003880041.1 WP_003880041.1 MULTISPECIES: DUF3618 domain-containing protein
43(1)   WP_044507168.1 WP_044507168.1 MULTISPECIES: DUF3618 domain-containing protein

44(1)   WP_263021844.1 WP_263021844.1 DsbA family protein
44(1)   WP_278175645.1 WP_278175645.1 thioredoxin domain-containing protein
44(1)   WP_138875253.1 WP_138875253.1 thioredoxin domain-containing protein
44(1)   WP_143913340.1 WP_143913340.1 thioredoxin domain-containing protein
44(1)   WP_213011485.1 WP_213011485.1 thioredoxin domain-containing protein
44(1)   WP_052336383.1 WP_052336383.1 thioredoxin domain-containing protein
44(1)   WP_210648820.1 WP_210648820.1 thioredoxin domain-containing protein
44(1)   WP_056153718.1 WP_056153718.1 MULTISPECIES: thioredoxin domain-containing protein
44(1)   WP_091063591.1 WP_091063591.1 thioredoxin domain-containing protein

45(1)   WP_131167280.1 WP_131167280.1 heavy metal translocating P-type ATPase
45(1)   WP_131167281.1 WP_131167281.1 heavy metal translocating P-type ATPase
45(1)   WP_308738565.1 WP_308738565.1 heavy metal translocating P-type ATPase
45(1)   WP_259809488.1 WP_259809488.1 cation-translocating P-type ATPase
45(1)   WP_245852231.1 WP_245852231.1 heavy metal translocating P-type ATPase
45(1)   WP_259809479.1 WP_259809479.1 cation-translocating P-type ATPase
45(1)   WP_229747475.1 WP_229747475.1 cation-translocating P-type ATPase
45(1)   WP_097193149.1 WP_097193149.1 heavy metal

translocating P-type ATPase
45(1)   WP_190394356.1 WP_190394356.1 hypothetical protein

46(1)   WP_280851337.1 WP_280851337.1 hypothetical protein
46(1)   WP_174673848.1 WP_174673848.1 MULTISPECIES: hypothetical protein
46(1)   WP_266514957.1 WP_266514957.1 MULTISPECIES: hypothetical protein
46(2)   WP_266765699.1 WP_266765699.1 MULTISPECIES: hypothetical protein
46(1)   WP_200712219.1 WP_200712219.1 MULTISPECIES: hypothetical protein
46(1)   WP_234535548.1 WP_234535548.1 hypothetical protein
46(1)   WP_329243462.1 WP_329243462.1 hypothetical protein
46(1)   WP_057609679.1 WP_057609679.1 MULTISPECIES: hypothetical protein

47(1)   WP_266514946.1 WP_266514946.1 DUF4429 domain-containing protein
47(1)   WP_330345289.1 WP_330345289.1 DUF4429 domain-containing protein
47(2)   WP_059205169.1 WP_059205169.1 MULTISPECIES: DUF4429 domain-containing protein
47(1)   WP_069742611.1 WP_069742611.1 DUF4429 domain-containing protein
47(2)   WP_266759050.1 WP_266759050.1 MULTISPECIES: DUF4429 domain-containing protein
47(1)   WP_031017398.1 WP_031017398.1 MULTISPECIES: DUF4429 domain-containing protein
47(1)   WP_201049328.1 WP_201049328.1 DUF4429 domain-containing protein

48(1)   WP_205654065.1 WP_205654065.1 polyprenol phosphomannose-dependent alpha 1,6 mannosyltransferase MptB
48(1)   WP_128470342.1 WP_128470342.1 polyprenol phosphomannose-dependent alpha 1,6 mannosyltransferase MptB
48(1)   WP_236641074.1 WP_236641074.1 polyprenol phosphomannose-dependent alpha 1,6 mannosyltransferase MptB
48(1)   WP_320965261.1 WP_320965261.1 polyprenol

phosphomannose-dependent alpha 1,6 mannosyltransferase MptB
48(1) WP_320965260.1 WP_320965260.1 polyprenol phosphomannose-dependent alpha 1,6 mannosyltransferase MptB
48(1) WP_255248528.1 WP_255248528.1 MULTISPECIES: polyprenol phosphomannose-dependent alpha 1,6 mannosyltransferase MptB
48(1) WP_105552065.1 WP_105552065.1 MULTISPECIES: polyprenol phosphomannose-dependent alpha 1,6 mannosyltransferase MptB
48(1) WP_310112501.1 WP_310112501.1 polyprenol phosphomannose-dependent alpha 1,6 mannosyltransferase MptB

49(1) WP_041561885.1 WP_041561885.1 MULTISPECIES: hypothetical protein
49(1) WP_246178186.1 WP_246178186.1 hypothetical protein
49(1) WP_255279852.1 WP_255279852.1 hypothetical protein
49(1) WP_150242294.1 WP_150242294.1 hypothetical protein
49(1) WP_110047910.1 WP_110047910.1 hypothetical protein
49(1) WP_132049026.1 WP_132049026.1 hypothetical protein
49(1) WP_255279853.1 WP_255279853.1 hypothetical protein

50(1) WP_306254374.1 WP_306254374.1 acyl-CoA thioesterase II
50(1) WP_070319955.1 WP_070319955.1 MULTISPECIES: acyl-CoA thioesterase II
50(1) WP_259310267.1 WP_259310267.1 acyl-CoA thioesterase II
50(1) WP_154347589.1 WP_154347589.1 acyl-CoA thioesterase II
50(1) WP_232304186.1 WP_232304186.1 thioesterase family protein
50(1) WP_205654055.1 WP_205654055.1 acyl-CoA thioesterase II
50(1) WP_134353683.1 WP_134353683.1 acyl-CoA thioesterase II

51(1) WP_280894667.1 WP_280894667.1 hydroxymethylglutaryl-CoA lyase
51(2) WP_093739142.1 WP_093739142.1 MULTISPECIES:

hydroxymethylglutaryl-CoA lyase
51(1) WP_329243437.1 WP_329243437.1
hydroxymethylglutaryl-CoA lyase
51(1) WP_039649250.1 WP_039649250.1 MULTISPECIES:
hydroxymethylglutaryl-CoA lyase
51(1) WP_031017412.1 WP_031017412.1 MULTISPECIES:
hydroxymethylglutaryl-CoA lyase
51(1) WP_189799150.1 WP_189799150.1
hydroxymethylglutaryl-CoA lyase

52(1) WP_280851334.1 WP_280851334.1 LysR family
transcriptional regulator
52(1) WP_185277592.1 WP_185277592.1 LysR substrate-binding
domain-containing protein
52(1) WP_029289522.1 WP_029289522.1 LysR family
transcriptional regulator
52(1) WP_329243467.1 WP_329243467.1 LysR family
transcriptional regulator
52(1) WP_259813891.1 WP_259813891.1 LysR family
transcriptional regulator
52(1) WP_308738561.1 WP_308738561.1 LysR family
transcriptional regulator
52(1) WP_079072204.1 WP_079072204.1 MULTISPECIES: LysR
family transcriptional regulator

53(1) WP_234535550.1 WP_234535550.1 GPR1/FUN34/YaaH family
transporter
53(1) WP_330345287.1 WP_330345287.1 GPR1/FUN34/YaaH family
transporter
53(2) WP_266759058.1 WP_266759058.1 MULTISPECIES:
GPR1/FUN34/YaaH family transporter
53(1) WP_039649269.1 WP_039649269.1 MULTISPECIES:
GPR1/FUN34/YaaH family transporter
53(1) WP_266514953.1 WP_266514953.1 MULTISPECIES:
GPR1/FUN34/YaaH family transporter
53(1) WP_200712218.1 WP_200712218.1 MULTISPECIES:
GPR1/FUN34/YaaH family transporter

54(1) WP_128470478.1 WP_128470478.1 peptide-methionine

(S)-S-oxide reductase MsrA
54(1)   WP_154347593.1 WP_154347593.1 peptide-methionine (S)-S-oxide reductase MsrA
54(1)   WP_185277589.1 WP_185277589.1 peptide-methionine (S)-S-oxide reductase MsrA
54(1)   WP_205654074.1 WP_205654074.1 peptide-methionine (S)-S-oxide reductase MsrA
54(1)   WP_066276756.1 WP_066276756.1 MULTISPECIES: peptide-methionine (S)-S-oxide reductase MsrA
54(1)   WP_096288819.1 WP_096288819.1 MULTISPECIES: peptide-methionine (S)-S-oxide reductase MsrA

55(1)   WP_070721238.1 WP_070721238.1 MULTISPECIES: TIGR03557 family F420-dependent LLM class oxidoreductase
55(1)   WP_147916372.1 WP_147916372.1 TIGR03842 family LLM class F420-dependent oxidoreductase
55(1)   WP_028472261.1 WP_028472261.1 LLM class flavin-dependent oxidoreductase
55(1)   WP_252619907.1 WP_252619907.1 LLM class F420-dependent oxidoreductase
55(1)   WP_147916377.1 WP_147916377.1 LLM class flavin-dependent oxidoreductase
55(1)   WP_056917801.1 WP_056917801.1 LLM class F420-dependent oxidoreductase

56(1)   WP_174181826.1 WP_174181826.1 serine O-acetyltransferase EpsC
56(1)   WP_126988463.1 WP_126988463.1 MULTISPECIES: sugar O-acetyltransferase
56(1)   WP_038990962.1 WP_038990962.1 MULTISPECIES: serine O-acetyltransferase EpsC
56(1)   WP_252624176.1 WP_252624176.1 sugar O-acetyltransferase
56(1)   WP_205654076.1 WP_205654076.1 serine O-acetyltransferase EpsC
56(1)   WP_147916389.1 WP_147916389.1 DapH/DapD/GlmU-related protein

57(1)   WP_070320441.1 WP_070320441.1 hypothetical protein

57(1)   WP_170210866.1 WP_170210866.1 MULTISPECIES: hypothetical protein
57(1)   WP_203670383.1 WP_203670383.1 hypothetical protein
57(1)   WP_029289525.1 WP_029289525.1 hypothetical protein
57(1)   WP_259307156.1 WP_259307156.1 hypothetical protein
57(1)   WP_199423942.1 WP_199423942.1 hypothetical protein

58(1)   WP_056153715.1 WP_056153715.1 MULTISPECIES: MauE/DoxX family redox-associated membrane protein
58(1)   WP_278175644.1 WP_278175644.1 MauE/DoxX family redox-associated membrane protein
58(1)   WP_213011517.1 WP_213011517.1 MauE/DoxX family redox-associated membrane protein
58(1)   WP_091063595.1 WP_091063595.1 MauE/DoxX family redox-associated membrane protein
58(1)   WP_263021845.1 WP_263021845.1 MauE/DoxX family redox-associated membrane protein
58(1)   WP_148240392.1 WP_148240392.1 MauE/DoxX family redox-associated membrane protein

59(1)   WP_147796141.1 WP_147796141.1 glycine betaine ABC transporter substrate-binding protein
59(1)   WP_046528005.1 WP_046528005.1 MULTISPECIES: glycine betaine ABC transporter substrate-binding protein
59(1)   WP_283320827.1 WP_283320827.1 glycine betaine ABC transporter substrate-binding protein
59(1)   WP_029289537.1 WP_029289537.1 glycine betaine ABC transporter substrate-binding protein
59(1)   WP_259307163.1 WP_259307163.1 glycine betaine ABC transporter substrate-binding protein
59(1)   WP_203670378.1 WP_203670378.1 glycine betaine ABC transporter substrate-binding protein

60(1)   WP_046528003.1 WP_046528003.1 MULTISPECIES: hypothetical protein
60(1)   WP_259307167.1 WP_259307167.1 hypothetical protein
60(1)   WP_203670376.1 WP_203670376.1 hypothetical protein
60(1)   WP_147796136.1 WP_147796136.1 hypothetical protein
60(1)   WP_283320831.1 WP_283320831.1 hypothetical protein

60(1)   WP_199423946.1 WP_199423946.1 hypothetical protein

61(1)   WP_308738568.1 WP_308738568.1 alcohol dehydrogenase catalytic domain-containing protein
61(1)   WP_150242306.1 WP_150242306.1 NADP-dependent oxidoreductase
61(1)   WP_252619924.1 WP_252619924.1 L-threonine 3-dehydrogenase
61(1)   WP_212321628.1 WP_212321628.1 zinc-dependent alcohol dehydrogenase family protein
61(1)   WP_014375117.1 WP_014375117.1 NADPH:quinone reductase

61(1)   WP_213011487.1 WP_213011487.1 zinc-binding dehydrogenase

62(1)   WP_128470349.1 WP_128470349.1 glyceraldehyde-3-phosphate dehydrogenase
62(1)   WP_229777309.1 WP_229777309.1 glyceraldehyde-3-phosphate dehydrogenase
62(1)   WP_205654070.1 WP_205654070.1 glyceraldehyde-3-phosphate dehydrogenase
62(1)   WP_096288801.1 WP_096288801.1 MULTISPECIES: glyceraldehyde-3-phosphate dehydrogenase
62(1)   WP_212321592.1 WP_212321592.1 type I glyceraldehyde-3-phosphate dehydrogenase
62(1)   WP_310112496.1 WP_310112496.1 glyceraldehyde-3-phosphate dehydrogenase

63(1)   WP_149378834.1 WP_149378834.1 site-specific integrase

63(1)   WP_223830497.1 WP_223830497.1 site-specific integrase

63(1)   WP_283663407.1 WP_283663407.1 tyrosine-type recombinase/integrase
63(1)   WP_082581073.1 WP_082581073.1 tyrosine-type recombinase/integrase
63(1)   WP_326338221.1 WP_326338221.1 tyrosine-type recombinase/integrase
63(1)   WP_131167287.1 WP_131167287.1 site-specific integrase

64(1)   WP_043642044.1 WP_043642044.1 glycosyltransferase
64(1)   WP_180903512.1 WP_180903512.1 glycosyltransferase
64(1)   WP_052336382.1 WP_052336382.1 glycosyltransferase family 4 protein
64(1)   WP_101787130.1 WP_101787130.1 glycosyltransferase
64(1)   WP_101787126.1 WP_101787126.1 glycosyltransferase

65(1)   WP_003880044.1 WP_003880044.1 MULTISPECIES: holo-ACP synthase
65(1)   WP_109148413.1 WP_109148413.1 holo-ACP synthase
65(1)   WP_138842542.1 WP_138842542.1 holo-ACP synthase
65(1)   WP_184443610.1 WP_184443610.1 holo-ACP synthase
65(1)   WP_069398824.1 WP_069398824.1 holo-ACP synthase

66(1)   WP_069398836.1 WP_069398836.1 MmpS family transport accessory protein
66(1)   WP_149378848.1 WP_149378848.1 MmpS family transport accessory protein
66(1)   WP_003880028.1 WP_003880028.1 MULTISPECIES: MmpS family transport accessory protein
66(1)   WP_082972523.1 WP_082972523.1 MULTISPECIES: MmpS family transport accessory protein
66(1)   WP_184443622.1 WP_184443622.1 MmpS family transport accessory protein

67(1)   WP_149378855.1 WP_149378855.1 CoA ester lyase
67(1)   WP_184443630.1 WP_184443630.1 CoA ester lyase
67(1)   WP_003880020.1 WP_003880020.1 CoA ester lyase
67(1)   WP_069398844.1 WP_069398844.1 CoA ester lyase
67(1)   WP_066860314.1 WP_066860314.1 MULTISPECIES: CoA ester lyase

68(1)   WP_048342600.1 WP_048342600.1 MULTISPECIES: thioredoxin
68(1)   WP_062103346.1 WP_062103346.1 MULTISPECIES:

thioredoxin
68(1)   WP_199423950.1 WP_199423950.1 thioredoxin
68(1)   WP_203670372.1 WP_203670372.1 thioredoxin
68(1)   WP_029289545.1 WP_029289545.1 thioredoxin

69(1)   WP_174181846.1 WP_174181846.1 MULTISPECIES: peptide deformylase
69(1)   WP_105552062.1 WP_105552062.1 MULTISPECIES: peptide deformylase
69(1)   WP_188947949.1 WP_188947949.1 peptide deformylase
69(1)   WP_205654067.1 WP_205654067.1 peptide deformylase
69(1)   WP_128470346.1 WP_128470346.1 peptide deformylase

70(1)   WP_067178794.1 WP_067178794.1 MaoC family dehydratase

70(1)   WP_184443629.1 WP_184443629.1 MaoC family dehydratase

70(1)   WP_003880021.1 WP_003880021.1 MULTISPECIES: MaoC family dehydratase
70(1)   WP_232326899.1 WP_232326899.1 MaoC family dehydratase

70(1)   WP_149378854.1 WP_149378854.1 MaoC family dehydratase

71(1)   WP_254584108.1 WP_254584108.1 type I polyketide synthase
71(1)   WP_069398884.1 WP_069398884.1 type I polyketide synthase
71(1)   WP_065148205.1 WP_065148205.1 type I polyketide synthase
71(1)   WP_184443609.1 WP_184443609.1 type I polyketide synthase
71(1)   WP_259843079.1 WP_259843079.1 type I polyketide synthase

72(1)   WP_213011483.1 WP_213011483.1 copper resistance protein CopC
72(1)   WP_278181958.1 WP_278181958.1 copper resistance

protein CopC
72(1)   WP_263021847.1 WP_263021847.1 copper resistance protein CopC
72(1)   WP_175441350.1 WP_175441350.1 copper resistance protein CopC
72(1)   WP_056917811.1 WP_056917811.1 copper resistance protein CopC

73(1)   WP_110047913.1 WP_110047913.1 histidine kinase
73(1)   WP_185973089.1 WP_185973089.1 histidine kinase
73(1)   WP_235530799.1 WP_235530799.1 histidine kinase
73(1)   WP_199423937.1 WP_199423937.1 sensor domain-containing protein
73(1)   WP_308738552.1 WP_308738552.1 histidine kinase

74(1)   WP_205654072.1 WP_205654072.1 peroxide stress protein YaaA
74(1)   WP_105569280.1 WP_105569280.1 MULTISPECIES: peroxide stress protein YaaA
74(1)   WP_320965251.1 WP_320965251.1 peroxide stress protein YaaA
74(1)   WP_128470477.1 WP_128470477.1 peroxide stress protein YaaA
74(1)   WP_310112493.1 WP_310112493.1 peroxide stress protein YaaA

75(1)   WP_326338224.1 WP_326338224.1 RNA methyltransferase
75(1)   WP_110047907.1 WP_110047907.1 RNA methyltransferase
75(1)   WP_132049016.1 WP_132049016.1 RNA methyltransferase
75(1)   WP_148757148.1 WP_148757148.1 RNA methyltransferase
75(1)   WP_150242286.1 WP_150242286.1 RNA methyltransferase

76(1)   WP_201049332.1 WP_201049332.1 serine/threonine protein kinase
76(1)   WP_329243473.1 WP_329243473.1 serine/threonine protein kinase
76(1)   WP_059205165.1 WP_059205165.1 MULTISPECIES: serine/threonine protein kinase

76(2) WP_266759068.1 WP_266759068.1 MULTISPECIES: serine/threonine protein kinase

77(1) WP_330345286.1 WP_330345286.1 GNAT family N-acetyltransferase
77(2) WP_266759066.1 WP_266759066.1 MULTISPECIES: GNAT family N-acetyltransferase
77(1) WP_201049331.1 WP_201049331.1 GNAT family N-acetyltransferase
77(1) WP_266514963.1 WP_266514963.1 MULTISPECIES: GNAT family N-acetyltransferase

78(1) WP_147916369.1 WP_147916369.1 FAD-dependent monooxygenase
78(1) WP_069742614.1 WP_069742614.1 MULTISPECIES: FAD-dependent monooxygenase
78(1) WP_150242310.1 WP_150242310.1 NAD(P)/FAD-dependent oxidoreductase
78(1) WP_189158425.1 WP_189158425.1 rifampin monooxygenase

79(1) WP_150242338.1 WP_150242338.1 DMT family transporter
79(1) WP_199560511.1 WP_199560511.1 DMT family transporter
79(1) WP_202423228.1 WP_202423228.1 MULTISPECIES: DMT family transporter
79(1) WP_067178804.1 WP_067178804.1 DMT family transporter

80(1) WP_203670371.1 WP_203670371.1 NUDIX hydrolase family protein
80(1) WP_029289547.1 WP_029289547.1 NUDIX hydrolase family protein
80(1) WP_199423951.1 WP_199423951.1 NUDIX hydrolase family protein
80(1) WP_048342599.1 WP_048342599.1 MULTISPECIES: NUDIX hydrolase family protein

81(1) WP_205654075.1 WP_205654075.1 cysteine synthase A
81(1) WP_102975900.1 WP_102975900.1 MULTISPECIES: cysteine

synthase A
81(1)   WP_038990964.1 WP_038990964.1 MULTISPECIES: cysteine synthase A
81(1)   WP_105569101.1 WP_105569101.1 MULTISPECIES: cysteine synthase A

82(1)   WP_210648825.1 WP_210648825.1 NAD(P)/FAD-dependent oxidoreductase
82(1)   WP_148240391.1 WP_148240391.1 NAD(P)/FAD-dependent oxidoreductase
82(1)   WP_056153675.1 WP_056153675.1 MULTISPECIES: NAD(P)/FAD-dependent oxidoreductase
82(1)   WP_052336384.1 WP_052336384.1 NAD(P)/FAD-dependent oxidoreductase

83(1)   WP_221632430.1 WP_221632430.1 fluoride efflux transporter CrcB
83(1)   WP_141787601.1 WP_141787601.1 CrcB family protein
83(1)   WP_147916383.1 WP_147916383.1 CrcB family protein
83(1)   WP_147916382.1 WP_147916382.1 CrcB family protein

84(1)   WP_148757156.1 WP_148757156.1 phosphotransferase family protein
84(1)   WP_132049024.1 WP_132049024.1 phosphotransferase family protein
84(1)   WP_250357387.1 WP_250357387.1 phosphotransferase family protein
84(1)   WP_101787143.1 WP_101787143.1 phosphotransferase family protein

85(1)   WP_254584110.1 WP_254584110.1 dipeptidase
85(1)   WP_184443611.1 WP_184443611.1 dipeptidase
85(1)   WP_065148206.1 WP_065148206.1 dipeptidase
85(1)   WP_069398825.1 WP_069398825.1 dipeptidase

86(1)   WP_255268932.1 WP_255268932.1 pyridoxamine 5'-phosphate oxidase family protein

86(1)   WP_101787142.1 WP_101787142.1 pyridoxamine 5'-phosphate oxidase family protein
86(1)   WP_132049028.1 WP_132049028.1 pyridoxamine 5'-phosphate oxidase family protein
86(1)   WP_148757158.1 WP_148757158.1 pyridoxamine 5'-phosphate oxidase family protein

87(1)   WP_066860300.1 WP_066860300.1 MULTISPECIES: enoyl-CoA hydratase
87(1)   WP_255279856.1 WP_255279856.1 enoyl-CoA hydratase-related protein
87(1)   WP_003880016.1 WP_003880016.1 enoyl-CoA hydratase
87(1)   WP_069398847.1 WP_069398847.1 enoyl-CoA hydratase

88(1)   WP_259809477.1 WP_259809477.1 Crp/Fnr family transcriptional regulator
88(1)   WP_048342605.1 WP_048342605.1 MULTISPECIES: Crp/Fnr family transcriptional regulator
88(1)   WP_045879973.1 WP_045879973.1 Crp/Fnr family transcriptional regulator
88(1)   WP_251746091.1 WP_251746091.1 Crp/Fnr family transcriptional regulator

89(1)   WP_246607918.1 WP_246607918.1 RICIN domain-containing protein
89(1)   WP_091063634.1 WP_091063634.1 RICIN domain-containing protein
89(1)   WP_263021860.1 WP_263021860.1 hypothetical protein
89(1)   WP_246607919.1 WP_246607919.1 pectinesterase family protein

90(1)   WP_097193153.1 WP_097193153.1 MMPL family transporter

90(1)   WP_067178806.1 WP_067178806.1 MMPL family transporter

90(2)   WP_266759080.1 WP_266759080.1 MULTISPECIES: MMPL family transporter

91(1)   WP_246607922.1 WP_246607922.1 hypothetical protein
91(1)   WP_278175647.1 WP_278175647.1 hypothetical protein
91(1)   WP_091064413.1 WP_091064413.1 hypothetical protein
91(1)   WP_263021843.1 WP_263021843.1 hypothetical protein

92(1)   WP_091063584.1 WP_091063584.1 cobalt ECF transporter T component CbiQ
92(1)   WP_138875248.1 WP_138875248.1 cobalt ECF transporter T component CbiQ
92(1)   WP_210648832.1 WP_210648832.1 cobalt ECF transporter T component CbiQ
92(1)   WP_278175650.1 WP_278175650.1 cobalt ECF transporter T component CbiQ

93(1)   WP_210649822.1 WP_210649822.1 PDGLE domain-containing protein
93(1)   WP_187366826.1 WP_187366826.1 PDGLE domain-containing protein
93(1)   WP_245716411.1 WP_245716411.1 PDGLE domain-containing protein
93(1)   WP_278175652.1 WP_278175652.1 PDGLE domain-containing protein

94(1)   WP_147796148.1 WP_147796148.1 MarR family transcriptional regulator
94(1)   WP_029110079.1 WP_029110079.1 MarR family transcriptional regulator
94(1)   WP_137292552.1 WP_137292552.1 MULTISPECIES: MarR family transcriptional regulator
94(1)   WP_283320818.1 WP_283320818.1 MarR family transcriptional regulator

95(1)   WP_283320819.1 WP_283320819.1 NAD(P)-binding domain-containing protein
95(1)   WP_029110085.1 WP_029110085.1 NAD(P)-binding domain-containing protein
95(1)   WP_147796147.1 WP_147796147.1 NAD(P)-binding

domain-containing protein
95(1)   WP_029110080.1 WP_029110080.1 NAD(P)-binding domain-containing protein

96(1)   WP_243718611.1 WP_243718611.1 PLP-dependent aminotransferase family protein
96(1)   WP_246178416.1 WP_246178416.1 PLP-dependent aminotransferase family protein
96(1)   WP_245899233.1 WP_245899233.1 PLP-dependent aminotransferase family protein
96(1)   WP_308207693.1 WP_308207693.1 hypothetical protein

97(1)   WP_308738560.1 WP_308738560.1 NADH:flavin oxidoreductase/NADH oxidase
97(1)   WP_063060741.1 WP_063060741.1 NADPH-dependent 2,4-dienoyl-CoA reductase
97(1)   WP_102030792.1 WP_102030792.1 MULTISPECIES: NADPH-dependent 2,4-dienoyl-CoA reductase
97(1)   WP_259813889.1 WP_259813889.1 NADH:flavin oxidoreductase/NADH oxidase

98(1)   WP_259813892.1 WP_259813892.1 NAD(P)-dependent oxidoreductase
98(1)   WP_101787132.1 WP_101787132.1 GDP-mannose 4,6-dehydratase
98(1)   WP_101787131.1 WP_101787131.1 NAD-dependent epimerase/dehydratase family protein
98(1)   WP_308738562.1 WP_308738562.1 NAD(P)-dependent oxidoreductase

99(1)   WP_128470351.1 WP_128470351.1 Nif3-like dinuclear metal center hexameric protein
99(1)   WP_105569100.1 WP_105569100.1 MULTISPECIES: Nif3-like dinuclear metal center hexameric protein
99(1)   WP_310112490.1 WP_310112490.1 Nif3-like dinuclear metal center hexameric protein
99(1)   WP_205654116.1 WP_205654116.1 Nif3-like dinuclear metal center hexameric protein

100(1) WP_101787141.1 WP_101787141.1 serine/threonine-protein kinase
100(1) WP_076406678.1 WP_076406678.1 MULTISPECIES: serine/threonine-protein kinase
100(1) WP_219601860.1 WP_219601860.1 protein kinase
100(1) WP_320965250.1 WP_320965250.1 phosphotransferase

101(1) WP_329243470.1 WP_329243470.1 SDR family oxidoreductase
101(1) WP_059205166.1 WP_059205166.1 MULTISPECIES: SDR family oxidoreductase
101(1) WP_029110081.1 WP_029110081.1 SDR family oxidoreductase
101(1) WP_280898538.1 WP_280898538.1 SDR family oxidoreductase

102(1) WP_329243482.1 WP_329243482.1 beta-galactosidase
102(1) WP_201049339.1 WP_201049339.1 beta-galactosidase
102(1) WP_326780451.1 WP_326780451.1 beta-galactosidase
102(1) WP_059205162.1 WP_059205162.1 MULTISPECIES: beta-galactosidase

103(1) WP_063060734.1 WP_063060734.1 hypothetical protein
103(1) WP_006554382.1 WP_006554382.1 MULTISPECIES: hypothetical protein
103(1) WP_006939021.1 WP_006939021.1 MULTISPECIES: hypothetical protein

104(1) WP_056917815.1 WP_056917815.1 helix-turn-helix domain-containing protein
104(1) WP_132049032.1 WP_132049032.1 helix-turn-helix domain-containing protein
104(1) WP_069742607.1 WP_069742607.1 MULTISPECIES: helix-turn-helix domain-containing protein

105(1) WP_143913335.1 WP_143913335.1 ABC transporter permease
105(1) WP_110047915.1 WP_110047915.1 FtsX-like permease family protein
105(1) WP_143913336.1 WP_143913336.1 FtsX-like permease family protein

106(1) WP_150242284.1 WP_150242284.1 2-hydroxyacid dehydrogenase
106(1) WP_110047906.1 WP_110047906.1 2-hydroxyacid dehydrogenase
106(1) WP_014911568.1 WP_014911568.1 MULTISPECIES: 2-hydroxyacid dehydrogenase

107(1) WP_110047908.1 WP_110047908.1 hypothetical protein
107(1) WP_150242288.1 WP_150242288.1 hypothetical protein
107(1) WP_014912335.1 WP_014912335.1 MULTISPECIES: hypothetical protein

108(1) WP_045877969.1 WP_045877969.1 aldo/keto reductase
108(1) WP_154347594.1 WP_154347594.1 aldo/keto reductase
108(1) WP_028472272.1 WP_028472272.1 aldo/keto reductase

109(1) WP_147916381.1 WP_147916381.1 DUF1697 domain-containing protein
109(1) WP_045877959.1 WP_045877959.1 DUF1697 domain-containing protein
109(1) WP_156742038.1 WP_156742038.1 DUF1697 domain-containing protein

110(1) WP_161111453.1 WP_161111453.1 alanine racemase
110(1) WP_150242274.1 WP_150242274.1 alanine racemase
110(1) WP_110047900.1 WP_110047900.1 alanine racemase

111(1) WP_097193145.1 WP_097193145.1 hypothetical protein
111(1) WP_166527904.1 WP_166527904.1 hypothetical protein

111(1) WP_014375110.1 WP_014375110.1 hypothetical protein

112(1) WP_185277606.1 WP_185277606.1 spore germination protein GerW family protein
112(1) WP_047523330.1 WP_047523330.1 MULTISPECIES: hypothetical protein
112(1) WP_154347602.1 WP_154347602.1 hypothetical protein

113(1) WP_188950311.1 WP_188950311.1 RelA/SpoT domain-containing protein
113(1) WP_096288783.1 WP_096288783.1 MULTISPECIES: (p)ppGpp synthetase
113(1) WP_066138183.1 WP_066138183.1 MULTISPECIES: (p)ppGpp synthetase

114(1) WP_029535365.1 WP_029535365.1 hypothetical protein
114(1) WP_185002021.1 WP_185002021.1 MULTISPECIES: hypothetical protein
114(1) WP_189158430.1 WP_189158430.1 hypothetical protein

115(1) WP_190394354.1 WP_190394354.1 C40 family peptidase
115(1) WP_017534252.1 WP_017534252.1 MULTISPECIES: C40 family peptidase
115(1) WP_110047904.1 WP_110047904.1 C40 family peptidase

116(1) WP_065148212.1 WP_065148212.1 pyruvate dehydrogenase (acetyl-transferring) E1 component subunit alpha
116(1) WP_149378856.1 WP_149378856.1 pyruvate dehydrogenase (acetyl-transferring) E1 component subunit alpha
116(1) WP_197497860.1 WP_197497860.1 MULTISPECIES: pyruvate dehydrogenase (acetyl-transferring) E1 component subunit alpha

117(1) WP_199423940.1 WP_199423940.1 phosphatase PAP2 family protein
117(1) WP_051681779.1 WP_051681779.1 phosphatase PAP2 family

protein
117(1) WP_144680236.1 WP_144680236.1 MULTISPECIES: phosphatase PAP2 family protein

118(1) WP_029289551.1 WP_029289551.1 RdgB/HAM1 family non-canonical purine NTP pyrophosphatase
118(1) WP_048342597.1 WP_048342597.1 MULTISPECIES: RdgB/HAM1 family non-canonical purine NTP pyrophosphatase
118(1) WP_199423953.1 WP_199423953.1 RdgB/HAM1 family non-canonical purine NTP pyrophosphatase

119(1) WP_141368736.1 WP_141368736.1 SDR family oxidoreductase
119(1) WP_203670395.1 WP_203670395.1 SDR family oxidoreductase
119(1) WP_070319957.1 WP_070319957.1 SDR family oxidoreductase

120(1) WP_204677330.1 WP_204677330.1 DUF2637 domain-containing protein
120(1) WP_096288785.1 WP_096288785.1 MULTISPECIES: DUF2637 domain-containing protein
120(1) WP_052253742.1 WP_052253742.1 MULTISPECIES: DUF2637 domain-containing protein

121(1) WP_161567471.1 WP_161567471.1 DUF3099 domain-containing protein
121(1) WP_105569095.1 WP_105569095.1 MULTISPECIES: DUF3099 domain-containing protein
121(1) WP_204677334.1 WP_204677334.1 DUF3099 domain-containing protein

122(1) WP_210648819.1 WP_210648819.1 adenosine deaminase
122(1) WP_056153720.1 WP_056153720.1 MULTISPECIES: adenosine deaminase
122(1) WP_222847671.1 WP_222847671.1 adenosine deaminase

123(1) WP_246910444.1 WP_246910444.1 ABC transporter substrate-binding protein
123(1) WP_086151439.1 WP_086151439.1 MULTISPECIES: ABC transporter substrate-binding protein
123(1) WP_131167298.1 WP_131167298.1 ABC transporter substrate-binding protein

124(1) WP_144680226.1 WP_144680226.1 biotin transporter BioY

124(1) WP_246910447.1 WP_246910447.1 biotin transporter BioY

124(1) WP_223927488.1 WP_223927488.1 biotin transporter BioY

125(1) WP_246910456.1 WP_246910456.1 energy-coupling factor transporter transmembrane component T
125(1) WP_212321600.1 WP_212321600.1 energy-coupling factor transporter transmembrane component T
125(1) WP_144680230.1 WP_144680230.1 energy-coupling factor transporter transmembrane protein EcfT

126(1) WP_154347588.1 WP_154347588.1 hypothetical protein
126(1) WP_252619904.1 WP_252619904.1 hypothetical protein
126(1) WP_134353684.1 WP_134353684.1 hypothetical protein

127(1) WP_254584114.1 WP_254584114.1 SGNH/GDSL hydrolase family protein
127(1) WP_040271737.1 WP_040271737.1 MULTISPECIES: SGNH/GDSL hydrolase family protein
127(1) WP_252619926.1 WP_252619926.1 SGNH/GDSL hydrolase family protein

128(1) WP_010596350.1 WP_010596350.1 MULTISPECIES: FAD-binding oxidoreductase
128(1) WP_063060736.1 WP_063060736.1 FAD-binding oxidoreductase

128(1) WP_254584116.1 WP_254584116.1 ferredoxin reductase

129(1) WP_254584117.1 WP_254584117.1 acyl-CoA desaturase
129(1) WP_063060735.1 WP_063060735.1 acyl-CoA desaturase
129(1) WP_010596351.1 WP_010596351.1 MULTISPECIES: acyl-CoA desaturase

130(1) WP_246910452.1 WP_246910452.1 beta-ketoacyl synthase N-terminal-like domain-containing protein
130(1) WP_255434383.1 WP_255434383.1 thiolase family protein

130(1) WP_138875257.1 WP_138875257.1 acetyl-CoA C-acetyltransferase

131(1) WP_134353677.1 WP_134353677.1 hypothetical protein
131(1) WP_154347600.1 WP_154347600.1 ATP-binding protein
131(1) WP_258046106.1 WP_258046106.1 ATP-binding protein

132(1) WP_045877960.1 WP_045877960.1 hypothetical protein
132(1) WP_251746085.1 WP_251746085.1 hypothetical protein
132(1) WP_261559526.1 WP_261559526.1 hypothetical protein, partial

133(1) WP_064257051.1 WP_064257051.1 MULTISPECIES: HNH endonuclease signature motif containing protein
133(1) WP_217628623.1 WP_217628623.1 HNH endonuclease
133(1) WP_263021841.1 WP_263021841.1 helix-turn-helix domain-containing protein

134(1) WP_199423948.1 WP_199423948.1 ATP-binding protein
134(2) WP_266759082.1 WP_266759082.1 MULTISPECIES: ATP-binding protein

135(1) WP_006131567.1 WP_006131567.1 MULTISPECIES: hypothetical protein

135(1) WP_266803687.1 WP_266803687.1 hypothetical protein
135(1) WP_266514981.1 WP_266514981.1 MULTISPECIES: hypothetical protein

136(1) WP_278175627.1 WP_278175627.1 glycosyltransferase
136(1) WP_278175628.1 WP_278175628.1 glycosyltransferase
136(1) WP_278175629.1 WP_278175629.1 glycosyltransferase

137(1) WP_091063581.1 WP_091063581.1 energy-coupling factor ABC transporter permease
137(1) WP_278175653.1 WP_278175653.1 energy-coupling factor ABC transporter permease
137(1) WP_187366825.1 WP_187366825.1 energy-coupling factor ABC transporter permease

138(1) WP_266383322.1 WP_266383322.1 glycoside hydrolase family 36 protein
138(1) WP_059205161.1 WP_059205161.1 MULTISPECIES: glycoside hydrolase family 36 protein
138(1) WP_280851327.1 WP_280851327.1 alpha-galactosidase

139(1) WP_056153740.1 WP_056153740.1 MULTISPECIES: PGPGW domain-containing protein
139(1) WP_283320817.1 WP_283320817.1 PGPGW domain-containing protein
139(1) WP_147796149.1 WP_147796149.1 PGPGW domain-containing protein

140(1) WP_065148213.1 WP_065148213.1 alpha-ketoacid dehydrogenase subunit beta
140(1) WP_286199268.1 WP_286199268.1 alpha-ketoacid dehydrogenase subunit beta
140(1) WP_067178792.1 WP_067178792.1 3-methyl-2-oxobutanoate dehydrogenase subunit beta

141(1) WP_262347581.1 WP_262347581.1 aminopeptidase P family

protein
141(1) WP_307861804.1 WP_307861804.1 aminopeptidase P family protein
141(1) WP_056153745.1 WP_056153745.1 MULTISPECIES: aminopeptidase P family protein

142(1) WP_308738545.1 WP_308738545.1 DNA-formamidopyrimidine glycosylase family protein
142(1) WP_010549695.1 WP_010549695.1 DNA-formamidopyrimidine glycosylase family protein
142(1) WP_131490989.1 WP_131490989.1 MULTISPECIES: DNA-formamidopyrimidine glycosylase family protein

143(1) WP_097193150.1 WP_097193150.1 metal-sensitive transcriptional regulator
143(1) WP_129459419.1 WP_129459419.1 MULTISPECIES: metal-sensitive transcriptional regulator
143(1) WP_308738566.1 WP_308738566.1 metal-sensitive transcriptional regulator

144(1) WP_312007928.1 WP_312007928.1 fused MFS/spermidine synthase
144(1) WP_031017385.1 WP_031017385.1 MULTISPECIES: fused MFS/spermidine synthase
144(1) WP_266514983.1 WP_266514983.1 MULTISPECIES: fused MFS/spermidine synthase

145(1) WP_318603918.1 WP_318603918.1 iron chelate uptake ABC transporter family permease subunit
145(1) WP_156742032.1 WP_156742032.1 iron ABC transporter permease
145(1) WP_156742031.1 WP_156742031.1 iron chelate uptake ABC transporter family permease subunit

146(1) WP_320965257.1 WP_320965257.1 winged helix DNA-binding domain-containing protein
146(1) WP_128470347.1 WP_128470347.1 winged helix

DNA-binding domain-containing protein
146(1) WP_105569097.1 WP_105569097.1 MULTISPECIES: winged helix DNA-binding domain-containing protein

147(1) WP_203670385.1 WP_203670385.1 DUF2079 domain-containing protein
147(1) WP_070320853.1 WP_070320853.1 DUF2079 domain-containing protein
147(1) WP_320965263.1 WP_320965263.1 DUF2079 domain-containing protein

148(1) WP_128470341.1 WP_128470341.1 VanZ family protein
148(1) WP_320965264.1 WP_320965264.1 VanZ family protein
148(1) WP_105569096.1 WP_105569096.1 VanZ family protein

149(1) WP_105552067.1 WP_105552067.1 MULTISPECIES: SURF1 family protein
149(1) WP_320965267.1 WP_320965267.1 SURF1 family protein
149(1) WP_128470338.1 WP_128470338.1 SURF1 family protein

150(1) WP_128470337.1 WP_128470337.1 acetone carboxylase
150(1) WP_320965268.1 WP_320965268.1 acetone carboxylase
150(1) WP_105552068.1 WP_105552068.1 MULTISPECIES: acetone carboxylase

151(1) WP_259307153.1 WP_259307153.1 globin
151(1) WP_131490984.1 WP_131490984.1 MULTISPECIES: globin
151(1) WP_324613567.1 WP_324613567.1 globin

152(1) WP_185278772.1 WP_185278772.1 metallopeptidase family protein
152(1) WP_324613569.1 WP_324613569.1 metallopeptidase family protein
152(1) WP_134353680.1 WP_134353680.1 metallopeptidase family protein

153(1) WP_134353676.1 WP_134353676.1 DNA polymerase IV
153(1) WP_258046107.1 WP_258046107.1 DNA polymerase IV
153(1) WP_324613572.1 WP_324613572.1 DNA polymerase IV

154(1) WP_047523334.1 WP_047523334.1 MULTISPECIES: Asp-tRNA(Asn)/Glu-tRNA(Gln) amidotransferase subunit GatA
154(1) WP_324613573.1 WP_324613573.1 Asp-tRNA(Asn)/Glu-tRNA(Gln) amidotransferase subunit GatA
154(1) WP_246861438.1 WP_246861438.1 amidase family protein

155(1) WP_223830496.1 WP_223830496.1 hypothetical protein
155(1) WP_110047911.1 WP_110047911.1 DUF2510 domain-containing protein
155(1) WP_326338222.1 WP_326338222.1 DUF2510 domain-containing protein

156(1) WP_110047903.1 WP_110047903.1 gamma-glutamyltransferase family protein
156(1) WP_150242280.1 WP_150242280.1 gamma-glutamyltransferase family protein
156(1) WP_326338226.1 WP_326338226.1 gamma-glutamyltransferase family protein

157(1) WP_110047902.1 WP_110047902.1 hypothetical protein
157(1) WP_326338227.1 WP_326338227.1 hypothetical protein
157(1) WP_150242278.1 WP_150242278.1 hypothetical protein

158(1) WP_150242276.1 WP_150242276.1 glycerophosphodiester phosphodiesterase family protein
158(1) WP_326338228.1 WP_326338228.1 glycerophosphodiester phosphodiesterase family protein
158(1) WP_110047901.1 WP_110047901.1 glycerophosphodiester phosphodiesterase family protein

159(1) WP_329243489.1 WP_329243489.1 carbohydrate ABC

transporter substrate-binding protein
159(1) WP_280898543.1 WP_280898543.1 extracellular solute-binding protein
159(1) WP_330345285.1 WP_330345285.1 extracellular solute-binding protein

160(1) WP_006554758.1 WP_006554758.1 MULTISPECIES: TetR family transcriptional regulator
160(1) WP_007626718.1 WP_007626718.1 MULTISPECIES: TetR/AcrR family transcriptional regulator

161(1) WP_014375098.1 WP_014375098.1 precorrin-2 C(20)-methyltransferase
161(1) WP_014375094.1 WP_014375094.1 precorrin-4 C(11)-methyltransferase

162(1) WP_029110076.1 WP_029110076.1 DUF899 family protein
162(1) WP_003880036.1 WP_003880036.1 MULTISPECIES: DUF899 family protein

163(1) WP_029110074.1 WP_029110074.1 nuclear transport factor 2 family protein
163(1) WP_065148207.1 WP_065148207.1 nuclear transport factor 2 family protein

164(1) WP_065148208.1 WP_065148208.1 ATPase
164(1) WP_029110075.1 WP_029110075.1 ATPase

165(1) WP_065148209.1 WP_065148209.1 PPE family protein
165(1) WP_029110078.1 WP_029110078.1 PPE family protein

166(1) WP_003880017.1 WP_003880017.1 dihydrolipoamide acetyltransferase family protein
166(1) WP_066860303.1 WP_066860303.1 MULTISPECIES: dihydrolipoamide acetyltransferase family protein

167(1) WP_067178808.1 WP_067178808.1 DUF3556 domain-containing protein
167(1) WP_069398829.1 WP_069398829.1 DUF3556 domain-containing protein

168(1) WP_063060729.1 WP_063060729.1 cytochrome c oxidase assembly protein
168(1) WP_070721260.1 WP_070721260.1 MULTISPECIES: cytochrome c oxidase assembly protein

169(2) WP_093739039.1 WP_093739039.1 MULTISPECIES: class F sortase

170(2) WP_093739053.1 WP_093739053.1 MULTISPECIES: Lrp/AsnC family transcriptional regulator

171(2) WP_093739057.1 WP_093739057.1 MULTISPECIES: discoidin domain-containing protein

172(2) WP_093739059.1 WP_093739059.1 MULTISPECIES: hypothetical protein

173(2) WP_093739134.1 WP_093739134.1 MULTISPECIES: PaaI family thioesterase

174(1) WP_014375121.1 WP_014375121.1 cupin domain-containing protein
174(1) WP_097193137.1 WP_097193137.1 cupin domain-containing protein

175(1) WP_014375119.1 WP_014375119.1 hypothetical protein
175(1) WP_097193138.1 WP_097193138.1 hypothetical protein

176(1) WP_097193155.1 WP_097193155.1 cytochrome P450
176(1) WP_010549711.1 WP_010549711.1 MULTISPECIES: cytochrome P450

177(1) WP_028472431.1 WP_028472431.1 low molecular weight phosphatase family protein
177(1) WP_101787135.1 WP_101787135.1 hypothetical protein

178(1) WP_079134125.1 WP_079134125.1 FAD-binding oxidoreductase
178(1) WP_107403682.1 WP_107403682.1 FAD-binding oxidoreductase

179(1) WP_126988465.1 WP_126988465.1 MULTISPECIES: thiamine-binding protein
179(1) WP_053918099.1 WP_053918099.1 MULTISPECIES: thiamine-binding protein

180(1) WP_097193148.1 WP_097193148.1 DUF305 domain-containing protein
180(1) WP_131167288.1 WP_131167288.1 DUF305 domain-containing protein

181(2) WP_141708933.1 WP_141708933.1 MULTISPECIES: hypothetical protein

182(1) WP_141787597.1 WP_141787597.1 GMC family oxidoreductase
182(1) WP_045877953.1 WP_045877953.1 GMC oxidoreductase

183(1) WP_144680240.1 WP_144680240.1 LysE family translocator
183(1) WP_134353681.1 WP_134353681.1 LysE family transporter

184(1) WP_147916374.1 WP_147916374.1 nitrilase-related carbon-nitrogen hydrolase
184(1) WP_052336378.1 WP_052336378.1 nitrilase family protein

185(1) WP_148757179.1 WP_148757179.1 dihydrofolate reductase family protein
185(1) WP_014375100.1 WP_014375100.1 dihydrofolate reductase family protein

186(1) WP_082574692.1 WP_082574692.1 MULTISPECIES: DUF2231 domain-containing protein
186(1) WP_154347591.1 WP_154347591.1 hypothetical protein

187(1) WP_154347604.1 WP_154347604.1 Asp-tRNA(Asn)/Glu-tRNA(Gln) amidotransferase subunit GatB
187(1) WP_134353675.1 WP_134353675.1 Asp-tRNA(Asn)/Glu-tRNA(Gln) amidotransferase subunit GatB

188(1) WP_154347605.1 WP_154347605.1 MULTISPECIES: Asp-tRNA(Asn)/Glu-tRNA(Gln) amidotransferase subunit GatC
188(1) WP_047523335.1 WP_047523335.1 MULTISPECIES: Asp-tRNA(Asn)/Glu-tRNA(Gln) amidotransferase subunit GatC

189(1) WP_147796155.1 WP_147796155.1 MULTISPECIES: ABC transporter permease
189(1) WP_156742051.1 WP_156742051.1 ABC transporter permease

190(2) WP_161302380.1 WP_161302380.1 MULTISPECIES: acyltransferase domain-containing protein

191(1) WP_147796138.1 WP_147796138.1 methyl-accepting chemotaxis protein
191(1) WP_166527901.1 WP_166527901.1 methyl-accepting chemotaxis protein

192(1) WP_180903516.1 WP_180903516.1 polysaccharide biosynthesis tyrosine autokinase
192(1) WP_052336387.1 WP_052336387.1 polysaccharide biosynthesis tyrosine autokinase

193(1) WP_069398823.1 WP_069398823.1 DUF1906 domain-containing protein
193(1) WP_184443608.1 WP_184443608.1 DUF1906 domain-containing protein

194(1) WP_184443623.1 WP_184443623.1 hypothetical protein
194(1) WP_115320827.1 WP_115320827.1 hypothetical protein

195(1) WP_168214823.1 WP_168214823.1 MULTISPECIES: DUF6131 family protein
195(1) WP_184443624.1 WP_184443624.1 DUF6131 family protein

196(1) WP_154347590.1 WP_154347590.1 Rieske (2Fe-2S) protein

196(1) WP_185277584.1 WP_185277584.1 Rieske (2Fe-2S) protein

197(1) WP_154347596.1 WP_154347596.1 ammonia-dependent NAD(+) synthetase
197(1) WP_185277590.1 WP_185277590.1 ammonia-dependent NAD(+) synthetase

198(1) WP_185277597.1 WP_185277597.1 winged helix-turn-helix domain-containing protein
198(1) WP_144680234.1 WP_144680234.1 MULTISPECIES:

helix-turn-helix domain-containing protein

199(1) WP_154347599.1 WP_154347599.1 hypothetical protein
199(1) WP_185277602.1 WP_185277602.1 hypothetical protein

200(1) WP_185277603.1 WP_185277603.1 energy-coupling factor transporter transmembrane component T
200(1) WP_154347601.1 WP_154347601.1 energy-coupling factor transporter transmembrane component T

201(1) WP_194239225.1 WP_194239225.1 MULTISPECIES: hypothetical protein
201(1) WP_185277587.1 WP_185277587.1 hypothetical protein

202(1) WP_195760731.1 WP_195760731.1 FAD-binding dehydrogenase
202(1) WP_134353685.1 WP_134353685.1 MULTISPECIES: FAD-binding dehydrogenase

203(1) WP_199423954.1 WP_199423954.1 ribonuclease PH
203(1) WP_048342596.1 WP_048342596.1 MULTISPECIES: ribonuclease PH

204(2) WP_202451645.1 WP_202451645.1 MULTISPECIES: inositol monophosphatase family protein

205(1) WP_070319958.1 WP_070319958.1 GNAT family N-acetyltransferase
205(1) WP_203670393.1 WP_203670393.1 GNAT family N-acetyltransferase

206(1) WP_203670396.1 WP_203670396.1 DNA topoisomerase IB
206(1) WP_070319956.1 WP_070319956.1 MULTISPECIES: DNA topoisomerase IB

207(1) WP_174181838.1 WP_174181838.1 MULTISPECIES: hypothetical protein
207(1) WP_205654069.1 WP_205654069.1 hypothetical protein

208(1) WP_174181830.1 WP_174181830.1 C4-type zinc ribbon domain-containing protein
208(1) WP_205654073.1 WP_205654073.1 C4-type zinc ribbon domain-containing protein

209(1) WP_149378849.1 WP_149378849.1 alpha/beta fold hydrolase
209(1) WP_225507736.1 WP_225507736.1 alpha/beta fold hydrolase

210(1) WP_229693944.1 WP_229693944.1 MFS transporter
210(1) WP_006554384.1 WP_006554384.1 MULTISPECIES: OFA family MFS transporter

211(1) WP_231997694.1 WP_231997694.1 MULTISPECIES: hypothetical protein
211(1) WP_067760604.1 WP_067760604.1 MULTISPECIES: hypothetical protein

212(1) WP_105569093.1 WP_105569093.1 MULTISPECIES: VanW family protein
212(1) WP_236641073.1 WP_236641073.1 VanW family protein

213(1) WP_238935399.1 WP_238935399.1 iron-siderophore ABC transporter substrate-binding protein
213(1) WP_231839557.1 WP_231839557.1 iron-siderophore ABC transporter substrate-binding protein

214(1) WP_185277594.1 WP_185277594.1 SGNH/GDSL hydrolase

family protein
214(1) WP_241656206.1 WP_241656206.1 SGNH/GDSL hydrolase family protein

215(1) WP_205654062.1 WP_205654062.1 Gfo/Idh/MocA family oxidoreductase
215(1) WP_251423485.1 WP_251423485.1 Gfo/Idh/MocA family oxidoreductase

216(1) WP_251746081.1 WP_251746081.1 hypothetical protein, partial
216(1) WP_045877963.1 WP_045877963.1 hypothetical protein

217(1) WP_251746083.1 WP_251746083.1 pirin family protein
217(1) WP_045877962.1 WP_045877962.1 pirin family protein

218(1) WP_147916384.1 WP_147916384.1 ABC transporter permease
218(1) WP_254584109.1 WP_254584109.1 ABC transporter permease

219(1) WP_254584115.1 WP_254584115.1 thioesterase family protein
219(1) WP_010596343.1 WP_010596343.1 MULTISPECIES: thioesterase family protein

220(1) WP_187437798.1 WP_187437798.1 hypothetical protein
220(1) WP_255268926.1 WP_255268926.1 hypothetical protein

221(1) WP_154347595.1 WP_154347595.1 hypothetical protein
221(1) WP_258046105.1 WP_258046105.1 hypothetical protein

222(1) WP_128470350.1 WP_128470350.1 MULTISPECIES: VOC family protein

222(1) WP_258133864.1 WP_258133864.1 MULTISPECIES: glyoxalase

223(1) WP_259307155.1 WP_259307155.1 aminopeptidase N
223(1) WP_134353687.1 WP_134353687.1 MULTISPECIES: aminopeptidase N

224(1) WP_259809478.1 WP_259809478.1 cation transporter
224(1) WP_141368720.1 WP_141368720.1 MULTISPECIES: heavy-metal-associated domain-containing protein

225(1) WP_056153680.1 WP_056153680.1 MULTISPECIES: helix-turn-helix domain-containing GNAT family N-acetyltransferase
225(1) WP_262347580.1 WP_262347580.1 helix-turn-helix domain-containing GNAT family N-acetyltransferase

226(1) WP_147916368.1 WP_147916368.1 TetR/AcrR family transcriptional regulator
226(1) WP_263021836.1 WP_263021836.1 TetR/AcrR family transcriptional regulator

227(1) WP_263021848.1 WP_263021848.1 hypothetical protein
227(1) WP_091063604.1 WP_091063604.1 hypothetical protein

228(1) WP_246607921.1 WP_246607921.1 fructosamine kinase family protein
228(1) WP_263021857.1 WP_263021857.1 fructosamine kinase family protein

229(1) WP_213011474.1 WP_213011474.1 hypothetical protein
229(1) WP_263021863.1 WP_263021863.1 hypothetical protein

230(1) WP_266514985.1 WP_266514985.1 MULTISPECIES:

hypothetical protein
230(1) WP_006131570.1 WP_006131570.1 MULTISPECIES: hypothetical protein

231(2) WP_266759084.1 WP_266759084.1 MULTISPECIES: helix-turn-helix transcriptional regulator

232(2) WP_266759086.1 WP_266759086.1 MULTISPECIES: DUF397 domain-containing protein

233(1) WP_278175642.1 WP_278175642.1 glycosyltransferase family 87 protein
233(1) WP_091063609.1 WP_091063609.1 glycosyltransferase 87 family protein

234(1) WP_091063601.1 WP_091063601.1 YcnI family protein
234(1) WP_278175643.1 WP_278175643.1 YcnI family protein

235(1) WP_213011489.1 WP_213011489.1 GNAT family N-acetyltransferase
235(1) WP_278181960.1 WP_278181960.1 GNAT family N-acetyltransferase

236(1) WP_283320821.1 WP_283320821.1 hypothetical protein
236(1) WP_147796145.1 WP_147796145.1 hypothetical protein

237(1) WP_283320812.1 WP_283320812.1 DUF2142 domain-containing protein
237(1) WP_283320823.1 WP_283320823.1 glycosyltransferase family 39 protein

238(1) WP_147796139.1 WP_147796139.1 hypothetical protein
238(1) WP_283320829.1 WP_283320829.1 hypothetical protein

239(1) WP_229693943.1 WP_229693943.1 PH domain-containing protein
239(1) WP_283663411.1 WP_283663411.1 PH domain-containing protein

240(1) WP_189158534.1 WP_189158534.1 PH domain-containing protein
240(1) WP_283663412.1 WP_283663412.1 PH domain-containing protein

241(1) WP_210648823.1 WP_210648823.1 thiamine pyrophosphate-requiring protein
241(1) WP_283663421.1 WP_283663421.1 thiamine pyrophosphate-binding protein

242(1) WP_056917809.1 WP_056917809.1 alpha/beta hydrolase
242(1) WP_283666104.1 WP_283666104.1 alpha/beta hydrolase

243(1) WP_308738544.1 WP_308738544.1 ATP-dependent helicase
243(1) WP_259813868.1 WP_259813868.1 ATP-dependent helicase

244(1) WP_053918101.1 WP_053918101.1 MULTISPECIES: hypothetical protein
244(1) WP_308738554.1 WP_308738554.1 hypothetical protein

245(1) WP_133677922.1 WP_133677922.1 MULTISPECIES: acyl-CoA thioesterase
245(1) WP_308738556.1 WP_308738556.1 acyl-CoA thioesterase

246(1) WP_308738559.1 WP_308738559.1 GTP pyrophosphokinase family protein
246(1) WP_259813887.1 WP_259813887.1 GTP pyrophosphokinase family protein

247(1) WP_308738563.1 WP_308738563.1 Pr6Pr family membrane protein
247(1) WP_259813894.1 WP_259813894.1 Pr6Pr family membrane protein

248(1) WP_310112498.1 WP_310112498.1 HNH endonuclease family protein
248(1) WP_205654068.1 WP_205654068.1 HNH endonuclease family protein

249(1) WP_205654064.1 WP_205654064.1 DNA alkylation repair protein
249(1) WP_310112502.1 WP_310112502.1 DNA alkylation repair protein

250(1) WP_056153683.1 WP_056153683.1 MULTISPECIES: prolyl oligopeptidase family serine peptidase
250(1) WP_310112504.1 WP_310112504.1 alpha/beta hydrolase

251(1) WP_310112506.1 WP_310112506.1 dihydrodipicolinate synthase family protein
251(1) WP_205654061.1 WP_205654061.1 dihydrodipicolinate synthase family protein

252(1) WP_310112507.1 WP_310112507.1 sugar phosphate isomerase/epimerase family protein
252(1) WP_240460376.1 WP_240460376.1 sugar phosphate isomerase/epimerase

253(1) WP_310112510.1 WP_310112510.1 ROK family protein
253(1) WP_156742049.1 WP_156742049.1 ROK family transcriptional regulator

254(1) WP_315912378.1 WP_315912378.1 hypothetical protein

254(1) WP_014375109.1 WP_014375109.1 hypothetical protein

255(1) WP_318603915.1 WP_318603915.1 hypothetical protein
255(1) WP_259813883.1 WP_259813883.1 glycine cleavage T C-terminal barrel domain-containing protein

256(1) WP_320965256.1 WP_320965256.1 hypothetical protein
256(1) WP_128470348.1 WP_128470348.1 hypothetical protein

257(1) WP_128470339.1 WP_128470339.1 hypothetical protein
257(1) WP_320965266.1 WP_320965266.1 hypothetical protein

258(1) WP_324613571.1 WP_324613571.1 ECF transporter S component
258(1) WP_185277605.1 WP_185277605.1 ECF transporter S component

259(1) WP_185277591.1 WP_185277591.1 cation:proton antiporter
259(1) WP_324613596.1 WP_324613596.1 cation:proton antiporter

260(1) WP_326338215.1 WP_326338215.1 class I SAM-dependent DNA methyltransferase
260(1) WP_283663404.1 WP_283663404.1 class I SAM-dependent DNA methyltransferase

261(1) WP_283663406.1 WP_283663406.1 DEAD/DEAH box helicase family protein
261(1) WP_326338217.1 WP_326338217.1 type I restriction endonuclease subunit R

262(1) WP_150242282.1 WP_150242282.1 bifunctional 3'-5' exonuclease/DNA polymerase

262(1) WP_326338225.1 WP_326338225.1 bifunctional 3'-5' exonuclease/DNA polymerase

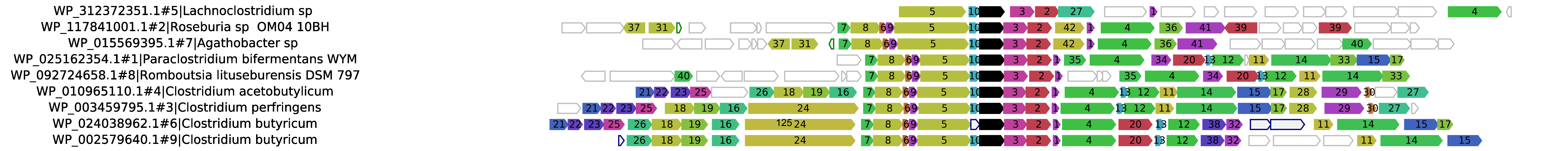

WP_312372351.1#5|Lachnoclostridium sp
WP_117841001.1#2|Roseburia sp OM04 10BH
WP_015569395.1#7|Agathobacter sp
WP_025162354.1#1|Paraclostridium bifermentans WYM
WP_092724658.1#8|Romboutsia lituseburensis DSM 797
WP_010965110.1#4|Clostridium acetobutylicum
WP_003459795.1#3|Clostridium perfringens
WP_024038962.1#6|Clostridium butyricum
WP_002579640.1#9|Clostridium butyricum

1(1)   WP_022242572.1 WP_022242572.1 MULTISPECIES: 30S ribosomal protein S15
1(2)   WP_002579643.1 WP_002579643.1 MULTISPECIES: 30S ribosomal protein S15
1(1)   WP_003449423.1 WP_003449423.1 MULTISPECIES: 30S ribosomal protein S15
1(1)   WP_170827933.1 WP_170827933.1 30S ribosomal protein S15
1(1)   WP_010965113.1 WP_010965113.1 MULTISPECIES: 30S ribosomal protein S15
1(1)   WP_312103426.1 WP_312103426.1 30S ribosomal protein S15
1(1)   WP_012742776.1 WP_012742776.1 MULTISPECIES: 30S ribosomal protein S15
1(1)   WP_021428907.1 WP_021428907.1 MULTISPECIES: 30S ribosomal protein S15

2(1)   WP_010965112.1 WP_010965112.1 MULTISPECIES: bifunctional riboflavin kinase/FAD synthetase
2(2)   WP_002579642.1 WP_002579642.1 MULTISPECIES: bifunctional riboflavin kinase/FAD synthetase
2(1)   WP_022242574.1 WP_022242574.1 MULTISPECIES: bifunctional riboflavin kinase/FAD synthetase
2(1)   WP_015569393.1 WP_015569393.1 MULTISPECIES: bifunctional riboflavin kinase/FAD synthetase
2(1)   WP_312103434.1 WP_312103434.1 bifunctional riboflavin kinase/FAD synthetase
2(1)   WP_092724656.1 WP_092724656.1 bifunctional riboflavin kinase/FAD synthetase
2(1)   WP_021428771.1 WP_021428771.1 bifunctional riboflavin kinase/FAD synthetase
2(1)   WP_003459763.1 WP_003459763.1 bifunctional riboflavin kinase/FAD synthetase

3(1)   WP_021428766.1 WP_021428766.1 tRNA pseudouridine(55) synthase TruB
3(1)   WP_002579641.1 WP_002579641.1 MULTISPECIES: tRNA pseudouridine(55) synthase TruB
3(1)   WP_010965111.1 WP_010965111.1 MULTISPECIES: tRNA pseudouridine(55) synthase TruB

3(1)   WP_312103436.1 WP_312103436.1 tRNA pseudouridine(55) synthase TruB
3(1)   WP_092724657.1 WP_092724657.1 tRNA pseudouridine(55) synthase TruB
3(1)   WP_015569394.1 WP_015569394.1 MULTISPECIES: tRNA pseudouridine(55) synthase TruB
3(1)   WP_022242575.1 WP_022242575.1 MULTISPECIES: tRNA pseudouridine(55) synthase TruB
3(1)   WP_024038963.1 WP_024038963.1 MULTISPECIES: tRNA pseudouridine(55) synthase TruB
3(1)   WP_003459755.1 WP_003459755.1 tRNA pseudouridine(55) synthase TruB

4(1)   WP_041254072.1 WP_041254072.1 MULTISPECIES: polyribonucleotide nucleotidyltransferase
4(1)   WP_312372342.1 WP_312372342.1 polyribonucleotide nucleotidyltransferase
4(1)   WP_010965114.1 WP_010965114.1 MULTISPECIES: polyribonucleotide nucleotidyltransferase
4(1)   WP_025162353.1 WP_025162353.1 polyribonucleotide nucleotidyltransferase
4(1)   WP_024038965.1 WP_024038965.1 polyribonucleotide nucleotidyltransferase
4(1)   WP_002579644.1 WP_002579644.1 MULTISPECIES: polyribonucleotide nucleotidyltransferase
4(1)   WP_117697175.1 WP_117697175.1 MULTISPECIES: polyribonucleotide nucleotidyltransferase
4(1)   WP_092724651.1 WP_092724651.1 polyribonucleotide nucleotidyltransferase
4(1)   WP_003459742.1 WP_003459742.1 polyribonucleotide nucleotidyltransferase

5(1)   WP_308801322.1 WP_308801322.1 translation initiation factor IF-2
5(2)   WP_002579637.1 WP_002579637.1 MULTISPECIES: translation initiation factor IF-2
5(1)   WP_010965108.1 WP_010965108.1 MULTISPECIES: translation initiation factor IF-2
5(1)   WP_117946900.1 WP_117946900.1 MULTISPECIES: translation initiation factor IF-2

5(1)   WP_312372353.1 WP_312372353.1 translation initiation factor IF-2, partial
5(1)   WP_003449445.1 WP_003449445.1 MULTISPECIES: translation initiation factor IF-2
5(1)   WP_092724660.1 WP_092724660.1 translation initiation factor IF-2
5(1)   WP_021428872.1 WP_021428872.1 translation initiation factor IF-2

6(2)   WP_002579635.1 WP_002579635.1 MULTISPECIES: YlxR family protein
6(1)   WP_012742785.1 WP_012742785.1 MULTISPECIES: YlxR family protein
6(1)   WP_022242580.1 WP_022242580.1 MULTISPECIES: YlxR family protein
6(1)   WP_003459790.1 WP_003459790.1 MULTISPECIES: YlxR family protein
6(1)   WP_092724662.1 WP_092724662.1 YlxR family protein
6(1)   WP_010965106.1 WP_010965106.1 MULTISPECIES: YlxR family protein
6(1)   WP_021428756.1 WP_021428756.1 MULTISPECIES: YlxR family protein

7(1)   WP_022293380.1 WP_022293380.1 MULTISPECIES: ribosome maturation factor RimP
7(1)   WP_022242582.1 WP_022242582.1 MULTISPECIES: ribosome maturation factor RimP
7(1)   WP_092724664.1 WP_092724664.1 ribosome maturation factor RimP
7(1)   WP_021428857.1 WP_021428857.1 MULTISPECIES: ribosome maturation factor RimP
7(1)   WP_003459796.1 WP_003459796.1 MULTISPECIES: ribosome maturation factor RimP
7(2)   WP_002579633.1 WP_002579633.1 MULTISPECIES: ribosome maturation factor RimP
7(1)   WP_010965104.1 WP_010965104.1 MULTISPECIES: ribosome maturation factor RimP

8(1)   WP_021428826.1 WP_021428826.1 MULTISPECIES:

transcription termination factor NusA
8(1)   WP_003449452.1 WP_003449452.1 MULTISPECIES: transcription termination factor NusA
8(1)   WP_055222720.1 WP_055222720.1 MULTISPECIES: transcription termination factor NusA
8(1)   WP_092724663.1 WP_092724663.1 transcription termination factor NusA
8(1)   WP_010965105.1 WP_010965105.1 MULTISPECIES: transcription termination factor NusA
8(1)   WP_117946899.1 WP_117946899.1 transcription termination factor NusA
8(2)   WP_002579634.1 WP_002579634.1 MULTISPECIES: transcription termination factor NusA

9(1)   WP_124229448.1 WP_124229448.1 ribosomal L7Ae/L30e/S12e/Gadd45 family protein
9(1)   WP_010965107.1 WP_010965107.1 MULTISPECIES: 50S ribosomal protein L7ae-like protein
9(1)   WP_025162355.1 WP_025162355.1 MULTISPECIES: ribosomal L7Ae/L30e/S12e/Gadd45 family protein
9(1)   WP_022242579.1 WP_022242579.1 MULTISPECIES: ribosomal L7Ae/L30e/S12e/Gadd45 family protein
9(1)   WP_003459801.1 WP_003459801.1 50S ribosomal protein L7ae-like protein
9(1)   WP_092724661.1 WP_092724661.1 ribosomal L7Ae/L30e/S12e/Gadd45 family protein
9(1)   WP_022293381.1 WP_022293381.1 ribosomal L7Ae/L30e/S12e/Gadd45 family protein
9(1)   WP_002579636.1 WP_002579636.1 MULTISPECIES: ribosomal L7Ae/L30e/S12e/Gadd45 family protein

10(1)   WP_092724659.1 WP_092724659.1 30S ribosome-binding factor RbfA
10(1)   WP_022242577.1 WP_022242577.1 MULTISPECIES: 30S ribosome-binding factor RbfA
10(1)   WP_312103442.1 WP_312103442.1 30S ribosome-binding factor RbfA
10(1)   WP_002579639.1 WP_002579639.1 MULTISPECIES: 30S ribosome-binding factor RbfA
10(1)   WP_003449473.1 WP_003449473.1 MULTISPECIES: 30S

ribosome-binding factor RbfA
10(1) WP_021428779.1 WP_021428779.1 MULTISPECIES: 30S ribosome-binding factor RbfA
10(1) WP_010965109.1 WP_010965109.1 MULTISPECIES: 30S ribosome-binding factor RbfA
10(1) WP_012742782.1 WP_012742782.1 MULTISPECIES: 30S ribosome-binding factor RbfA

11(1) WP_092724641.1 WP_092724641.1 ATP-dependent Clp protease proteolytic subunit
11(1) WP_077175946.1 WP_077175946.1 ATP-dependent Clp protease proteolytic subunit
11(1) WP_057231703.1 WP_057231703.1 ATP-dependent Clp protease proteolytic subunit
11(1) WP_003428384.1 WP_003428384.1 ATP-dependent Clp protease proteolytic subunit
11(1) WP_002579653.1 WP_002579653.1 MULTISPECIES: ATP-dependent Clp protease proteolytic subunit
11(1) WP_010965117.1 WP_010965117.1 MULTISPECIES: ClpP family protease

12(1) WP_092724643.1 WP_092724643.1 aspartate kinase
12(1) WP_003428394.1 WP_003428394.1 aspartate kinase
12(1) WP_010965116.1 WP_010965116.1 MULTISPECIES: aspartate kinase
12(1) WP_003459758.1 WP_003459758.1 MULTISPECIES: aspartate kinase
12(1) WP_035762424.1 WP_035762424.1 aspartate kinase
12(1) WP_021428850.1 WP_021428850.1 MULTISPECIES: aspartate kinase

13(1) WP_003459765.1 WP_003459765.1 MULTISPECIES: YlmC/YmxH family sporulation protein
13(1) WP_092724645.1 WP_092724645.1 YlmC/YmxH family sporulation protein
13(1) WP_021428747.1 WP_021428747.1 MULTISPECIES: YlmC/YmxH family sporulation protein
13(1) WP_010965115.1 WP_010965115.1 MULTISPECIES: YlmC/YmxH family sporulation protein

13(2)   WP_002579646.1 WP_002579646.1 MULTISPECIES: YlmC/YmxH family sporulation protein

14(1)   WP_010965118.1 WP_010965118.1 MULTISPECIES: DNA translocase FtsK
14(1)   WP_146867778.1 WP_146867778.1 DNA translocase FtsK
14(1)   WP_061415777.1 WP_061415777.1 MULTISPECIES: DNA translocase FtsK
14(1)   WP_035762410.1 WP_035762410.1 DNA translocase FtsK
14(1)   WP_092724639.1 WP_092724639.1 DNA translocase FtsK
14(1)   WP_025162351.1 WP_025162351.1 MULTISPECIES: DNA translocase FtsK

15(1)   WP_003459746.1 WP_003459746.1 MULTISPECIES: 30S ribosomal protein S12 methylthiotransferase RimO
15(1)   WP_025162350.1 WP_025162350.1 MULTISPECIES: 30S ribosomal protein S12 methylthiotransferase RimO
15(1)   WP_171781613.1 WP_171781613.1 30S ribosomal protein S12 methylthiotransferase RimO
15(1)   WP_010965119.1 WP_010965119.1 MULTISPECIES: 30S ribosomal protein S12 methylthiotransferase RimO
15(1)   WP_169512802.1 WP_169512802.1 MULTISPECIES: 30S ribosomal protein S12 methylthiotransferase RimO

16(1)   WP_010965103.1 WP_010965103.1 MULTISPECIES: flavodoxin-dependent (E)-4-hydroxy-3-methylbut-2-enyl-diphosphate synthase
16(1)   WP_003469134.1 WP_003469134.1 flavodoxin-dependent (E)-4-hydroxy-3-methylbut-2-enyl-diphosphate synthase
16(2)   WP_002579631.1 WP_002579631.1 MULTISPECIES: flavodoxin-dependent (E)-4-hydroxy-3-methylbut-2-enyl-diphosphate synthase

17(1)   WP_003428378.1 WP_003428378.1 MULTISPECIES: CDP-diacylglycerol--glycerol-3-phosphate 3-phosphatidyltransferase
17(1)   WP_003469140.1 WP_003469140.1 MULTISPECIES: CDP-diacylglycerol--glycerol-3-phosphate

3-phosphatidyltransferase
17(1) WP_010965120.1 WP_010965120.1 MULTISPECIES: CDP-diacylglycerol--glycerol-3-phosphate 3-phosphatidyltransferase
17(1) WP_021428714.1 WP_021428714.1 MULTISPECIES: CDP-diacylglycerol--glycerol-3-phosphate 3-phosphatidyltransferase

18(1) WP_002579629.1 WP_002579629.1 MULTISPECIES: 1-deoxy-D-xylulose-5-phosphate reductoisomerase
18(1) WP_003459741.1 WP_003459741.1 1-deoxy-D-xylulose-5-phosphate reductoisomerase
18(1) WP_010965101.1 WP_010965101.1 MULTISPECIES: 1-deoxy-D-xylulose-5-phosphate reductoisomerase
18(1) WP_024038959.1 WP_024038959.1 1-deoxy-D-xylulose-5-phosphate reductoisomerase

19(1) WP_002579630.1 WP_002579630.1 MULTISPECIES: RIP metalloprotease RseP
19(1) WP_010965102.1 WP_010965102.1 MULTISPECIES: RIP metalloprotease RseP
19(1) WP_024038960.1 WP_024038960.1 RIP metalloprotease RseP
19(1) WP_003466788.1 WP_003466788.1 MULTISPECIES: RIP metalloprotease RseP

20(1) WP_021428870.1 WP_021428870.1 pitrilysin family protein
20(1) WP_092724647.1 WP_092724647.1 pitrilysin family protein
20(1) WP_035762428.1 WP_035762428.1 MULTISPECIES: pitrilysin family protein
20(1) WP_003428399.1 WP_003428399.1 MULTISPECIES: pitrilysin family protein

21(1) WP_003415502.1 WP_003415502.1 MULTISPECIES: UMP kinase
21(1) WP_010965095.1 WP_010965095.1 MULTISPECIES: UMP

kinase
21(1)   WP_003459788.1 WP_003459788.1 MULTISPECIES: UMP kinase

22(1)   WP_010965096.1 WP_010965096.1 MULTISPECIES: ribosome recycling factor
22(1)   WP_003459781.1 WP_003459781.1 MULTISPECIES: ribosome recycling factor
22(1)   WP_003407900.1 WP_003407900.1 MULTISPECIES: ribosome recycling factor

23(1)   WP_003407899.1 WP_003407899.1 MULTISPECIES: isoprenyl transferase
23(1)   WP_003459793.1 WP_003459793.1 isoprenyl transferase
23(1)   WP_010965097.1 WP_010965097.1 MULTISPECIES: isoprenyl transferase

24(1)   WP_035762433.1 WP_035762433.1 MULTISPECIES: PolC-type DNA polymerase III
24(1)   WP_061415779.1 WP_061415779.1 PolC-type DNA polymerase III
24(1)   WP_146867779.1 WP_146867779.1 PolC-type DNA polymerase III

25(1)   WP_146867781.1 WP_146867781.1 phosphatidate cytidylyltransferase
25(1)   WP_013913673.1 WP_013913673.1 MULTISPECIES: phosphatidate cytidylyltransferase
25(1)   WP_003459799.1 WP_003459799.1 MULTISPECIES: phosphatidate cytidylyltransferase

26(1)   WP_207714534.1 WP_207714534.1 AI-2E family transporter
26(1)   WP_035762435.1 WP_035762435.1 MULTISPECIES: AI-2E family transporter
26(1)   WP_010965100.1 WP_010965100.1 MULTISPECIES: AI-2E family transporter

27(1)   WP_013913643.1 WP_013913643.1 MULTISPECIES: pyridoxal phosphate-dependent aminotransferase
27(1)   WP_312103431.1 WP_312103431.1 PLP-dependent aminotransferase family protein
27(1)   WP_061415775.1 WP_061415775.1 pyridoxal phosphate-dependent aminotransferase

28(1)   WP_003459804.1 WP_003459804.1 MULTISPECIES: recombinase RecA
28(1)   WP_010965121.1 WP_010965121.1 MULTISPECIES: recombinase RecA

29(1)   WP_003459753.1 WP_003459753.1 MULTISPECIES: ribonuclease Y
29(1)   WP_010965122.1 WP_010965122.1 MULTISPECIES: ribonuclease Y

30(1)   WP_003459803.1 WP_003459803.1 MULTISPECIES: stage V sporulation protein S
30(1)   WP_010965123.1 WP_010965123.1 MULTISPECIES: stage V sporulation protein S

31(1)   WP_022242586.1 WP_022242586.1 MULTISPECIES: UDP-glucose 4-epimerase GalE
31(1)   WP_015516548.1 WP_015516548.1 MULTISPECIES: UDP-glucose 4-epimerase GalE

32(1)   WP_035762419.1 WP_035762419.1 sugar O-acetyltransferase
32(1)   WP_003407907.1 WP_003407907.1 MULTISPECIES: sugar O-acetyltransferase

33(1)   WP_021428709.1 WP_021428709.1 tRNA 2-selenouridine(34) synthase MnmH

33(1)   WP_092724637.1 WP_092724637.1 tRNA 2-selenouridine(34) synthase MnmH

34(1)   WP_092724649.1 WP_092724649.1 polysaccharide deacetylase family protein
34(1)   WP_021428800.1 WP_021428800.1 MULTISPECIES: polysaccharide deacetylase family protein

35(1)   WP_021428904.1 WP_021428904.1 DegV family protein
35(1)   WP_092724653.1 WP_092724653.1 DegV family protein

36(1)   WP_117697177.1 WP_117697177.1 MULTISPECIES: S8 family peptidase
36(1)   WP_022293387.1 WP_022293387.1 MULTISPECIES: S8 family peptidase

37(1)   WP_117840997.1 WP_117840997.1 MULTISPECIES: helix-turn-helix domain-containing protein
37(1)   WP_012742789.1 WP_012742789.1 MULTISPECIES: AraC family transcriptional regulator

38(1)   WP_124229446.1 WP_124229446.1 DMT family transporter
38(1)   WP_002579648.1 WP_002579648.1 MULTISPECIES: DMT family transporter

39(1)   WP_117946903.1 WP_117946903.1 MULTISPECIES: D-alanyl-D-alanine carboxypeptidase family protein
39(1)   WP_243006639.1 WP_243006639.1 MULTISPECIES: D-alanyl-D-alanine carboxypeptidase family protein

40(1)   WP_308801314.1 WP_308801314.1 ABC transporter ATP-binding protein
40(1)   WP_092724671.1 WP_092724671.1 ABC transporter ATP-binding protein

41(1)   WP_117946902.1 WP_117946902.1 MULTISPECIES: cardiolipin synthase
41(1)   WP_308801320.1 WP_308801320.1 cardiolipin synthase

42(1)   WP_308801321.1 WP_308801321.1 SH3 domain-containing C40 family peptidase
42(1)   WP_117946901.1 WP_117946901.1 MULTISPECIES: NlpC/P60 family protein
