# Supplementary material for: Structural and bioinformatics analyses identify deoxydinucleotide-specific nucleases and their association with genomic islands in gram-positive bacteria
Source: Nucleic Acids Res. 2025 Jan 8;53(1):gkae1235. doi: 10.1093/nar/gkae1235 (PMC11706625; doi:10.1093/nar/gkae1235)
Supplement: gkae1235_Supplemental_Files [file gkae1235_supplemental_files.zip › diDNase_supplement_FiguresTables_NAR_R1_fixed2.pdf]

Supplementary Data

Structural and bioinformatics analyses identify deoxydinucleotide-specific nucleases and their association with genomic islands in Gram-positive bacteria

Sofia Mortensen[1], Stanislava Kuncová[1], Justin D. Lormand[1], Tanner M. Myers[3], Soo-Kyoung Kim[4], Vincent T. Lee[4], Wade C. Winkler[3,4], and Holger Sondermann[1,2,*]

CONTENT

Figure S1. *Sequence alignment and conservation between NrnC homologs in Gram-negative and Gram-positive bacteria.*

Figure S2. *Enzymatic activity of NrnC homologs from Gram-positive and NrnC orthologs from Gram-negative bacteria.*

Figure S3. *Enzymatic activity of diDNase on single- and double-stranded DNA oligonucleotides.*

Figure S4. *Structural basis of diDNase activity.*

Figure S5. *Characterization of Actinomycetal diDNases in vivo and comparison of Actinomycetal and Gammaproteobacterial Orn.*

Table S1. *Representative NrnC homolog sequences from different bacterial orders.*

Table S2. *X-ray diffraction data processing and refinement statistics.*

Table S3. *Enzyme kinetics parameters of NrnC homologs.*

Table S4. *Enzyme kinetic parameters of diDNase$_{Noc}$ towards diverse substrates.*

Table S5. *Enzyme kinetic parameters of mutants of diDNase$_{Noc}$ and NrnC$_{Bh}$.*

Table S6. *Enzyme kinetics parameters of Orn homologs.*

Table S7. *Details of the genomic environment analysis.*

SUPPLEMENTARY FIGURES

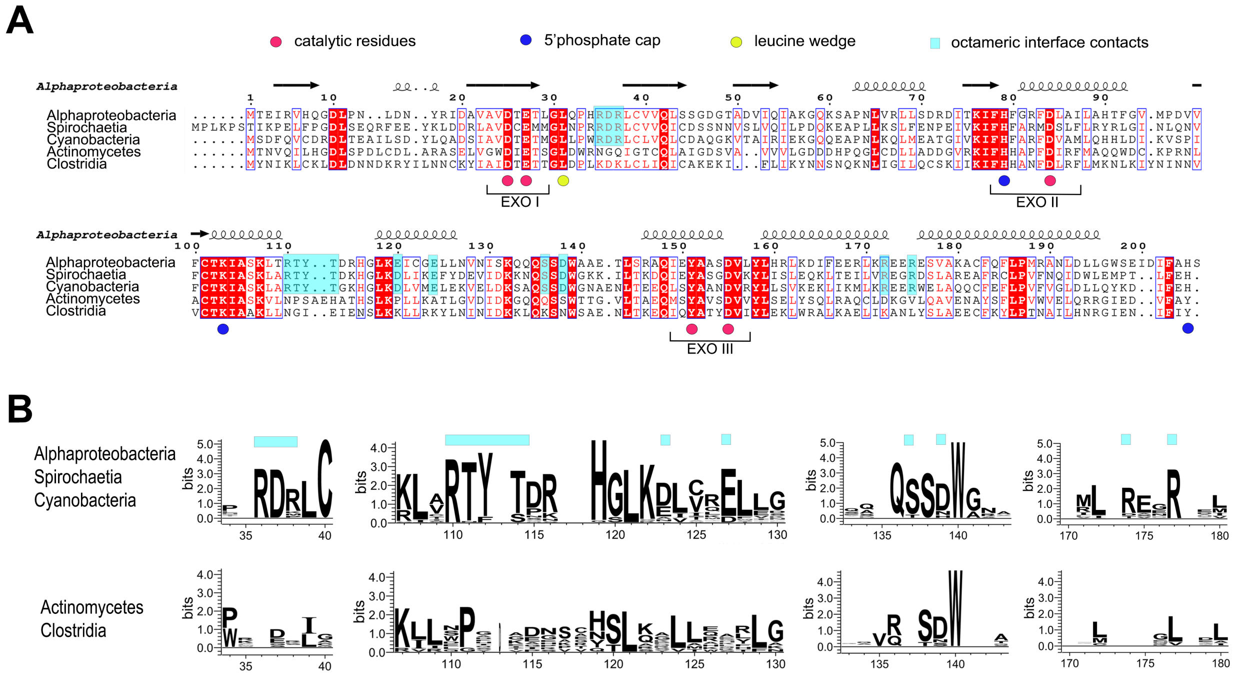

Figure S1. Sequence alignment and conservation between NrnC homologs in Gram-negative and Gram-positive bacteria. **A.** Sequence alignment of representative NrnC homologs from five classes. *Bartonella* from Alphaproteobacteria, *Leptospira* from Spirochaetia, *Okeania* from Cyanobacteria, *Clostridium* from Clostridia and *Rhodococcus* from Actinomycetes. **B.** Sequence logos showing conservation of residues involved in octameric assembly of NrnCs in Gram-negative but not in Gram-positive bacteria. Logos were created based on multiple sequence alignments used to construct the tree in Figure 1A. Amino acid numbering, secondary structure mapping, and assignment of functions for catalysis, 5' phosphate coordination, substrate wedging, and octamerization are based on *B. henselae* NrnC and its structure (PBD ID 7MPL).

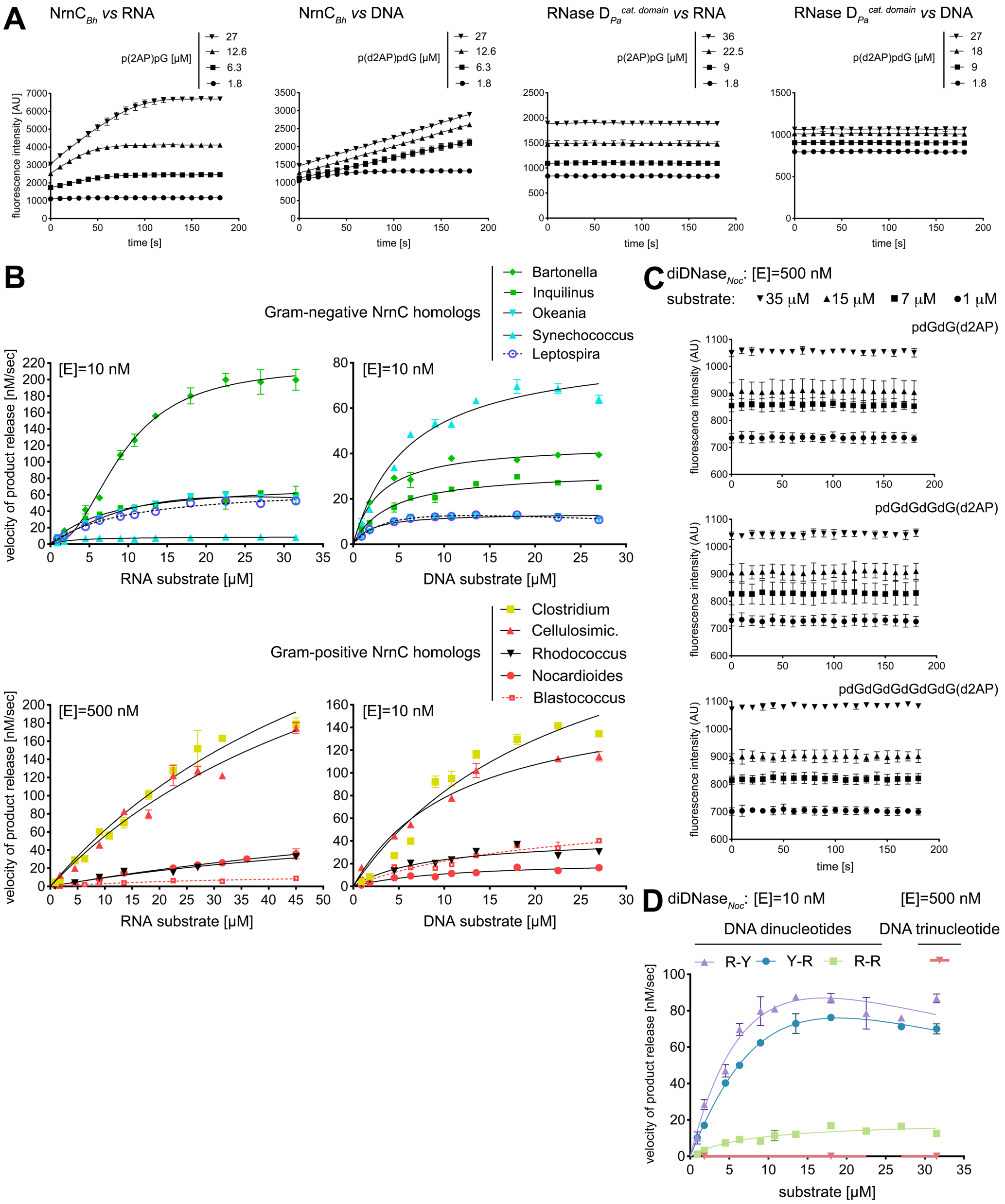

Figure S2. Enzymatic activity of NrnC homologs from Gram-positive and NrnC homologs from Gram-negative bacteria. **A.** Enzymatic activity of NrnC$_{Bh}$ and the catalytic domain of RNase D$_{Pa}$. Fluorescence intensity changes over time (excitation at 310 nm/ emission at 375 nm) are plotted. Mean value of three measurements and SDs are shown. **B.** Enzymatic activity of NrnC homologs towards RNA (p(2AP)G) and DNA (p(d2AP)dG) substrates. Data were fitted with a Michaelis-Menten model, except for *Bartonella* NrnC with RNA substrate, which were fitted an allosteric sigmoidal model. The enzyme concentrations used in different measurements are indicated. **C**. Enzymatic activity of diDNase$_{Noc}$ on longer nano-DNAs. Time courses of the fluorescence intensity changes over time when longer ssDNAs with 3, 5, or 7 nucleotides were used as substrates for diDNase$_{Noc}$. Graphs show mean values from triplicate experiments. Error bars represent SDs. **D.** Enzymatic activity of diDNase$_{Noc}$ on deoxynucleotide substrates with combinations of purine-purine (R-R), purine-pyrimidine (R-Y), or pyrimidine-purine (Y-R) nucleobases. The data for the R-Y and Y-R substrates were fitted using a substrate-inhibition model.

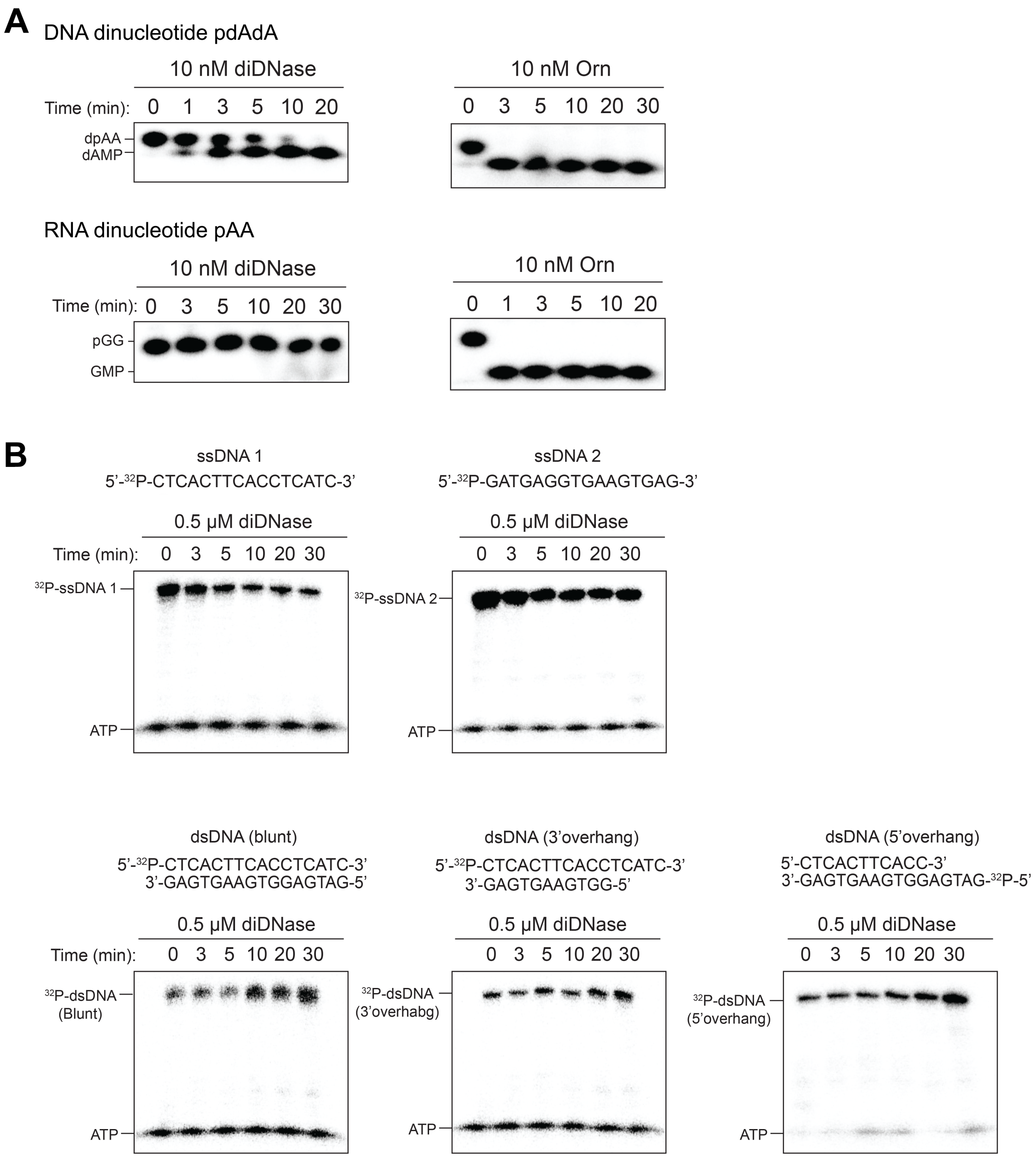

Figure S3. Enzymatic activity of diDNase on single- and double-stranded DNA oligonucleotides. **A.** Enzymatic activity of diDNase$_{Noc}$ and Orn$_{Vc}$ on 5'-^{32}P-labeled dinucleotides. **B.** Enzymatic activity of diDNase$_{Noc}$ on 5'-^{32}P-labeled ssDNA and (blunt-end, 3' overhang, 5' overhang) dsDNA oligonucleotides. Reactions were stopped at the indicated time intervals. Representative denaturing 20% Urea PAGE resolving reaction mixtures are shown.

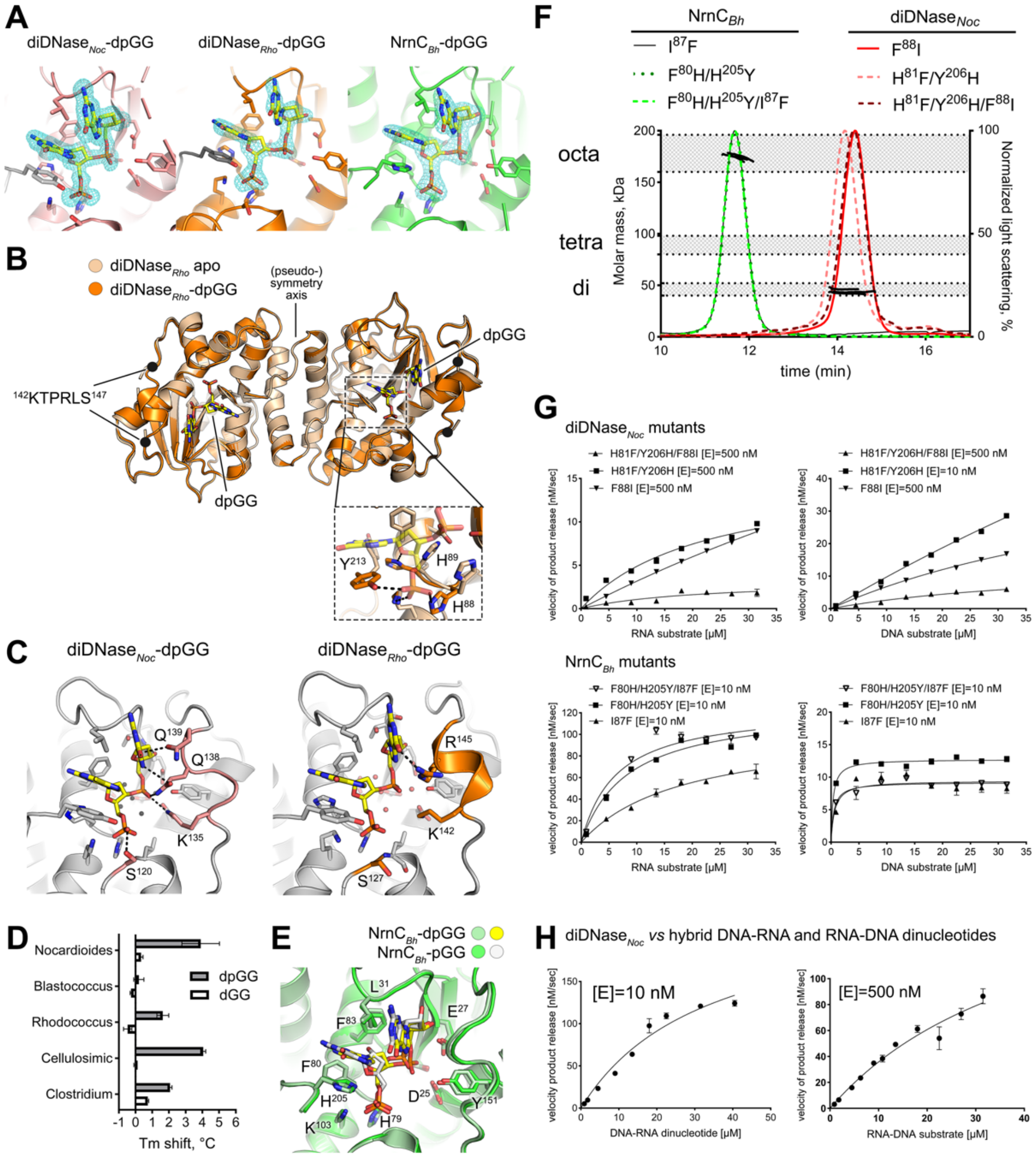

Figure S4. Structural basis of diDNase activity. **A.** Electron density polder maps for the substrate dpGG in the structures of complexes with diDNase$_{Rho}$, diDNase$_{Noc}$, and NrnC$_{Bh}$. **B.** Structural superposition of diDNase$_{Rho}$ with and without dpGG. **C.** Comparison of the active sites of diDNase$_{Rho}$ and diDNase$_{Noc}$ bound to dpGG. **D.** DiDNase melting temperature (T_m) in the presence of deoxydinucleotides with and without 5' phosphate. Each measurement was performed in triplicate and the mean values with SDs were plotted. **E.** Comparison of the active site of NrnC$_{Bh}$ bound to pGG (PDB ID 7MPL) and dpGG. **F**. Oligomeric state of NrnC$_{Bh}$ and diDNase$_{Noc}$ mutants determined by SEC-MALS. The normalized light scattering signal (color-coded lines, right axis) and molar mass values (black dots, left axis) were plotted against the SEC elution volume. Grey shaded areas show the theoretical molar mass ranges for dimeric, tetrameric, and octameric assemblies based on the primary sequence of the proteins. **G**. Enzymatic activity of mutants of diDNase$_{Noc}$ and NrnC$_{Bh}$ towards RNA and DNA dinucleotides. Data were fitted with a Michaelis-Menten model. The enzyme concentrations used in different measurements are indicated. **H.** Enzymatic activity of diDNase$_{Noc}$ towards hybrid DNA-RNA and RNA-DNA dinucleotides, fitted using a Michaelis-Menten model. Graphs in panels G and H show mean values from triplicate experiments and error bars indicate SDs.

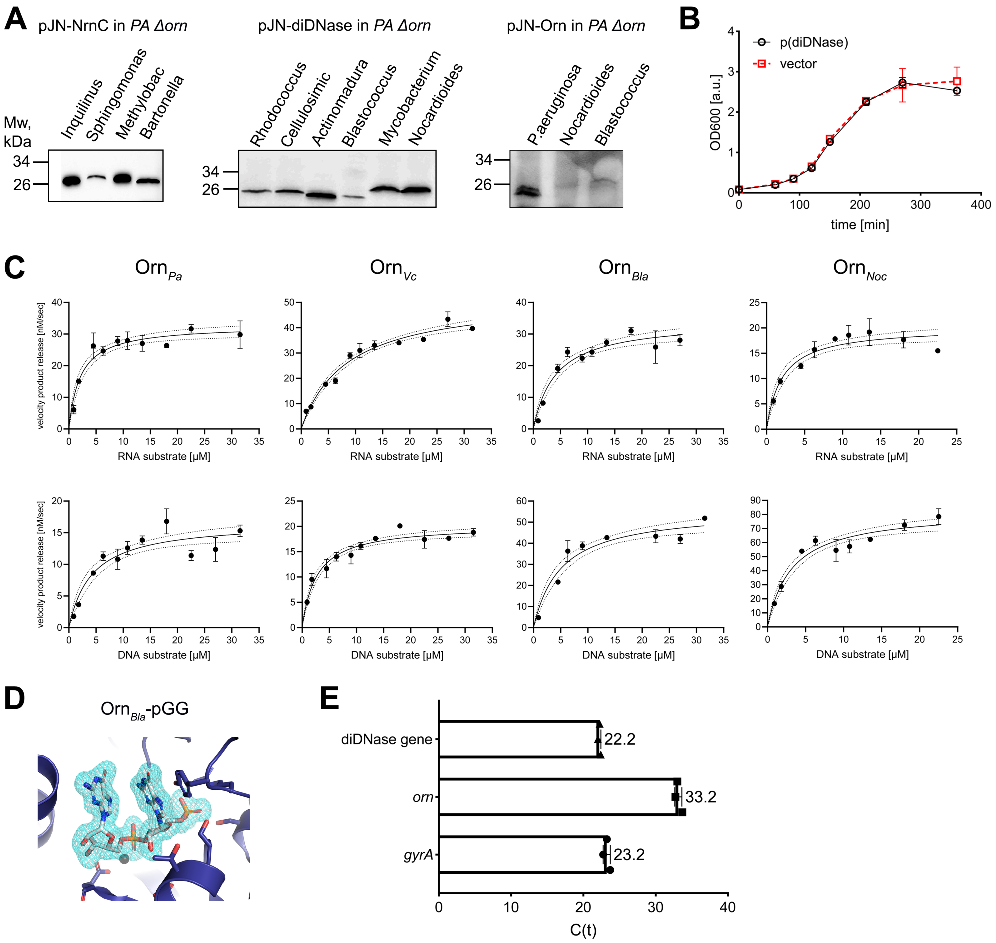

Figure S5. Characterization of Actinomycetal diDNases in vivo and comparison of Actinomycetal and Gammaproteobacterial Orns. **A.** Western blot detection of the indicated StrepII-tagged proteins as expressed from an inducible pJN plasmid in the *P. aeruginosa Δorn* strain. **B**. Growth curves of *P. aeruginosa PA14* wild type harboring either an empty plasmid or plasmid with the *diDNase*$_{Noc}$ gene under induction conditions. Data are average values of three biological replicates and the error bars indicating SDs. **C.** Enzymatic activity of different Orn homologs towards RNA and DNA dinucleotides. Data points are mean values of triplicate experiments fitted using the Michaelis-Menten model. Error bars show SDs and dotted lines indicate the 95% confidence interval of model fitting. **D.** Electron density polder map of the pGG substrate bound to Orn$_{Bla}$.. **E**. Threshold cycle (C(t)) values of qPCR amplification of *diDNase* gene, *orn*, and *gyrA* from cDNA of *Nocardiodies alkalitolerans* grown to mid-exponential phase. Data are averages of three biological replicates and the error bars show SDs. 'No-RT' samples had the following C(t) values: 27.3 ±1.2, *diDNase*; 29.3 ± 0.97, *gyrA*; not detected, *orn*.

Table S1. Representative NrnC homolog sequences from different bacterial orders.

Protein name in the study	Order	Protein ID	Gene locus	Organsim
	Caulobacteriales	WP_010921430.1		
	Emcibacteriales	WP_139941750.1		
	Holosporales	HBN22520.1		
Bartonella	Hyphomicrobiales	WP_011180203.1	NZ_CP082885.1: 131539-132159 (+)	*Bartonella henselae*
Methylobacterium	Hyphomicrobiales	WP_010684307.1	NZ_CP043538.1: 6037200-6037823 (+)	*Methylobacterium mesophilicum*
	Hyphomonadales	MBC6403182.1		
	Iodidimonadales	WP_150004858.1		
	Kordiimonadales	MBT5186728.1		
	Magnetococcales	MBA44506.1		
	Maricaulales	WP_203290785.1		
	Micropepsales	WP_166929412.1		
	Minwuiales	GJL88150.1		
	Parvularculales	WP_173197848.1		
	Rhodobacterales	WP_013654666.1		
Inquilinus	Rhodospirillales	PCJ56693.1	NVWO01000032.1: 27040-27651 (+)	unclassified *Rhodospirillaceae*
	Rhodothalassiales	WP_132708146.1		
	Rickettsiales	WP_010962900.1		
	Sneathiellales	WP_161340238.1		
	Sphingomonadales	WP_315462619.1		
	Futianiales	WP_269333129.1		
	C.Pelagibacterales	MBI05322.1		
	A.incertae sedis	RZO28711.1		
Leptospira	Leptospirales	WP_001128627.1	NC_004342.2: 2888339-2888977 (-)	*Leptospira interrogans* serovar Lai str. 56601
	Chroococcidiopsidales	WP_015154567.1		
	Desertifilales	WP_318700344.1		
	Gloeomargaritales	MBF2096999.1		
	Gloeobacterales	WP_023173952.1		
	Nostocales	MDJ0774554.1		
	Nodosilineales	WP_190701001.1		
Okeania	Oscillatoriales	WP_287241722.1	NZ_JAAHGA010000038.1: 34875-35498 (-)	*Okeania sp*. SIO1H2
	Pleurocapsales	WP_073598865.1		
Thermosynechococcus	Acaryochloridales	WP_011056926.1	NC_004113.1: 1116687-1117322 (+)	*Thermosynechococcus vestitus* BP-1
	Spirulinales	WP_204105276.1		
	Thermostichales	WP_099799022.1		
Synechoccocus	Synechococcales	MBT65949.1	PBYZ01000019.1: 15854-16498 (-)	*Synechococcus sp.* NP17
Nocaridoides	Propionibacteriales	WP_138874963.1	NZ_CP040695.2: 459379-459999 (-)	*Nocardioides sp.* S-1144
	Frankiales	WP_261557234.1		
Blastococcus	Geodermatophilales	WP_097195732.1	NZ_OBQI01000004.1: 182838-183476 (-)	*Blastococcus aggregatus*
	Kitasatosporales	WP_168374667.1		
	Eubacteriales	WP_010965213.1		
Cellulosimicrobium	Micrococcales	WP_144679698.1	NZ_JBBCYN010000007.1: 25516-26157 (+)	*Cellulosimicrobium cellulans*
	Micromonosporales	WP_263023575.1		
Rhodococcus	Mycobacteriales	WP_159105349.1	NZ_BCXE01000001.1: 474556-475197 (-)	*Rhodococcus ruber* NBRC 15591
	Pseudonocardiales	WP_189159268.1		
Actinomadura	Streptosporangiales	WP_165965988.1	NZ_SMKB01000028.1: 18764-19387 (-)	*Actinomadura sp.* 7K534
Clostridium	Eubacteriales	WP_223078741.1	NZ_JAIOKK010000004.1: 85912-86535 (+)	*Clostridium butyricum*

Table S2. X-ray diffraction data processing and refinement statistics.

	diDNaseNoc-dpGG	diDNaseRho	diDNaseRho-dpGG	NrnCBH-dpGG	OrnBla-pGG
PDB ID	9F7G	9F7J	9F7D	9F7L	9F7M
Diffraction data processing					
Resolution range (Å)	40.97 - 1.55 (1.605 - 1.55)	39.28 - 1.72 (1.781 - 1.72)	39.58 - 2.0 (2.071 - 2.0)	47.57 - 2.2 (2.279 - 2.2)	47.28 - 1.65 (1.709 - 1.65)
Space group	C 2 2 21	C 2 2 21	C 2 2 21	P21	P21
Unit cell parameters					
a, b, c (Å), β (º)	35.03, 85.221, 149.056, 90	51.745, 60.353, 134.514, 90	51.837, 61.298, 135.109, 90	71.334, 128.047, 128.71, 94.82	79.57, 117.701, 99.466, 93.617
Unique reflections	32636 (2974)	22757 (2228)	14916 (1457)	115114 (11364)	216829 (21705)
Multiplicity	11.5 (5.3)	13.3 (13.7)	13.2 (13.4)	3.6 (3.5)	6.8 (7.1)
Completeness (%)	98.93 (91.59)	99.78 (99.69)	99.76 (99.73)	98.62 (97.86)	98.98 (99.28)
I/σ (I)	22.03 (4.73)	11.13 (1.90)	14.00 (1.93)	13.36 (1.57)	11.42 (1.66)
Rmeas	0.07804 (0.4277)	0.1822 (2.111)	0.1655 (1.445)	0.07526 (0.9262)	0.1039 (1.251)
CC1/2	0.999 (0.931)	0.997 (0.774)	0.998 (0.865)	0.999 (0.65)	0.997 (0.795)
Refinement					
No. reflections	32633 (2974)	22751 (2228)	14901 (1454)	115086 (11364)	216782 (21706)
No. reflections for Rfree	1908 (173)	1939 (190)	1490 (146)	2006 (197)	1819 (184)
Rwork	0.1434 (0.1528)	0.1778 (0.2865)	0.1836 (0.2696)	0.1758 (0.3128)	0.1709 (0.2852)
Rfree	0.1800 (0.1928)	0.2091 (0.3046)	0.2289 (0.3037)	0.2140 (0.3350)	0.1917 (0.3044)
RMSD bonds (Å)	0.005	0.006	0.007	0.007	0.006
RMSD angles (º)	0.75	0.89	1.04	0.81	0.91
Ramachandran plota :					
Favoured/allowed/outliers (%)	99.01/0.99/0.00	98.97/1.03/0.00	99.02/0.98/0.00	98.89/1.04/0.06	99.20/0.80/0.00
Rotamer outliers (%)	1.12	1.15	0.56	1.26	0.15
Clashscorea	3.35	2.22	3.31	2.17	3.46
Average B-factor (Å2):	19.63	28.32	37.72	50.43	34.44

Statistics for the highest-resolution shell are shown in parentheses

[a] values given by MolProbity

RMSD - root mean square deviation

Table S3. Enzyme kinetic parameters of NrnC homologs. Substrates: RNA - p(2AP)pG, and DNA – p(d2AP)pdG.

Class	Alphaproteobacteria				Cyanobacteria				Spirochaetia	
Protein	Bartonella		Inquilinus		Synechococcus		Okeania		Leptospira	
Substrate	RNA	DNA	RNA	DNA	RNA	DNA	RNA	DNA	RNA	DNA
Enzyme, nM	10	10	10	10	10	10	10	10	10	10
Vmax, nM/sec	217.00	44.51	78.88	29.99	9.34	13.27	78.54	80.29	70.66	21.17
Km, µM	9.14	2.89	8.77	3.35	2.68	2.05	8.82	5.24	9.68	4.32
kcat, s^{-1}	21.70	4.45	7.89	3.00	0.93	1.33	7.85	8.03	7.07	2.12
kcat/Km, $*10^6$ M^{-1} s^{-1}	1.14	0.46	0.90	0.90	0.35	0.65	0.89	1.53	0.73	0.49
DNA:RNA	0.40		1.00		1.86		1.72		0.67	

Class	Actinomycetes								Clostridia	
Protein	Rhodococcus		Blastococcus		Nocardioides		Cellulosimic.		Clostridium	
Substrate	RNA	DNA	RNA	DNA	RNA	DNA	RNA	DNA	RNA	DNA
Enzyme, nM	500	10	500	10	500	10	500	10	500	10
Vmax, nM/sec	90.75	45.47	15.85	68.35	134.60	19.23	426.10	265.50	480.70	265.50
Km, µM	84.30	9.25	39.39	20.59	125.60	7.62	66.66	21.68	67.52	21.68
kcat, s^{-1}	0.18	4.55	0.03	6.84	0.27	1.92	0.85	26.55	0.96	26.55
kcat/Km, $*10^6$ M^{-1} s^{-1}	0.0022	0.49	0.0008	0.33	0.0021	0.25	0.0130	1.22	0.0140	1.22
DNA:RNA	222.72		412.48		119.05		93.85		87.14	

Table S4. Kinetic parameters of diDNase$_{Noc}$ towards different substrates.

	RNA		DNA			RNA-DNA	DNA-RNA
	p(2AP)pG	pGp(2AP)	p(d2AP)pdG,	p(d2AP)pdC,	pdCp(d2AP),	pGp(d2AP)	p(d2AP)pG
	R-R	R-R	R-R	R-Y	Y-R	R-R	R-R
Enzyme, nM	500	500	10	10	10	500	10
Vmax, nM/sec	134.60	225.80	19230.00	225.20	304.90	200.10	244.5
Km, µM	125.60	92.98	7620.00	13.59	28.52	45.92	33.61
kcat, s^{-1}	0.27	0.45	1923.00	22.52	30.49	0.40	24.45
kcat/Km, $*10^6$ M^{-1} s^{-1}	0.002	0.005	0.252	1.660	1.070	0.009	0.728
Ki, µM	-	-	-	21.53	12.58	-	-

Table S5. Enzyme kinetics parameters of $diDNase_{Noc}$ and $NrnC_{Bh}$ mutants.

	$diDNase_{Noc}$						$NrnC_{Bh}$					
	$F^{88}I$		$H^{81}F/Y^{206}H$		$H^{81}F/Y^{206}H/F^{88}I$		$I^{87}F$		$F^{80}H/H^{205}Y$		$F^{80}H/H^{205}Y/I^{87}F$	
	RNA	DNA	RNA	DNA	RNA	DNA	RNA	DNA	RNA	DNA	RNA	DNA
Enzyme, nM	500	500	500	10	500	500	10	10	10	10	10	10
Vmax, nM/sec	54.47	52.72	15.74	231.80	3.33	14.48	106.00	9.47	125.80	12.75	125.00	9225.00
Km, μM	161.90	67.87	22.28	228.00	21.35	45.28	18.25	0.64	8355.00	0.36	6247.00	0.49
kcat, s^{-1}	0.1089	0.1054	0.0314	23.18	0.0067	0.0289	10.60	0.83	12.58	1275.00	12.50	0.9225
kcat/Km, $*10^6$ M^{-1} s^{-1}	0.0007	0.0016	0.0014	0.1017	0.0003	0.0006	0.5808	0.26	1.51	3.56	2.00	1.87
DNA:RNA	2.31		72.00		2.06		0.45		2.36		0.94	

Table S6. Enzyme kinetics parameters of Orn homologs.

	Orn_{Vc}		Orn_{Pa}		Orn_{Bla}		Orn_{Noc}	
	RNA	DNA	RNA	DNA	RNA	DNA	RNA	DNA
Enzyme, nM	10	10	5	10	10	100	10	100
Vmax, nM/sec	52.20	20.29	32.76	16.77	34.24	55.99	33.38	82.78
Km, µM	8.47	2.68	2.11	4.26	4.14	5.03	5.27	3.20
kcat, s^{-1}	5.22	2.03	6.55	1.68	3.42	0.56	3.34	0.83
kcat/Km, $*10^6\ M^{-1}\ s^{-1}$	0.62	0.76	3.11	0.39	0.83	0.11	0.63	0.26
DNA:RNA	1.23		0.13		0.13		0.41	

Table S7 - partA. Details of the genomic environment analysis.

DiDNase protein ID	Genome assembly ID	Species	Order	Mobilome genes	Inside prophage	Known/predicted phage defense systems (Number of genes in a system)	Multidrug Resistance MFS transporter	Toxin-Antitoxin systems
WP_138874963.1	GCF_005954645.2	Nocardioides sp. S-1144	Propionibacteriales		no	Gao19 (2), PDC-S30 (1)		
WP_084540974.1	GCF_000426525.1	Nocardioides alkalitolerans	Propionibacteriales	recombinase family protein WP_052336343.1	yes	no		
WP_210650311.1	GCF_017916425.1	Nocardioides sp. SYSU D00065	Propionibacteriales		no	no		
WP_212324208.1	GCF_018128325.1	Arachnia rubra	Propionibacteriales	transposase family protein WP_212323263.1; IS3 family transposase WP_223927909.1; ISAs1 family transposase WP_212324177.1; ISAs1 family transposase WP_244980176.1; transposase family protein WP_212324175.1; transposase WP_244980175.1; transposase WP_212324228.1; WP_223927697.1 transposase family protein; WP_244980173.1 transposase family protein; WP_244980174.1 transposase; WP_244980178.1 transposase; WP_263407059.1 transposase; WP_280526541.1 transposase	yes	PDC-M03 (2)		type II toxin-antitoxin system Phd/YefM family antitoxin WP_212324212.1; type II toxin-antitoxin system Phd/YefM family toxin WP_212324214.1
WP_185973153.1	GCF_007421815.1	Aeromicrobium piscarium	Propionibacteriales	IS3 family transposase WP_223927909.1	no	PDC-M03 (2), DarTG (2)		
WP_082574769.1	GCF_001428725.1	unclassified Nocardioides	Propionibacteriales		no	PDC-M03 (2)	three, WP_056154328.1, WP_056154344.1, WP_056154321.1	
WP_259808649.1	GCF_025144225.1	Aestuariimicrobium sp. p3-SID1156	Propionibacteriales		no	PDC-M03 (2)	one, WP_259808659.1	
WP_165489951.1	GCF_004324755.1	Propioniciclava sinopodophylli	Propionibacteriales	tyrosine-type recombinase/integrase WP_131166526.1	no	RM type I (3)		
WP_266515825.1	GCF_026342455.1	Streptomyces canus	Kitasatosporales	mobile element transfer protein WP_046708309.1; WP_266515792.1 site-specific integrase ; replication initiator protein RepSA WP_266515797.1	no	no		
WP_234539034.1	GCF_021462265.1	Streptomyces shenzhenensis	Kitasatosporales	mobile element transfer protein WP_234538975.1; site-specific integrase WP_234538964.1; replication initiator protein RepSA WP_234538967.1; IS110 family transposase WP_234539033.1	no	no		
WP_189481977.1	GCF_014649895.1	Streptomyces rubiginosus	Kitasatosporales	mobile element transfer protein WP_189481966.1; site-specific integrase WP_189481959.1; replication initiator protein RepSA WP_189481961.1	no	no		
WP_312004126.1	GCF_031932565.1	Streptomyces sp. B1866	Kitasatosporales		no	no		
WP_161302357.1	GCF_900091855.1	Streptomyces sp. DvalAA-14	Kitasatosporales		no	AbiU (1)		
WP_237555091.1	GCF_009863045.1	Streptomyces sp. SID4948	Kitasatosporales		no	AbiU (1)		
WP_189798739.1	GCF_021394575.1	Streptomyces thermodiastaticus	Kitasatosporales	transposase WP_233975594.1	no	no		
WP_079133910.1	GCF_001746305.1	Streptomyces sp. EN23	Kitasatosporales		no	no		type II toxin-antitoxin system CcdA family antitoxin WP_069740776.1; nucleotidyl transferase AbiEii/AbiGii toxin family protein WP_069740780.1
WP_266520503.1	GCF_026341955.1	Streptomyces sp. NBC_00474	Kitasatosporales	transposase WP_266518740.1; IS5 family transposase WP_266520501.1; IS5 family transposase WP_266518733.1; WP_266520196.1 IS701 family transposase; WP_266520501.1 IS5 family transposase	no	no		type II toxin-antitoxin system PemK/MazF family toxin WP_266512598.1
WP_168374667.1	GCF_012328665.1	Streptomyces galbus	Kitasatosporales	mobile element transfer protein WP_137961317.1; site-specific integrase WP_168374678.1; replication initiation protein WP_168374676.1;	no	no		
WP_323178798.1	GCF_026341855.1	Streptomyces sp. NBC_00568	Kitasatosporales	IS5 family transposase WP_266755043.1	yes	no		
WP_266761021.1	GCF_026341795.1	Streptomyces sp. NBC_00638	Kitasatosporales	IS5 family transposase WP_266755043.1	yes	no		
WP_201054839.1	GCF_016654115.1	Streptomyces sp. MBT53	Kitasatosporales	IS3 family transposase (frame-shifted)	no	PD-Lambda-1 (1)		
WP_266811478.1	GCF_026341895.1	Streptomyces longwoodensis	Kitasatosporales	site-specific integrase WP_266811480.1; phage/plasmid primase, P4 family WP_266811484.1	yes	no	two, WP_266811468.1, WP_266814572.1	
WP_071659270.1	GCF_000816485.1	Streptomyces sp. MUSC 125	Kitasatosporales	mobile element transfer WP_039654752.1 ; site-specific integrase WP_039654747.1; replication initiator WP_039654749.1	no	no		
WP_280911787.1	GCF_029894045.1	Streptomyces sp. SAI-208	Kitasatosporales	mobile element transfer protein WP_280911794.1; site-specific integrase WP_246178598.1	no	no		
WP_057608706.1	GCF_001425805.1	Streptomyces sp. Root369	Kitasatosporales	mobile element transfer protein WP_057608713.1; IS3 family transposase WP_057608698.1; replication initiator WP_057608716.1	no	no		
WP_261557234.1	GCF_025403405.1	Frankia tisae	Frankiales		no	PDC-M03 (2), PD-T4-6 (1)	one, WP_261557239.1	type II toxin-antitoxin system PemK/MazF family toxin WP_261557212.1
WP_251747721.1	GCF_023716945.1	Frankia sp. AiPs1	Frankiales		no	PDC-M03 (2)	one, WP_251747724.1	
WP_198152831.1	GCF_000966285.1	Pseudofrankia sp. DC12	Frankiales		no	no	one, WP_045876247.1	
WP_315912562.1	GCF_032460525.1	Geodermatophilus sp. DSM 44513	Geodermatophilales		no	no		
WP_097195732.1	GCF_900221005.1	Blastococcus aggregatus	Geodermatophilales	recombinase family protein WP_245852818.1	no	no		
WP_166486422.1	GCF_000284015.1	Blastococcus saxobsidens	Geodermatophilales		no	PDC-M03 (2)		
WP_134352466.1	GCF_004402135.1	Microbacterium sp. 3H14	Micrococcales	site-specific integrase WP_134352458.1	no	no	one, WP_134352454.1	
WP_134850835.1	GCF_004535805.1	Cellulomonas sp. HD19AZ1	Micrococcales	recombinase family protein WP_134850833.1; recombinase family protein WP_206119940.1	no	RM_type_IIG (1), Wadjet type III(4), PDC-S09 (1)		
WP_199424253.1	GCF_016464425.1	Actinotalea solisilvae	Micrococcales		no	no		
WP_203676524.1	GCF_016862775.1	Cellulomonas phragmiteti	Micrococcales	tyrosine-type recombinase/integrase WP_203676491.1	no	no		type IV toxin-antitoxin system AbiEi family antitoxin
WP_051681776.1	GCF_000708885.1	Cellulomonas sp. HZM	Micrococcales		no	no		
WP_185275394.1	GCF_014217625.1	Leifsonia shinshuensis	Micrococcales	type IV secretory system conjugative DNA transfer family protein WP_258045887.1	no	no		
WP_246906846.1	GCF_023015685.1	Isoptericola sp. S6320L	Micrococcales		no	no		
WP_252593457.1	GCF_024519215.1	Ornithinimicrobium	Micrococcales	transposase family protein WP_256843042.1; ISAs1 family transposase WP_306239619.1; transposase family protein WP_256843040.1	no	no		
WP_154346788.1	GCF_009674665.1	Agromyces kandeliae	Micrococcales		no	RM type II (2), Vspr (1), HEC-06 (1)		
WP_259308335.1	GCF_024704385.1	Cellulomonas sp. P24	Micrococcales		no	DMS_others (no pseudogenes, 3 genes: MTase II, specificity I, REease I), PDC-S08 (1), PDC-M03 (2)		
WP_048342045.1	GCF_001040865.1	Cellulomonas sp. A375-1	Micrococcales	tyrosine-type recombinase/integrase WP_197080755.1	no	RM type III (2), PDC-M03 (2)		toxin-antitoxin system HicB family antitoxin WP_048342051.1
WP_144679698.1	GCF_007680565.1	Cellulosimicrobium sp. TH-20	Micrococcales		no	HEC-05 (1), PCD-M03, DMS_others (2)		
WP_221632387.1	GCF_006716205.1	Oryzihumus leptocrescens	Micrococcales	site-specific integrase WP_141786868.1; plasmid replication, integration and excision activator WP_221632386.1; replication initiation protein WP_141786870.1	no	PDC-M03 (2)		
WP_147794797.1	GCF_008033115.1	Cellulomonas sp. Y8	Micrococcales		no	no		
WP_147918740.1	GCF_008079355.1	Ruania zhangjianzhongii	Micrococcales		no	no		
WP_168168536.1	GCF_001816045.1	Kytococcus sp. HMSC28H12	Micrococcales	transposase WP_009481982.1, plasmid stabilization protein OFS06195.1=type II toxin-antitoxin system VapC family toxin WP_070706162.1	no	no		type IV toxin-antitoxin system AbiEi family antitoxin domain-containing protein; type II toxin-antitoxin system VapC family toxin WP_070706162.1
WP_197522505.1	GCF_902703175.1	Occultella aeris	Micrococcales		no	PDC-M03 (2)		
WP_205653545.1	GCF_017052465.1	Arthrobacter pascens	Micrococcales	ISL3 family transposase WP_205653546.1	no	BREX type II (6), RM type IV (1), PDC-M03 (2)		
WP_310112464.1	GCF_031455825.1	Pseudarthrobacter oxydans	Micrococcales		no	PDC-M03 (2), RM type IV (1)		
WP_096288639.1	GCF_002332385.1	Glutamicibacter sp. BW80	Micrococcales		no	PDC-M03 (2)		
WP_283319023.1	GCF_030053835.1	Cellulomonas sp. ES6	Micrococcales		no	PDC-M03 (2)		zeta toxin family protein WP_283319035.1
WP_128470519.1	GCF_004100365.1	Glutamicibacter sp. HZAU	Micrococcales		no	PDC-M03 (2)		
WP_259815455.1	GCF_025144125.1	Brachybacterium paraconglomeratum	Micrococcales	tyrosine recombinase XerC WP_126984584.1	no	PDC-M03 (2) RM type I (3)		
WP_320965088.1	GCF_034119785.1	Glutamicibacter protophormiae	Micrococcales		no	PDC-M03 (2)		
WP_308739417.1	GCF_030866705.2	Brachybacterium sp. GU-2	Micrococcales		no	PDC-M03 (2) RM type I (3)		
WP_056915130.1	GCF_001427915.1	Phycicoccus sp. Root563	Micrococcales	tyrosine recombinase XerC WP_235529051.1; recombinase family protein WP_235530755.1	no	HEC-06 (1)		
WP_259843041.1	GCF_025152195.1	Brachybacterium muris	Micrococcales		no	PDC-M03 (2)		
WP_278177389.1	GCF_029626175.1	Micromonospora sp. WMMD1082	Micromonosporales	IS1634 family transposase WP_278176203.1	no	no		
WP_269689498.1	GCF_027460165.1	Micromonospora sp. WMMC241	Micromonosporales		no	no		
WP_263023575.1	GCF_025630775.1	Actinoplanes sp. KI2	Micromonosporales		no	no		
WP_213011332.1	GCF_018332695.1	Actinoplanes toevensis	Micromonosporales		no	DMS_others (2 : mREase II, REase II)		
WP_168214987.1	GCF_008329905.1	Mycobacterium sp. ELW1	Mycobacteriales	site-specific integrase WP_234787735.1; DDE-type integrase/transposase/recombinase WP_149382908.1	yes	no		
WP_068262908.1	GCF_001667625.1	Mycobacterium sp. E1715	Mycobacteriales		no	no		
WP_225503931.1	GCF_020227715.1	Mycolicibacterium fortuitum	Mycobacteriales		yes	no		
WP_063061358.1	GCF_001613205.1	Nocardia sienata	Mycobacteriales	tyrosine-type recombinase/integrase WP_063061366.1, DDE-type integrase/transposase/recombinase, Tn3 family transposase, IS3 family transposase, DDE-type integrase/transposase/recombinase	no	no		
WP_029108576.1	GCF_000426065.1	Mycobacterium sp. URHD0025	Mycobacteriales	site-specific integrase WP_029108587.1	no	no		
WP_184443023.1	GCF_014202335.1	Mycobacterium sp. AZCC_0083	Mycobacteriales		no	no		
WP_067179195.1	GCF_001673415.1	Mycobacterium sp. 1165196.3	Mycobacteriales		no	no		
WP_069400067.1	GCF_001722325.1	Mycobacterium sherrisii	Mycobacteriales		yes	no		
WP_264721898.1	GCF_025989245.1	Rhodococcus pyridinivorans	Mycobacteriales	site-specific integrase WP_264721896.1	no	no	one, WP_264721889.1	

WP_007631577.1	GCF_004346635.1	Dietzia cinnamea	Mycobacteriales	IS630 family transposase WP_081470573.1	no	PDC-M03 (2)	one, WP_243699659.1	
WP_159105349.1	GCF_003086595.1	Rhodococcus ruber	Mycobacteriales	transposase family protein WP_072786302.1	yes	DndABCDE (5), PbeABCD (4)		
WP_010694450.1	GCF_000194155.1	Saccharopolyspora spinosa	Pseudonocardiales	IS21 family transposase WP_202798806.1	no	RM type IIG (1), Mokosh type II (1) RM type IV (1) 10 kbp/13 genes away		Scr1 family TA system antitoxin-like transcriptional regulator WP_237710510.1
WP_283663879.1	GCF_030064675.1	Crossiella sp. CA-258035	Pseudonocardiales	plasmid replication, integration and excision activator WP_283663883.1; replication initiator protein WP_283666125.1; tyrosine-type recombinase/integrase WP_283663889.1	no	no		
WP_189159268.1	GCF_014646255.1	Lentzea pudingi	Pseudonocardiales		no	RM type I (3)		
WP_180903687.1	GCF_002850745.1	Nonomuraea indica	Streptosporangiales	site-specific integrase WP_281260300.1; plasmid replication, integration and excision activator WP_101788513.1	no	no		
WP_223830488.1	GCF_008638365.1	Nocardiopsis quinghaiensis	Streptosporangiales		no	no	one, WP_150241378.1	
WP_165965988.1	GCF_004348575.1	Actinomadura sp. 7K534	Streptosporangiales	plasmid replication, integration and excision activator WP_132045620.1; replication initiation protein WP_243718312.1; tyrosine-type recombinase/integrase WP_132045616.1	no	no		
WP_250358789.1	GCF_023614255.1	Actinomadura madurae	Streptosporangiales	plasmid replication, integration and excision activator WP_250358784.1; site-specific integrase WP_250358780.1	no	no		
WP_148758586.1	GCF_008121305.1	Actinomadura decatromicini	Streptosporangiales	site-specific integrase WP_246178598.1	no	Shedu (1)		
WP_161111101.1	GCF_009862005.1	Nocardiopsis alba	Streptosporangiales		no	PDC-M03 (2)	one, WP_161111106.1	
WP_110050910.1	GCF_003182025.1	Nocardiopsis sp. L17-MgMaSL7	Streptosporangiales		no	no		
QKW32475.1	GCA_013364295.1	Nocardiopsis flavescens NA01583	Streptosporangiales	plasmid	no	PDC-M03 (2)		
WP_168932123.1	GCF_012843415.1	Paraclostridium bifermentans	Eubacteriales	recombinase family protein WP_168932136.1	no	no		
WP_118056553.1	GCF_003461105.1	unclassified Roseburia	Eubacteriales	transposase WP_118056556.1	no	Mokosh_TypeII (1), AbiO-Nhi_family (1), PDC-S51 (1), Lamassu_Family (3), PDC-S38 (1)		
WP_136002637.1	GCF_019710775.1	Clostridium perfringens QHY-2	Eubacteriales	RusA family crossover junction endodeoxyribonuclease WP_136002631.1	yes	no		
WP_010965213.1	GCF_000191905.1	Clostridium acetobutylicum EA 2018	Eubacteriales		yes	no		
WP_312368662.1	GCF_031997925.1	Lachnoclostridium sp.	Eubacteriales	IS3 family transposase WP_312368650.1	yes	RM type I (3), SoFic (1)		type II toxin-antitoxin system prevent-host-death family antitoxin WP_312368789.1
WP_223078741.1	GCF_019913025.1	Clostridium butyricum GD1_1	Eubacteriales	site-specific integrase WP_316301477.1	no	no		
WP_118375161.1	GCF_003474775.1	Agathobacter rectalis	Eubacteriales		no	no		
WP_242872456.1	GCF_900002825.1	Romboutsia lituseburensis	Eubacteriales	RusA family crossover junction endodeoxyribonuclease WP_092727753.1	yes	no		
WP_255847331.1	GCF_024397055.1	Clostridium butyricum LCL-155_viral	Eubacteriales	tyrosine-type recombinase/integrase WP_255847336.1	yes	no		
93<br>100%				39 not part of prophage<br>total MGE: 53	14	39<br>41.90%	11<br>11.80%	10<br>10.80%

Table S7 - part B. Details of the genomic environment analysis.

Orn or NrnA protein ID	Genome assembly ID	Species	Order	Mobilome genes	Known/predicted phage defense systems (Number of genes in a system)
WP_246060552.1	GCF_005954645.2	Nocardioides sp. S-1144	Propionibacteriales		
WP_043642046.1	GCF_000426525.1	Nocardioides alkalitolerans	Propionibacteriales		
WP_210648821.1	GCF_017916425.1	Nocardioides sp. SYSU D00065	Propionibacteriales		
WP_212321617.1	GCF_018128325.1	Arachnia rubra	Propionibacteriales		
WP_143913339.1	GCF_007421815.1	Aeromicrobium piscarium	Propionibacteriales		
WP_056153707.1	GCF_001428725.1	unclassified Nocardioides	Propionibacteriales		
WP_259809485.1	GCF_025144225.1	Aestuariimicrobium sp. p3-SID1156	Propionibacteriales	recombinase family protein WP_259809486.1	
WP_131167292.1	GCF_004324755.1	Propioniciclava sinopodophylli	Propionibacteriales	site-specific integrase WP_131167287.1	
WP_057609681.1	GCF_026342455.1	Streptomyces canus	Kitasatosporales		
WP_234535545.1	GCF_021462265.1	Streptomyces shenzhenensis	Kitasatosporales		
WP_006131557.1	GCF_014649895.1	Streptomyces rubiginosus	Kitasatosporales		
WP_312007930.1	GCF_031932565.1	Streptomyces sp. B1866	Kitasatosporales		
WP_093739063.1	GCF_900091855.1	Streptomyces sp. DvalAA-14	Kitasatosporales		
WP_093739063.1	GCF_009863045.1	Streptomyces sp. SID4948	Kitasatosporales		
WP_189799164.1	GCF_021394575.1	Streptomyces thermodiastaticus	Kitasatosporales		
WP_069739578.1	GCF_001746305.1	Streptomyces sp. EN23	Kitasatosporales		
WP_266514961.1	GCF_026341955.1	Streptomyces sp. NBC_00474	Kitasatosporales	MULTISPECIES: IS200/IS605 family transposase WP_266514974.1; transposase WP_266520282.1	
WP_137301343.1	GCF_012328665.1	Streptomyces galbus	Kitasatosporales		
WP_266759064.1	GCF_026341855.1	Streptomyces sp. NBC_00568	Kitasatosporales		
WP_266759064.1	GCF_026341795.1	Streptomyces sp. NBC_00638	Kitasatosporales		
WP_200712221.1	GCF_016654115.1	Streptomyces sp. MBT53	Kitasatosporales		
WP_137307006.1	GCF_026341895.1	Streptomyces longwoodensis	Kitasatosporales		
WP_039649274.1	GCF_000816485.1	Streptomyces sp. MUSC 125	Kitasatosporales		
WP_280894676.1	GCF_029894045.1	Streptomyces sp. SAI-208	Kitasatosporales		
WP_057609681.1	GCF_001425805.1	Streptomyces sp. Root369	Kitasatosporales		
WP_261559525.1	GCF_025403405.1	Frankia tisae	Frankiales		
WP_285902040.1	GCF_023716945.1	Frankia sp. AiPs1	Frankiales		
WP_157488974.1	GCF_000966285.1	Pseudofrankia sp. DC12	Frankiales		
WP_315912377.1	GCF_032460525.1	Geodermatophilus sp. DSM 44513	Geodermatophilales		
WP_097194003.1	GCF_900221005.1	Blastococcus aggregatus	Geodermatophilales		
WP_231839556.1	GCF_000284015.1	Blastococcus saxobsidens	Geodermatophilales	IS4 family transposase WP_014375120.1	
WP_131490977.1	GCF_004402135.1	Microbacterium sp. 3H14	Micrococcales		
WP_255430542.1	GCF_004535805.1	Cellulomonas sp. HD19AZ1	Micrococcales		
WP_199423943.1	GCF_016464425.1	Actinotalea solisilvae	Micrococcales		
WP_203670382.1	GCF_016862775.1	Cellulomonas phragmiteti	Micrococcales		
WP_029289528.1	GCF_000708885.1	Cellulomonas sp. HZM	Micrococcales		
WP_185277595.1	GCF_014217625.1	Leifsonia shinshuensis	Micrococcales		
WP_246910438.1	GCF_023015685.1	Isoptericola sp. S6320L	Micrococcales		
WP_256841199.1	GCF_024519215.1	Ornithinimicrobium	Micrococcales		
WP_154347597.1	GCF_009674665.1	Agromyces kandeliae	Micrococcales		
WP_259307157.1	GCF_024704385.1	Cellulomonas sp. P24	Micrococcales		
WP_048342615.1	GCF_001040865.1	Cellulomonas sp. A375-1	Micrococcales		
WP_144680238.1	GCF_007680565.1	Cellulosimicrobium sp. TH-20	Micrococcales		
WP_221632431.1	GCF_006716205.1	Oryzihumus leptocrescens	Micrococcales		
WP_147797185.1	GCF_008033115.1	Cellulomonas sp. Y8	Micrococcales		
WP_268957778.1	GCF_008079355.1	Ruania zhangjianzhongii	Micrococcales		
WP_083323510.1	GCF_001816045.1	Kytococcus sp. HMSC28H12	Micrococcales		
WP_197522623.1	GCF_902703175.1	Occultella aeris	Micrococcales		
WP_205654066.1	GCF_017052465.1	Arthrobacter pascens	Micrococcales		
WP_174181848.1	GCF_031455825.1	Pseudarthrobacter oxydans	Micrococcales	IS110 family transposase WP_174179957.1	
WP_096288791.1	GCF_002332385.1	Glutamicibacter sp. BW80	Micrococcales		
WP_283320822.1	GCF_030053835.1	Cellulomonas sp. ES6	Micrococcales		
WP_128470343.1	GCF_004100365.1	Glutamicibacter sp. HZAU	Micrococcales		
WP_259813874.1	GCF_025144125.1	Brachybacterium paraconglomeratum	Micrococcales		
WP_320965259.1	GCF_034119785.1	Glutamicibacter protophormiae	Micrococcales	IS1380 family transposase WP_320965253.1	
WP_308738557.1	GCF_030866705.2	Brachybacterium sp. GU-2	Micrococcales		
WP_255354156.1	GCF_001427915.1	Phycicoccus sp. Root563	Micrococcales	tyrosine-type recombinase/integrase WP_082581073.1	
no Orn	GCF_025152195.1	Brachybacterium muris	Micrococcales		
WP_278175641.1	GCF_029626175.1	Micromonospora sp. WMMD1082	Micromonosporales		
WP_091063613.1	GCF_027460165.1	Micromonospora sp. WMMC241	Micromonosporales		
WP_263021849.1	GCF_025630775.1	Actinoplanes sp. KI2	Micromonosporales		
WP_213011482.1	GCF_018332695.1	Actinoplanes toevensis	Micromonosporales		
WP_168214822.1	GCF_008329905.1	Mycobacterium sp. ELW1	Mycobacteriales	site-specific integrase WP_149378834.1	
WP_067760557.1	GCF_001667625.1	Mycobacterium sp. E1715	Mycobacteriales		
WP_003880032.1	GCF_020227715.1	Mycolicibacterium fortuitum	Mycobacteriales		
WP_063060840.1	GCF_001613205.1	Nocardia sienata	Mycobacteriales		
WP_029110086.1	GCF_000426065.1	Mycobacterium sp. URHD0025	Mycobacteriales	IS30 family transposase, partial WP_029110088.1	
WP_184443618.1	GCF_014202335.1	Mycobacterium sp. AZCC_0083	Mycobacteriales		
WP_066860339.1	GCF_001673415.1	Mycobacterium sp. 1165196.3	Mycobacteriales		
WP_069398832.1	GCF_001722325.1	Mycobacterium sherrisii	Mycobacteriales		
WP_024100533.1	GCF_025989245.1	Rhodococcus pyridinivorans	Mycobacteriales		
WP_007626700.1	GCF_004346635.1	Dietzia cinnamea	Mycobacteriales		
WP_010596338.1	GCF_003086595.1	Rhodococcus ruber	Mycobacteriales		
WP_029535366.1	GCF_000194155.1	Saccharopolyspora spinosa	Pseudonocardiales		
WP_283663409.1	GCF_030064675.1	Crossiella sp. CA-258035	Pseudonocardiales	tyrosine-type recombinase/integrase WP_283663407.1	RM type I (3)
WP_189158437.1	GCF_014646255.1	Lentzea pudingi	Pseudonocardiales		
WP_101787138.1	GCF_002850745.1	Nonomuraea indica	Streptosporangiales		
WP_150242296.1	GCF_008638365.1	Nocardiopsis quinghaiensis	Streptosporangiales	site-specific integrase WP_223830497.1	
WP_132049030.1	GCF_004348575.1	Actinomadura sp. 7K534	Streptosporangiales		
WP_250357390.1	GCF_023614255.1	Actinomadura madurae	Streptosporangiales		
WP_148757160.1	GCF_008121305.1	Actinomadura decatromicini	Streptosporangiales		
WP_017534257.1	GCF_009862005.1	Nocardiopsis alba	Streptosporangiales	tyrosine-type recombinase/integrase WP_326338221.1;	RM type I (3)
WP_026129118.1	GCF_003182025.1	Nocardiopsis sp. L17-MgMaSL7	Streptosporangiales		
no Orn	GCA_013364295.1	Nocardiopsis flavescens NA01583	Streptosporangiales		
WP_025162354.1	GCF_012843415.1	Paraclostridium bifermentans	Eubacteriales		
WP_117841001.1	GCF_003461105.1	unclassified Roseburia	Eubacteriales		
WP_003459795.1	GCF_019710775.1	Clostridium perfringens QHY-2	Eubacteriales		
WP_010965110.1	GCF_000191905.1	Clostridium acetobutylicum EA 2018	Eubacteriales		
WP_312372351.1	GCF_031997925.1	Lachnoclostridium sp.	Eubacteriales		
WP_024038962.1	GCF_019913025.1	Clostridium butyricum GD1_1	Eubacteriales		
WP_015569395.1	GCF_003474775.1	Agathobacter rectalis	Eubacteriales		
WP_092724658.1	GCF_900002825.1	Romboutsia lituseburensis	Eubacteriales		
WP_002579640.1	GCF_024397055.1	Clostridium butyricum LCL-155_viral	Eubacteriales		
91				12	2
100%				13.20%	2.20%
